# Supplementary material for: Indications of alcohol or drug use disorders in five different national registers in Sweden: a cross-sectional population-based study
Source: BMJ Open. 2023 Sep 4;13(9):e070744. doi: 10.1136/bmjopen-2022-070744 (PMC10481836; doi:10.1136/bmjopen-2022-070744)
Supplement: Supplementary data [file bmjopen-2022-070744supp001.pdf]

Supplementary tables

Supplementary table 1 Alcohol use disorder in the male population. Total number (N) with indication, percent % and number per cell (n) with register indication by age-group, migrant status, education, marital status, phi - the correlation coefficient between the registers and the unique contribution of the specific register to all registers combined (Any registers) for the following registers: Inpatient care, Outpatient care, the Register of Medications (Medications), the Social Insurance register (Social Insurance), the Register of convictions (Convictions), and Any register.

		**Inpatient**	**Outpatient**	**Medication**	**Social Insurance**	**Convictions**	**Any registers**
		N=46889	N=65445	N=71680	N=6415	N=1642	N=114789
		% (n)	% (n)	% (n)	% (n)	% (n)	% (n)
**Age**	**20–29**	1.09 (3627)	1.87 (6209)	1.45 (4821)	0.08 (259)	0.08 (251)	3.16 (10530)
	**30–39**	1.01 (6033)	1.73 (10297)	1.83 (10881)	0.15 (898)	0.06 (333)	2.98 (17699)
	**40–49**	1.69 (11050)	2.49 (16257)	2.90 (18887)	0.31 (2038)	0.07 (441)	4.26 (27768)
	**50–59**	2.37 (13889)	3.08(18097)	3.52 (20678)	0.40 (2320)	0.07 (396)	5.38 (31563)
	**60–65**	2.05 (12290)	2.43 (14585)	2.74 (16413)	0.15 (900)	0.04 (221)	4.54 (27229)
**Migration status**	**Born in Sweden**	1.73 (40370)	2.40 (55988)	2.74(63916)	0.25 (5748)	0.06 (1345)	4.27 (99770)
	**Born abroad**	1.63 (6133)	2.34 (8798)	1.94 (7295)	0.17 (632)	0.07 (275)	3.71 (13944)
	**Refugee**	0.69 (386)	1.18 (659)	0.84 (469)	0.06 (35)	0.04 (22)	1.92 (1075)
**Education**	**Education Pre-upper**	3.03 (15314)	3.88 (19623)	3.98 (20116)	0.33 (1671)	0.14 (687)	6.75 (34084)
	**Upper second**	1.83 (24993)	2.51 (34331)	2.79 (38145)	0.28 (3774)	0.06 (802)	4.43 (60676)
	**Post-second**	0.71 (6023)	1.28 (10749)	1.52 (12833)	0.11 (947)	0.01 (115)	2.23 (18772)
	**Miss**	1.14 (559)	1.51 (742)	1.19 (586)	0.05 (23)	0.08 (38)	2.56 (1257)
**Marital status**	**Married**	0.84 (9605)	1.29 (14734)	1.85 (21070)	0.13 (1453)	0.02 (267)	2.58 (29405)
	**Unmarried**	1.89 (24986)	2.69 (35455)	2.64 (34762)	0.25 (3311)	0.08 (992)	4.52 (59662)
	**Divorced**	4.07 (11824)	5.04 (14650)	5.22 (15183)	0.55 (1602)	0.13 (369)	8.48 (24635)
	**Widow(er)**	2.77 (474)	3.54 (606)	3.89 (665)	0.29 (49)	0.08 (14)	6.36 (1087)
**Unique contribution to Any register**	**%**	8.74 (10037)	16.55 (19001)	27.57 (31653)	0.46 (531)	0.19 (98)	-

Lundin A, *et al. BMJ Open* 2023; 13:e070744. doi: 10.1136/bmjopen-2022-070744

Supplementary table 2 Alcohol use disorder in the female population. Total number (N) with indication, percent % and number per cell (n) with register indication by age-group, migrant status, education, marital status, phi - the correlation coefficient between the registers and the unique contribution of the specific register to all registers combined (Any registers) for the following registers: Inpatient care, Outpatient care, the Register of Medications (Medications), the Social Insurance register (Social Insurance), the Register of convictions (Convictions), and Any register.

		**Inpatient**	**Outpatient**	**Medication**	**Social Insurance**	**Convictions**	**Any registers**
		n=20051	n=30834	n=31519	n=2908	n=195	n=52389
**Age**	**20–29**	0.72 (2290)	1.28 (4073)	0.74 (2361)	0.04 (126)	0.01 (20)	1.99 (6317)
	**30–39**	0.53 (3020)	0.91 (5213)	0.86 (4899)	0.08 (447)	0.01 (44)	1.49 (8562)
	**40–49**	0.84 (5253)	1.30 (8173)	1.42 (8917)	0.16 (1017)	0.01 (64)	2.15 (13461)
	**50–59**	0.95 (5437)	1.39 (7984)	1.60 (9215)	0.17 (999)	0.01 (46)	2.42 (13885)
	**60–65**	0.68 (4051)	0.90 (5391)	1.03 (6127)	0.05 (319)	0.00 (21)	1.71 (10164)
**Migration status**	**Born in Sweden**	0.76 (16946)	1.17 (26229)	1.23 (27502)	0.12 (2581)	0.01 (182)	2.01 (44962)
	**Born abroad**	0.73 (3024)	1.08 (4458)	0.95 (3927)	0.08 (324)	0.00 (13)	1.74 (7204)
	**Refugee**	0.24 (81)	0.43 (147)	0.26 (90)	0.01 (3)	0.00 (0)	0.65 (223)
**Education**	**Education Pre-upper**	1.44 (5505)	1.94 (7409)	1.78 (6792)	0.13 (506)	0.02 (69)	3.24 (12375)
	**Upper second**	0.85 (10640)	1.28 (16093)	1.31 (16417)	0.13 (1692)	0.01 (84)	2.18 (27397)
	**Post-second**	0.37 (3697)	0.70 (7053)	0.80 (8096)	0.07 (702)	0.00 (40)	1.21 (12164)
	**Miss**	0.49 (209)	0.66 (279)	0.50 (214)	0.02 (8)	0.00 (2)	1.07 (453)
**Marital status**	**Married**	0.42 (5082)	0.66 (8031)	0.80 (9748)	0.07 (821)	0.00 (45)	1.22 (14853)
	**Unmarried**	0.82 (8644)	1.35 (14143)	1.20 (12556)	0.12 (1214)	0.01 (102)	2.17 (22792)
	**Divorced**	1.58 (5707)	2.18 (7911)	2.31 (8369)	0.23 (815)	0.01 (43)	3.69 (13352)
	**Widow(er)**	1.16 (618)	1.41 (749)	1.59 (846)	0.11 (58)	0.01 (5)	2.62 (1392)
**Unique contribution to Any register**	**%**	9.60 (5029)	20.09 (10527)	26.16 (13705)	0.53 (277)	0.19 (98)	-

Lundin A, *et al. BMJ Open* 2023; 13:e070744. doi: 10.1136/bmjopen-2022-070744

Supplementary table 3 The association between socioeconomic factors and appearing in specific registers among men with an indication of alcohol use disorder (n= 114 789) by the following registers: the Inpatient care, the Outpatient care, the Register of Medications (Medications), the Social Insurance register (Social Insurance) and the Register of convictions (Convictions), presented with odds ratios (OR) with 95 % confidence interval (CI), by age group, sex, migrant status, education level and marital status all included in the multivariate models.

		**Inpatient**	**Outpatient**	**Medication**	**Social Insurance**	**Convictions**
**Age-group**	**20–29**	0.55 (0.53-0.58)	0.98 (0.94-1.03)	0.54 (0.52-0.57)	0.33 (0.29-0.38)	1.78 (1.49-2.11)
	**30–39**	0.60 (0.58-0.63)	0.98 (0.95-1.02)	0.94 (0.91-0.98)	0.69 (0.64-0.75)	1.55 (1.33-1.81)
	**40–49**	0.82 (0.80-0.85)	1.04 (1.01-1.08)	1.17 (1.13-1.21)	1.00 (0.94-1.07)	1.32 (1.15-1.52)
	**50–59**	1	1	1	1	1
	**60–64**	1.12 (1.08-1.15)	0.89 (0.86-0.92)	0.73 (0.71-0.76)	0.43 (0.4-0.47)	0.64 (0.54-0.76)
**Migrant status**	**Migrant**	1.15 (1.11-1.19)	1.35 (1.30-1.40)	0.60 (0.57-0.62)	0.78 (0.72-0.85)	1.4 (1.22-1.6)
	**Refugee**	0.93 (0.82-1.06)	1.24 (1.10-1.41)	0.37 (0.33-0.42)	0.51 (0.36-0.71)	1.39 (0.9-2.13)
	**Sweden**	1	1	1	1	1
**Education**	**Pre-upper secondary school**	1.14 (1.11-1.17)	1.03 (1.01-1.06)	0.92 (0.89-0.94)	0.84 (0.79-0.89)	1.55 (1.39-1.72)
	**Upper secondary**	1	1	1	1	1
	**Post-secondary school**	0.69 (0.67-0.72)	1.10 (1.06-1.13)	1.19 (1.15-1.24)	0.83 (0.77-0.9)	0.51(0.42-0.62)
	**Missing**	1.26 (1.12-1.42)	0.97 (0.86-1.09)	0.76 (0.68-0.86)	0.4 (0.26-0.6)	1.75 (1.25-2.46)
**Marital status**	**Married**	1	1	1	1	1
	**Single**	1.74 (1.69-1.80)	1.45 (1.41-1.50)	0.57 (0.55-0.59)	1.13 (1.06-1.21)	1.3 (1.12-1.5)
	**Divorced**	1.77 (1.71-1.84)	1.47 (1.42-1.52)	0.65 (0.63-0.68)	1.33 (1.24-1.44)	1.64 (1.4-1.92)
	**Widow/Widower**	1.36 (1.21-1.54)	1.33 (1.17-1.50)	0.72 (0.63-0.82)	1.19 (0.89-1.6)	1.72 (1-2.96)

Lundin A, *et al. BMJ Open* 2023; 13:e070744. doi: 10.1136/bmjopen-2022-070744

Supplementary table 4 The association between socioeconomic factors and appearing in specific registers among women with an indication of alcohol use disorder (n= 52 389) by the following registers: the Inpatient care, the Outpatient care, the Register of Medications (Medications), the Social Insurance register (Social Insurance) and the Register of convictions (Convictions), presented with odds ratios (OR) with 95 % confidence interval (CI), by age group, sex, migrant status, education level and marital status all included in the multivariate models.

		**Inpatient**	**Outpatient**	**Medication**	**Social Insurance**	**Convictions**
**Age-group**	**20–29**	0.81 (0.76-0.87)	1.24 (1.16-1.33)	0.33 (0.31-0.35)	0.26 (0.22-0.32)	0.74 (0.42-1.3)
	**30–39**	0.85 (0.80-0.90)	1.11 (1.05-1.18)	0.70 (0.66-0.74)	0.71 (0.63-0.8)	1.39 (0.9-2.15)
	**40–49**	1.00 (0.95-1.05)	1.13 (1.08-1.19)	1.00 (0.95-1.06)	1.04 (0.95-1.14)	1.4 (0.95-2.06)
	**50–59**	1	1	1	1	1
	**60–64**	1.03 (0.98-1.09)	0.86 (0.82-0.91)	0.75 (0.71-0.79)	0.43 (0.38-0.49)	0.6 (0.36-1.02)
**Migrant status**	**Migrant**	1.16 (1.10-1.22)	1.19 (1.13-1.26)	0.71(0.67-0.75)	0.76 (0.68-0.86)	0.44 (0.25-0.77)
	**Refugee**	0.97 (0.73-1.27)	1.30 (0.99-1.72)	0.48 (0.36-0.63)	0.24 (0.08-0.75)	-
	**Sweden**	1	1	1	1	1
**Education**	**Pre-upper secondary school**	1.26 (1.21-1.31)	1.04 (1.00-1.09)	0.87 (0.84-0.91)	0.7 (0.63-0.78)	1.93 (1.4-2.66)
	**Upper secondary**	1	1	1	1	1
	**Post-secondary school**	0.69 (0.66-0.72)	1.01 (0.97-1.05)	1.27 (1.21-1.33)	0.93 (0.85-1.02)	1.11(0.76-1.63)
	**Missing**	1.40 (1.16-1.69)	0.98 (0.81-1.19)	0.95 (0.78-1.15)	0.42 (0.21-0.85)	2.1 (0.51-8.7)
**Marital status**	**Married**	1	1	1	1	1
	**Single**	1.23 (1.17-1.29)	1.27 (1.21-1.33)	0.83 (0.79-0.87)	1.09 (0.99-1.2)	1.29 (0.89-1.88)
	**Divorced**	1.37 (1.31-1.44)	1.24 (1.18-1.30)	0.87 (0.83-0.92)	1.1 (1-1.22)	1.06 (0.7-1.62)
	**Widow/Widower**	1.42 (1.27-1.59)	1.08 (0.96-1.20)	0.87 (0.78-0.98)	0.95 (0.72-1.26)	1.48 (0.58-3.8)

Lundin A, *et al. BMJ Open* 2023; 13:e070744. doi: 10.1136/bmjopen-2022-070744

Supplementary table 5 Drug use disorder in the male population. Total number (N) with indication, percent % and number per cell (n) with register indication by age-group, migrant status, education, marital status, phi - the correlation coefficient between the registers and the unique contribution of the specific register to all registers combined (Any registers) for the following registers: Inpatient care, Outpatient care, the Register of Medications (Medications), the Social Insurance register (Social Insurance), the Register of convictions (Convictions), and Any register.

	**Inpatient**	**Outpatient**	**Medication**	**Social Insurance**	**Convictions**	**Any registers**
	n= 20365	n= 29671	n= 8117	n= 2449	n= 48155	n= 69928
**20–29**	1.60 (5314)	2.33 (7740)	0.26 (875)	0.23 (777)	5.30 (17643)	6.28 (20889)
**30–39**	0.98 (5808)	1.44 (8551)	0.33 (1979)	0.15 (877)	2.48 (14738)	3.25 (19323)
**40–49**	0.73 (4744)	1.07 (6964)	0.30 (1951)	0.08 (495)	1.43 (9331)	2.25 (14642)
**50–59**	0.55 (3258)	0.81 (4728)	0.31 (1793)	0.04 (264)	0.94 (5511)	1.81 (10620)
**60–65**	0.21 (1241)	0.28 (1688)	0.25 (1519)	0.01 (36)	0.16 (932)	0.74 (4454)
**Born in Sweden**	0.72 (16895)	1.03 (24099)	0.28 (6545)	0.09 (2115)	1.56 (36426)	2.33 (54457)
**Born abroad**	0.77 (2908)	1.22 (4574)	0.32 (1210)	0.08 (286)	2.66 (9978)	3.48 (13084)
**Refugee**	1.00 (562)	1.78 (998)	0.65 (362)	0.09 (48)	3.13 (1751)	4.27 (2387)
**Education Pre-upper**	1.74 (8767)	2.42 (12204)	0.64 (3231)	0.16 (819)	3.97 (20080)	5.31 (26807)
**Upper second**	0.70 (9593)	1.04 (14240)	0.28 (3842)	0.10 (1364)	1.76 (24123)	2.60 (35597)
**Post-second**	0.19 (1605)	0.32 (2671)	0.11 (954)	0.03 (246)	0.32 (2725)	0.71 (5994)
**Miss**	0.81 (400)	1.13 (556)	0.18 (90)	0.04 (20)	2.50 (1227)	3.11 (1530)
**Married**	0.20 (2321)	0.31 (3558)	0.19 (2176)	0.02 (271)	0.36 (4067)	0.79 (9019)
**Unmarried**	1.15 (15198)	1.67 (22078)	0.35 (4569)	0.15 (1921)	2.95 (3891)	3.93 (51823)
**Divorsed**	0.95 (2771)	1.35 (3927)	0.45 (1307)	0.09 (251)	1.75 (5100)	3.05 (8861)
**Widow(er)**	0.44 (75)	0.63 (108	0.38 (65)	0.04 (6)	0.44 (76)	1.32 (225)
**Unique contribution to Any register**	5.35 (3744)	11.92 (8332)	4.48 (3131)	0.39 (273)	44.08 (30825)	

Supplementary table 6 Drug use disorder in the female population. Total number (N) with indication, percent % and number per cell (n) with register indication by age-group, migrant status, education, marital status, phi - the correlation coefficient between the registers and the unique contribution of the specific register to all registers combined (Any registers) for the following registers: Inpatient care, Outpatient care, the Register of Medications (Medications), the Social Insurance register (Social Insurance), the Register of convictions (Convictions), and Any register.

	**Inpatient**	**Outpatient**	**Medication**	**Social Insurance**	**Convictions**	**Any registers**
	n= 11810	n= 16452	n= 5176	n= 1110	n= 9056	n= 29624
**20–29**	0.81 (2565)	1.06 (3363)	0.15 (479)	0.08 (255)	0.99 (3145)	1.92 (6095)
**30–39**	0.50 (2861)	0.70 (4020)	0.16 (912)	0.06 (338)	0.43 (2459)	1.17 (6703)
**40–49**	0.48 (3022)	0.68 (4237)	0.19 (1203)	0.05 (319)	0.35 (2196)	1.18 (7418)
**50–59**	0.36 (2084)	0.54 (3083)	0.22 (1281)	0.03 (158)	0.19 (1115)	0.99 (5659)
**60–65**	0.21 (1278)	0.29 (1749)	0.22 (1301)	0.01 (40)	0.02 (141)	0.63 (3749)
**Born in Sweden**	0.45 (10002)	0.62 (13989)	0.20 (4539)	0.04 (993)	0.35 (7910)	1.12 (25177)
**Born abroad**	0.40 (1677)	0.55 (2292)	0.14 (595)	0.03 (110)	0.25 (1054)	1.00 (4125)
**Refugee**	0.38 (131)	0.50 (171)	0.12 (42)	0.02 (7)	0.27 (92)	0.94 (322)
**Education Pre-upper**	1.07 (4100)	1.44 (5498)	0.42 (1621)	0.07 (255)	1.09 (4170)	2.55 (9750)
**Upper second**	0.45 (5621)	0.62 (7830)	0.20 (2458)	0.05 (579)	0.32 (4074)	1.15 (14422)
**Post-second**	0.19 (1870)	0.28 (2808)	0.10 (1035)	0.03 (270)	0.06 (618)	0.49 (4954)
**Miss**	0.52 (219)	0.74 (316)	0.15 (62)	0.01 (6)	0.46 (194)	1.17 (498)
**Married**	0.21 (2575)	0.29 (3601)	0.15 (1854)	0.02 (242)	0.08 (1000)	0.58 (7031)
**Unmarried**	0.60 (6312)	0.85 (8911)	0.19 (2034)	0.06 (677)	0.60 (6305)	1.48 (15566)
**Divorsed**	0.76 (2737)	1.02 (3680)	0.31 (1126)	0.05 (181)	0.46 (1662)	1.80 (6533)
**Widow(er)**	0.35 (186)	0.49 (260)	0.30 (162)	0.02 (10)	0.17 (89)	0.93 (494)
**Unique contribution to Any register**	14.20 (4204)	25.60 (7583)	10.64 (3152)	0.79 (233)	16.53 (4898)	--

<u>Supplementary table 7</u> The association between socioeconomic factors and appearing in specific registers among men with an indication of drug use disorder (n= 69 928) by the following registers: the Inpatient care, the Outpatient care, the Register of Medications (Medications), the Social Insurance register (Social Insurance) and the Register of convictions (Convictions), presented with odds ratios (OR) with 95 % confidence interval (CI), by age group, sex, migrant status, education level and marital status all included in the multivariate models.

		**Inpatient**	**Outpatient**	**Medication**	**Social Insurance**	**Convictions**
		OR (95 % CI)	OR (95 % CI)	OR (95 % CI)	OR (95 % CI)	OR (95 % CI)
**Age-group**	**20–29**	0.73 (0.69-0.77)	0.70 (0.67-0.74)	0.24 (0.22-0.27)	1.77 (1.52-2.07)	4.07 (3.84-4.31)
	**30–39**	0.96 (0.91-1.02)	0.98 (0.93-1.03)	0.60 (0.55-0.64)	2.08 (1.80-2.41)	2.72 (2.58-2.87)
	**40–49**	1.10 (1.04-1.16)	1.14 (1.08-1.20)	0.77 (0.72-0.83)	1.44 (1.24-1.68)	1.57 (1.49-1.65)
	**50–59**	1	1	1	1	1
	**60–65**	0.86 (0.80-0.93)	0.75 (0.69-0.80)	2.22 (2.05-2.42)	0.29 (0.21-0.42)	0.28 (0.26-0.31)
**Migrant status**	**Born abroad**	0.62 (0.59-0.65)	0.67 (0.64-0.69)	0.73 (0.69-0.79)	0.54 (0.47-0.61)	1.69 (1.61-1.78)
	**Refugee**	0.67 (0.61-0.74)	0.88 (0.81-0.96)	1.27 (1.13-1.43)	0.49 (0.37-0.66)	1.60 (1.45-1.77)
	**Sweden**	1	1	1	1	1
**Education**	**Pre-upper secondary school**	1.39 (1.34-1.44)	1.32 (1.28-1.37)	1.28 (1.22-1.35)	0.79 (0.72-0.86)	1.30 (1.25-1.35)
	**Upper secondary**	1	1	1	1	1
	**Post-secondary school**	1.02 (0.96-1.09)	1.24 (1.17-1.31)	1.13 (1.04-1.22)	1.21 (1.05-1.39)	0.48 (0.45-0.51)
	**Missing**	1.33 (1.18-1.50)	1.16 (1.04-1.29)	0.77 (0.62-0.96)	0.40 (0.25-0.62)	1.21 (1.06-1.39)
**Marital status**	**Married**	1	1	1	1	1
	**Single**	1.18 (1.12-1.25)	1.17 (1.11-1.23)	0.50 (0.47-0.54)	0.86 (0.75-0.98)	2.16 (2.05-2.28)
	**Divorced**	1.24 (1.17-1.33)	1.17 (1.10-1.25)	0.48 (0.45-0.52)	1.01(0.85-1.21)	2.09 (1.96-2.23)
	**Widow/Widower**	1.34 (1.01-1.78)	1.40 (1.08-1.84)	0.83 (0.61-1.12)	1.32 (0.58-3.02)	1.17 (0.86-1.58)

Lundin A, *et al. BMJ Open* 2023; 13:e070744. doi: 10.1136/bmjopen-2022-070744

Supplementary table 8 The association between socioeconomic factors and appearing in specific registers among women with an indication of drug use disorder (n= 29 624) by the following registers: the Inpatient care, the Outpatient care, the Register of Medications (Medications), the Social Insurance register (Social Insurance) and the Register of convictions (Convictions), presented with odds ratios (OR) with 95 % confidence interval (CI), by age group, sex, migrant status, education level and marital status all included in the multivariate models.

		**Inpatient**	**Outpatient**	**Medication**	**Social Insurance**	**Convictions**
		OR (95 % CI)	OR (95 % CI)	OR (95 % CI)	OR (95 % CI)	OR (95 % CI)
**Age-group**	**20–29**	1.29 (1.19-1.41)	0.99 (0.91-1.08)	0.31 (0.27-0.35)	1.71 (1.37-2.14)	3.16 (2.88-3.47)
	**30–39**	1.33 (1.23-1.44)	1.24 (1.15-1.34)	0.54 (0.49-0.60)	1.88 (1.53-2.30)	2.16 (1.97-2.36)
	**40–49**	1.20 (1.11-1.29)	1.12 (1.04-1.20)	0.67 (0.61-0.73)	1.59 (1.31-1.94)	1.65 (1.52-1.80)
	**50–59**	1	1	1	1	1
	**60–65**	0.89 (0.81-0.97)	0.74 (0.68-0.81)	1.69 (1.54-1.86)	0.37 (0.26-0.53)	0.17 (0.14-0.20)
**Migrant status**	**Migrant**	1.02 (0.95-1.09)	0.98 (0.92-1.05)	0.73 (0.66-0.80)	0.69 (0.57-0.85)	0.79 (0.73-0.86)
	**Refugee**	0.99 (0.79-1.24)	0.88 (0.70-1.10)	0.78 (0.56-1.09)	0.52 (0.24-1.10)	0.77 (0.60-1.00)
	**Sweden**	1	1	1	1	1
**Education**	**Pre-upper secondary school**	1.11 (1.06-1.17)	1.08 (1.03-1.14)	1.09 (1.01-1.17)	0.62 (0.54-0.72)	1.79 (1.69-1.90)
	**Upper secondary**	1	1	1	1	1
	**Post-secondary school**	0.98 (0.92-1.05)	1.14 (1.07-1.22)	1.10 (1.01-1.20)	1.51 (1.30-1.75)	0.42 (0.38-0.47)
	**Missing**	1.17 (0.97-1.41)	1.49 (1.23-1.79)	1.12 (0.85-1.48)	0.29 (0.13-0.64)	1.27 (1.05-1.55)
**Marital status**	**Married**	1	1	1	1	1
	**Single**	1.04 (0.98-1.11)	1.19 (1.12-1.27)	0.66 (0.61-0.71)	1.01 (0.86-1.19)	2.25 (2.07-2.44)
	**Divorced**	1.27 (1.18-1.36)	1.25 (1.16-1.33)	0.55 (0.50-0.60)	0.88 (0.72-1.07)	2.19 (1.99-2.40)
	**Widow/Widower**	1.16 (0.96-1.40)	1.20 (1.00-1.45)	0.97 (0.80-1.19)	0.93 (0.49-1.77)	2.38 (1.83-3.10)

Lundin A, *et al. BMJ Open* 2023; 13:e070744. doi: 10.1136/bmjopen-2022-070744
