# Supplementary material for: Indications of alcohol or drug use disorders in five different national registers in Sweden: a cross-sectional population-based study
Source: BMJ Open. 2023 Sep 4;13(9):e070744. doi: 10.1136/bmjopen-2022-070744 (PMC10481836; doi:10.1136/bmjopen-2022-070744)
Supplement: Author's manuscript [file bmjopen-2022-070744.draft_revisions.pdf]

BMJ Open

BMJ Open is committed to open peer review. As part of this commitment we make the peer review history of every article we publish publicly available.

When an article is published we post the peer reviewers' comments and the authors' responses online. We also post the versions of the paper that were used during peer review. These are the versions that the peer review comments apply to.

The versions of the paper that follow are the versions that were submitted during the peer review process. They are not the versions of record or the final published versions. They should not be cited or distributed as the published version of this manuscript.

BMJ Open is an open access journal and the full, final, typeset and author-corrected version of record of the manuscript is available on our site with no access controls, subscription charges or pay-per-view fees (http://bmjopen.bmj.com).

If you have any questions on BMJ Open's open peer review process please email info.bmjopen@bmj.com

BMJ Open

Indications of alcohol or drug use disorders in five different national registers in Sweden – is there a correlation between the registers or are the separate registers identifying different populations. A cross sectional population-based study.

Journal:	*BMJ Open*
Manuscript ID	bmjopen-2022-070744
Article Type:	Original research
Date Submitted by the Author:	02-Dec-2022

Complete List of Authors:	Lundin, Andreas; Karolinska Institutet, Department of Global Public Health ; Centre for Epidemiology and Community Medicine, Region Stockholm<br>Danielsson, Anna-Karin; Karolinska Institutet, Global Public Health<br>Dalman, Christina; Karolinska Institute, Global Public Health ; Centre for Epidemiology and Community Medicine, Region Stockholm<br>Hollander, Anna-Clara; Karolinska Institutet, Department of Global Public Health

Keywords:	EPIDEMIOLOGY, Substance misuse < PSYCHIATRY, PUBLIC HEALTH, SOCIAL MEDICINE

SCHOLARONE™ Manuscripts

I, the Submitting Author has the right to grant and does grant on behalf of all authors of the Work (as defined in the below author licence), an exclusive licence and/or a non-exclusive licence for contributions from authors who are: i) UK Crown employees; ii) where BMJ has agreed a CC-BY licence shall apply, and/or iii) in accordance with the terms applicable for US Federal Government officers or employees acting as part of their official duties; on a worldwide, perpetual, irrevocable, royalty-free basis to BMJ Publishing Group Ltd ("BMJ") its licensees and where the relevant Journal is co-owned by BMJ to the co-owners of the Journal, to publish the Work in this journal and any other BMJ products and to exploit all rights, as set out in our licence.

The Submitting Author accepts and understands that any supply made under these terms is made by BMJ to the Submitting Author unless you are acting as an employee on behalf of your employer or a postgraduate student of an affiliated institution which is paying any applicable article publishing charge ("APC") for Open Access articles. Where the Submitting Author wishes to make the Work available on an Open Access basis (and intends to pay the relevant APC), the terms of reuse of such Open Access shall be governed by a Creative Commons licence – details of these licences and which Creative Commons licence will apply to this Work are set out in our licence referred to above.

Other than as permitted in any relevant BMJ Author's Self Archiving Policies, I confirm this Work has not been accepted for publication elsewhere, is not being considered for publication elsewhere and does not duplicate material already published. I confirm all authors consent to publication of this Work and authorise the granting of this licence.

For peer review only

Title page

The title: Indications of alcohol- or drug use disorders in five different national registers in Sweden – is there a correlation between the registers or do the separate registers identify different populations? A cross sectional population-based study.

List the full names and institutional addresses for all authors:

Andreas Lundin[1,2]

Anna-Karin Danielsson[1]

Christina Dalman[1,2]

Anna-Clara Hollander[1]*

[1]Epidemiology of Psychiatric Conditions, Substance Use and Social Environment (EPiCSS), Department of Global Public Health, KI, Solnavägen 1E, 171 77 Stockholm, Sweden

[2]Centre for Epidemiology and Community Medicine, Region Stockholm, Stockholm, Sweden

*the corresponding author

For peer review only

Abstract

Background

The purpose of this study is to examine the prevalence of indications of alcohol- or drug use disorders in five different national Swedish registers and to investigate the correlation between these registers. Furthermore, the study seeks to determine if combining data from different registers increases the prevalence of these indications in the population, due to registers identifying different demographic groups.

Methods

We included all individuals living in Sweden who were aged 20–64 in 2006. The combined national registers were registers of in- and outpatient care; of prescribed and purchased medication; of sickness and of activity compensation; and the register of convicted of crime. Demographic variables were gender, age, migrant status, education, and civil status. Indications of alcohol or drug use disorders were presented as prevalence in percentage (%). Correlation was examined with a phi-correlation coefficients and differences across demographic factors using logistic regression, presented as odds ratios (OR) and 95 % confidence intervals (CI).

Results

The prevalence of an indication of alcohol- or drug use disorder varied between registers, meaning that the prevalence increased when all registers were considered together. The prevalence of alcohol use disorder increased by 60 % and 66% amongst men and women, respectively, while the prevalence of drug use disorder increased by 45 % and 80% amongst men and women, respectively, when all registers were combined compared to solely using the register with the highest prevalence. Combined registers detected more cases among young people, migrants, and individuals with a higher education.

Conclusions

Having accurate estimates of alcohol or drug use disorders is critical for prevention, health care and rehabilitation. This study shows that using a single register alone underestimates the burden of disease unevenly, while combining a range of registers can provide a more accurate picture.

Keywords

alcohol use disorder; drug use disorder; substance use disorder; population based; nationwide; Sweden

Word count: 4632

For peer review only

Strengths and limitations of this study

This section should be no more than 5 bullet points relating specifically to the methods - not the results of the study. This will be published as a summary box after the abstract in the final published article.

- Five different national Swedish registers were used to investigate the correlation between the registers to find out if combining the registers increase the prevalence of indications of alcohol- or drug use disorders in the population, due to registers identifying different demographic groups.
- The study included individuals living in Sweden aged 20–64 in 2006.
- The combined national registers were: the registers of inpatient and outpatient care; the register of prescribed and purchased medication; the registers of sickness and activity compensation; and the register of convicted of crime.
- This study examines the proportion with an indication of alcohol- or drug use disorder during a certain period (period prevalence) and this indication does not take previous use into account.
- The validity of indications of alcohol- and drug use disorder in this study depends on the validity of the different registers.

For peer review only

Background

Alcohol and drug use cause a substantial disease burden globally, the composition and extent of which varies between countries [1]. Globally, in 2016, 4.2% of all DALYs were attributable to alcohol use, and 1.3% to drug use [1]. The lost DALYs were due to the risk of injury, premature death, and other negative consequences [2]. However, the risk posed by using alcohol and drugs differs between men and women, by socioeconomic conditions, and for migrants by country of birth [3-5].

Substance use disorder combines the categories of substance abuse and substance dependence into a single disorder. Health care planning in a demographically changing society requires updated analyses of substance use disorder. Although studies of the negative consequences of alcohol and drug use in a population commonly use surveys, registry-based studies provide an additional way of studying the harms of alcohol and drug use [6-10]. There are many advantages to using survey data in studies of alcohol and drug use in a population, such as the ability to study different degrees of the disorder as the participants describe their consumption and their related problems themselves. However, disadvantages include the decreasing response rate in population surveys in general [11], and that heavy substance use can affect memory [12], increasing the risk of recall bias.

In Scandinavia and Finland, health care visits are recorded in local and national administrative registers covering the entire population. Such population-based registers have benefited psychiatric research immensely [13]. These health registers are generally considered valid [19], but using registers collected for administrative purposes in research can give misleading results if there is a systematic underutilisation of care in specific sub-groups. In Sweden to date, studies of substance use disorder have used the National Patient Register [6, 10, 20] from the perspective that the most severe cases of substance use disorder will require care. This register has been an important source of knowledge about the social determinants and consequences of severe alcohol- and drug use disorder. However, it has long been recognised that, in addition to detecting the most severe cases only, these registers possibly underestimate severe cases in specific sub-groups where under-utilisation is pronounced [21]. As register-based research could be a powerful tool for population-based studies of substance use disorders, there have been attempts to expand the use of population-registers by including additional registers including less severe cases, such as registers with diagnoses from GP surgeries [22], the Crime Prevention Council's register [8], and the Register of prescribed and purchased medicine [9]. Studies using the additional registers recognise that appearing in the additional registers is not the same as being diagnosed with a substance use disorder but acknowledge that it is a possible indication of a substance use disorder. However, despite being used in studies of the determinants and consequences of a substance use disorder, to date this practice has not been tested empirically. The purpose of this study is to examine the prevalence of indications of alcohol- or drug use disorders in five different national Swedish registers. This is in order to investigate the extent of correlation between the registers to find out if combining the registers increases the prevalence of such indications in the population, due to registers identifying different demographic groups such as different age-groups, sexes, educational level, migrant or marital status.

Methods

Design

We conducted a historical cross-sectional register-based study in several Swedish national registries, using the personal identity numbers that are uniquely assigned to every resident at birth or immigration to link registers.

Data sources

For peer review only - http://bmjopen.bmj.com/site/about/guidelines.xhtml

Data were extracted from a linked register database called Psychiatry Sweden (PS) held by the research group Epidemiology of Psychiatric Conditions, Substance use and Social environment (EPICSS). PS consists of several national and regional registers.

For the registers of alcohol and drug use disorders we used:

From the National Board of Health and Welfare we used:

- the Patient register [20] that contains two registers; one for inpatient care (henceforth called the Inpatient register) and another for specialized outpatient care (henceforth called the Outpatient register)
- the Register of prescribed and purchased pharmaceuticals (Läkemedelsregistret)[25], a register of all medicines dispensed at pharmacies (henceforth called the Register of Medications).

We also included the Swedish Social Insurance Agency's (Försäkringskassan) [26] registers of sickness or activity compensation included in Mikrodata for analysis of social insurance (henceforth called the Social Insurance register).

From the Crime Prevention Council's register of convicted of crime (Brottsförebyggande rådets (BRÅs)) Register över lagförda personer [27] we used the Register of prosecuted convictions based on the information registered at the Swedish Public Prosecutor's Office and courts (henceforth called the Register of convictions).

In addition, we used registers from Statistics Sweden for the demographic factors:

- the Register of the total population
- the Longitudinal integration database for health insurance and labour market studies (LISA) [23]
- the Longitudinal database for integration studies STATIV [24].

<u>Population</u>

The population consisted of all persons in Sweden aged 20–64 during 2006 and was identified in LISA. These individuals were then followed up in various registers regarding the indication of alcohol and drug use disorder between 2006 and 2016.

<u>Definitions of indication of alcohol or drug use disorder</u>

The present study focused on use that was so extensive and problematic that it was captured in national registers and will henceforth be called a register indication of alcohol or drug use disorder. The registers primarily use the International Statistical Classification of Diseases and Related Health Problems, 10th Edition (ICD-10) [28] system of classification where alcohol and drug use disorder has six possible criteria:

- Craving
- Loss of control
- Prioritization of alcohol consumption over other activities
- Continued intake despite adverse effects
- Increased tolerance
- Abstinence

For peer review only - http://bmjopen.bmj.com/site/about/guidelines.xhtml

For a person to be diagnosed according to ICD-10, three of six criteria must be met during the past year [28].

Alcohol use disorder

In both the Patient registers and the Social Insurance register, an indication of alcohol dependence was an ICD-10 diagnosis of "Mental disorders and behavioural disorders caused by alcohol (F10)".

In the register of convictions, an indication of alcohol use disorder was defined as relevant alcohol-related crimes such as drunk driving, aggravated drunk driving or care of intoxicated persons (Act (1951: 649) on penalties for certain traffic offenses, § 4 drunk driving and § 4a aggravated drunk driving respectively). Since one of the ICD-10 criteria for dependence is still drinking despite negative effects, it was only counted as a register indication if an individual had more than two convictions during the same calendar year.

In the Register of Medications, an indication of alcohol dependence was defined as having been prescribed medicine against alcohol use disorder including Disulfiram (ATC NO7BB01), Acamprosate (ATC NO7BB03), Naltrexone (ATC NO7BB04) and Nalmefene (ACT NO7BB05).

Drug use disorder

In the Patient registers and the Social Insurance register, an indication of drug use disorder was an ICD-10 diagnosis of chapters F11 – F19, which are “Mental disorders and behavioural disorders caused by opioids, cannabis, sedatives and hypnotics, cocaine, other stimulants, hallucinogens and volatile solvents and of other psychoactive substances".

In the Register of convictions, an indication of drug use disorder was relevant drug-related crime (Narcotics Penal Code (1968: 64) and the Act (1991: 1969) on the prohibition of certain substances such as possession, use of, or other engagement with drugs. As one of the ICD-10 criteria for dependence is continued use despite negative effects, it was only counted as a register indication when a person had more than two convictions during the same calendar year.

In the Register of Medications, an indication of drug dependence was having been prescribed and dispensed Buprenorphine N07BC01, Methadone N07BC02, Levomethadone N07BC05 and Buprenorphine, combinations N07BC51 (only drugs for opiate dependence were available).

Co-variates

- Sex was defined as sex assigned at birth: male or female.
- Age was categorised in age groups: 20–29, 30–39, 40–49, 50–59 and 60–65.
- Migration status was grouped into those born in Sweden, migrants, and refugees; the refugees were defined as having received a residence permit for refugee reasons according to the Swedish Migration Agency.
- Education level was grouped according to Swedish education nomenclature as pre-secondary, upper secondary, post-secondary, and unknown.
- Marital status was grouped as married (including registered partnership), divorced (including deregistered partnership), widow / widower and unmarried.

Statistical analysis

Alcohol and drug use disorder in the registers were presented as cumulative period prevalence and reported in percentage. Correlation, i.e. the extent to which those with an indication of use disorder in one register appear in other registers, was examined with phi-correlation for dichotomous

For peer review only - http://bmjopen.bmj.com/site/about/guidelines.xhtml

variables. Phi correlation is interpreted as a scale where the correlation coefficient is between 1 and -1, where 1 means maximum positive correlation, 0 is no correlation at all, and -1 is maximum negative correlation. Relationships between the demographic variables (age, sex, migrant status, level of education, marital status) and indication of alcohol or drug use disorder in different registers were examined using logistic regression and presented as odds ratios (OR) and 95 percent confidence interval (CI). All regression models were adjusted for age, sex, migrant status, education level and marital status.

Results

The population consisted of all individuals in Sweden aged 20–64 in 2006, a total of 5.45 million individuals, and is described in Table 1.

Table 1. Characterise of the population in 2006 in percent (%).

**Characteristics**		(N = 5 453 616)
**Sex**	Male	50.7
	Female	49.3
**Migration status**	Born in Sweden	83.9
	Born abroad	14.5
	Refugee*	1.7
**Education**	Pre-upper secondary school	16.3
	Upper secondary	48.1
	Post-secondary school	33.9
	Missing	1.7
**Age group**	20–29	20.0
	30–39	22.6
	40–49	22.7
	50–59	21.9
	60–65	12.8
**Marital status**	Married	43.3
	Unmarried	43.4
	Divorced	12.0
	Widow widower	1.3

Table 2 shows the prevalence of an indication of alcohol use disorder, by sex and age in the population. In total, 2.0 % of the women and 4.2 % of the men appeared in one of these registers. Those aged 50–59 years had the highest prevalence in all registers except the Register of convictions. The prevalence of individuals with a register indication for alcohol use disorder differed between registers. Men were consistently found to have a higher prevalence than women. The highest prevalence for both men and women were found in the Register of Medications (men 2.6%, women 1.2%), followed by the Inpatient and Outpatient register and the Social Insurance register. The lowest prevalence was found in the Register of convictions. Comparing all registers combined with the register with the highest prevalence increased total prevalence by 60 % for men and 66 % for women. Prevalence in the different age groups increased correspondingly from 24 % to 67 %, with the largest increase among people aged 30–39 years. Furthermore, Table 2 shows the correlation i.e. the extent to which persons with an indication of alcohol use disorder in one register appear in other registers. Register indication in the Inpatient and Outpatient register and the Register of medication had the highest correlation. The correlation between having an indication in another register were greatest if a person appeared in the Register of medication and lowest if the person appeared in The Register of convictions.

For peer review only - http://bmjopen.bmj.com/site/about/guidelines.xhtml

Table 2. Register indication in percent (%) of alcohol use disorder in the population by sex or age-group during the period 2006–2016 in the following registers: Inpatient care, Outpatient care, the Register of Medications (Medication), the Social Insurance register (Social Insurance), the Register of convictions (Convictions), and all registers combined (any registers), and the Correlation (phi-the correlation coefficient) between the registers and any register.

	Inpatient	Outpatient	Medication	Social Insurance	Convictions	Any registers
**Male**	1.69	2.37	2.59	0.23	0.06	4.15
**Women**	0.75	1.15	1.17	0.11	0.01	1.95
**20–29**	0.91	1.58	1.10	0.06	0.04	2.59
**30–39**	0.78	1.33	1.35	0.12	0.03	2.25
**40–49**	1.27	1.91	2.17	0.24	0.04	3.22
**50–59**	1.66	2.25	2.57	0.29	0.04	3.91
**60–65**	1.37	1.67	1.89	0.10	0.02	3.13
**Inpatient**	1	0.54	0.41	0.25	0.04	0.63
**Outpatient**		1	0.49	0.22	0.04	0.75
**Medication**			1	0.22	0.04	0.78
**Sick leave**				1	0.02	0.23
**Convictions**					1	0.10
**Any registers**						1

For peer review only

Individuals with an indication of alcohol use disorder were n=167175 and differed by age, sex, migrant status, education level and marital status (see Table 3). Those with an indication of alcohol use disorder aged 20–29 or 40–49 years were more likely to be found in the Outpatient register compared to those in other age-groups, whereas those aged between 20-29 appeared more often in the Register of convictions. People aged 40–49 and 50–59 years appeared more often in the Register of Medications (Table 3). Women with an indications of alcohol use disorder appeared more often in the Outpatient register compared with men but had a lower likelihood of being found in the Register of convictions. The differences between the sexes in the Inpatient register and in the Social Insurance register were small (see Table 3). Migrants with an indications of alcohol use disorder were more likely to appear in the Inpatient and Outpatient register, and they were also more likely to have an indication in the Register of convictions, compared with those born in Sweden. Persons born in Sweden with an indication of alcohol use disorder, on the other hand, were more likely to appear in the Register of Medications and in the Social Insurance register compared with migrants (Table 3). People with an indication of alcohol use disorder and with post-secondary education appeared more often in the Register of Medications and the Outpatient register compared with other education levels. However, among those with an indication of alcohol use disorder and pre-secondary education only, it was more common to appear in the Register of convictions and in the Inpatient register. People with an indication of alcohol use disorder who were married were more likely to be found in in the Register of Medications than individuals who were unmarried, divorced, and widows and widowers (see Table 3).

For peer review only - http://bmjopen.bmj.com/site/about/guidelines.xhtml

Table 3. The association between socioeconomic factors and appearing in specific registers among those with an indication of alcohol use disorder (n=167175) by the following registers: the Inpatient care, the Outpatient care, the Register of Medications (Medication), the Social Insurance register (Social Insurance) and the Register of convictions (Convictions), and all registers combined (any register) and the Correlation (phi-the correlation coefficient) between those registers. Presented with odds ratios (OR) with 95 % confidence interval (CI), adjusted for age group, sex, migrant status, education level and marital status.

		**Inpatient**	**Outpatient**	**Medication**	**Social Insurance**	**Convictions**
		OR (95 % CI)	OR (95 % CI)	OR (95 % CI)	OR (95 % CI)	OR (95 % CI)
**Age-group**	**20–29**	0.62 (0.60–0.65)	1.07 (1.03–1.11)	0.46 (0.45–0.48)	0.30 (0.27–0.34)	1.63 (1.38–1.93)
	**30–39**	0.67 (0.65–0.69)	1.02 (0.99–1.06)	0.86 (0.83–0.89)	0.70 (0.65–0.75)	1.53 (1.33–1.78)
	**40–49**	0.87 (0.85–0.90)	1.07 (1.04–1.10)	1.11 (1.08–1.15)	1.01 (0.96–1.07)	1.34 (1.17–1.52)
	**50–59**	1	1	1	1	1
	**60–65**	1.09 (1.06–1.13)	0.88 (0.86–0.91)	0.74 (0.72–0.76)	0.43 (0.40–0.46)	0.64 (0.54–0.75)
**Sex**	**Female**	0.96 (0.94–0.98)	1.08 (1.06–1.10)	0.88 (0.86–0.90)	0.99 (0.94–1.03)	0.26 (0.22–0.30)
	**Male**	1	1	1	1	1
**Migrant status**	**Migrant**	1.15 (1.12–1.19)	1.29 (1.25–1.33)	0.63 (0.61–0.65)	0.77 (0.72–0.83)	1.27 (1.12–1.45)
	**Refugee**	0.93 (0.83–1.04)	1.25 (1.12–1.40)	0.39 (0.35–0.44)	0.46 (0.34–0.64)	1.28 (0.84–1.97)
	**Sweden**	1	1	1	1	1
**Education**	**Pre-upper secondary school**	1.17 (1.15–1.20)	1.04 (1.01–1.06)	0.90 (0.88–0.92)	0.80 (0.76–0.84)	1.58 (1.43–1.75)
	**Upper secondary**	1	1	1	1	1
	**Post-secondary school**	0.69 (0.67–0.71)	1.06 (1.03–1.09)	1.23 (1.19–1.26)	0.87 (0.82–0.92)	0.59 (0.49–0.70)
	**Missing**	1.30 (1.17–1.43)	0.98 (0.88–1.08)	0.81 (0.73–0.89)	0.40 (0.28–0.58)	1.82 (1.31–2.52)
**Marital status**	**Single**	1.56 (1.52–1.60)	1.39 (1.35–1.43)	0.64 (0.62–0.66)	1.11 (1.05–1.18)	1.29 (1.13–1.48)
	**Married**	1	1	1	1	1
	**Divorced**	1.62 (1.58–1.67)	1.39 (1.35–1.42)	0.73 (0.71–0.75)	1.25 (1.18–1.32)	1.56 (1.34–1.81)
	**Widow/Widower**	1.41 (1.29–1.53)	1.18 (1.09–1.28)	0.79 (0.73–0.86)	1.06 (0.87–1.30)	1.69 (1.06–2.71)

For peer review only - http://bmjopen.bmj.com/site/about/guidelines.xhtml

The prevalence of individuals with an indication of drug use disorder in any of the registers was 1.1 % amongst women and 2.5 % amongst men, while the 20–29 age group had the highest prevalence in all registers except in the Register of medication (Table 4). The prevalence of an indication of drug use disorder was consistently higher for men than women. For all registers, except in the Register of Medications, prevalence decreased with age. For men, the highest prevalence was found in the Register of convictions (1.7%), followed by the Outpatient and Inpatient register, and in the Register of Medications. For women, the highest prevalence was found in the Outpatient register (0.6%), followed by the Inpatient register, the Register of convictions and in the Register of Medications. Both men and women had the lowest prevalence in the Social Insurance register. For drug use disorder, the prevalence increased by 45 % for men and 80 % for women if all registers were combined, instead of the register with the highest prevalence alone. The prevalence in different age groups increased correspondingly by between 30 to 109 %, with the largest increase among people in the 50–59 age group. The was a correlation between having an indication of a drug use disorder between the registers. The correlation was highest between the Inpatient and Outpatient register (0.56). The correlation between having an indication in another register was greatest if the individual appeared in the Register of convictions (0.76) and lowest if they appeared in the Social Insurance register (0.19).

Table 4. Register indication in percent (%) of drug use disorder in the population by sex or age group during the period 2006–2016 in the following registers: the Inpatient care, the Outpatient care, the Register of Medications (Medication), the Social Insurance register (Social Insurance) and the Register of convictions (Convictions) and all registers combined (any register) and the Correlation (phi-the correlation coefficient) between the registers and any register.

	**Inpatient**	**Outpatient**	**Medication**	**Social Insurance**	**Convictions**	**Any registers**
**Male**	0.74	1.07	0.29	0.09	1.74	2.53
**Women**	0.44	0.61	0.19	0.04	0.34	1.10
**20–29**	1.21	1.71	0.21	0.16	3.20	4.15
**30–39**	0.74	1.08	0.25	0.10	1.47	2.23
**40–49**	0.61	0.88	0.25	0.06	0.90	1.73
**50–59**	0.46	0.67	0.26	0.04	0.57	1.40
**60–65**	0.21	0.29	0.24	0.01	0.09	0.69
**Inpatient**	1	0.56	0.23	0.20	0.33	0.56
**Outpatient**		1	0.27	0.21	0.36	0.68
**Medication**			1	0.10	0.15	0.36
**Sick leave**				1	0.12	0.19
**Convictions**					1	0.76
**Any registers**						1

Individuals with who were found to have an indication of drug use disorder (n=99529) differed by age, sex, migrant status, education level and marital status (see Table 5). Those aged 20–29 years and with an indication of drug use disorder were more likely to appear in the Register of convictions. Persons with an indication of drug use disorder aged 30–49 were more likely than other groups to be found both in the Register of convictions (together with those aged 20-29) and to have an indication in the Social Insurance register. The oldest age group (60–65 years) with an indication of drug use disorder were more likely to appear in the Register of Medications (see Table 5). In terms of sex

For peer review only - http://bmjopen.bmj.com/site/about/guidelines.xhtml

differences among those with an indication of drug use disorder, women were more likely to appear in the Outpatient and Inpatient registers as well as the Register of Medications compared with men, while men were more likely to appear in the Register of convictions. Migrants with an indication of drug use disorder were more likely to appear in the Register of convictions compared with individuals born in Sweden (see Table 5). Those with a pre-secondary education were more likely to appear in all registers, except the Social Insurance register, compared to those with a higher education. Unmarried and divorced individuals with an indication of drug use disorder were more likely to appear in the Inpatient register and in the Register of convictions compared with married individuals. Married individuals with an indication of drug use disorders appeared more often than other marital statuses in the Register of Medications.

For peer review only

For peer review only - http://bmjopen.bmj.com/site/about/guidelines.xhtml

Table 5. The association between socioeconomic factors and appearing in specific registers among those with an indication of drug use disorder n=99529 by the following registers: the Inpatient care, the Outpatient care, the Register of Medications (Medication), the Social Insurance register (Social Insurance) and the Register of convictions (Convictions) and any register that are all registers combined and the Correlation (phi-the correlation coefficient) between those registers. Presented with odds ratios (OR) with 95 % confidence interval (CI), adjusted for age group, sex, migrant status, education level and marital status.

		**Inpatient**	**Outpatient**	**Medication**	**Social Insurance**	**Convictions**
		OR (95 % CI)	OR (95 % CI)	OR (95 % CI)	OR (95 % CI)	OR (95 % CI)
**Age-group**	**20–29**	0.86 (0.82–0.90)	0.77 (0.74–0.80)	0.26 (0.24–0.28)	1.74 (1.53–1.97)	3.82 (3.64–4.01)
	**30–39**	1.07 (1.02–1.12)	1.05 (1.01–1.09)	0.57 (0.54–0.61)	2.00 (1.78–2.26)	2.57 (2.46–2.69)
	**40–49**	1.13 (1.08–1.18)	1.13 (1.09–1.18)	0.73 (0.69–0.77)	1.49 (1.32–1.68)	1.61 (1.54–1.69)
	**50–59**	1	1	1	1	1
	**60–65**	0.86 (0.81–0.91)	0.74 (0.70–0.78)	1.97 (1.85–2.09)	0.33 (0.26–0.42)	0.25 (0.23–0.27)
**Sex**	**Female**	1.61 (1.56–1.66)	1.69 (1.64–1.74)	1.27 (1.22–1.32)	1.07 (1.00–1.16)	0.22 (0.22–0.23)
	**Male**	1	1	1	1	1
**Migrant status**	**Born abroad**	0.72 (0.69–0.75)	0.74 (0.71–0.76)	0.73 (0.69–0.77)	0.58 (0.52–0.64)	1.38 (1.32–1.43)
	**Refugee**	0.73 (0.67–0.80)	0.90 (0.83–0.97)	1.20 (1.08–1.35)	0.50 (0.38–0.65)	1.40 (1.28–1.54)
	**Sweden**	1	1	1	1	1
**Education**	**Pre-upper secondary school**	1.30 (1.26–1.34)	1.25 (1.22–1.29)	1.21 (1.16–1.27)	0.74 (0.69–0.80)	1.44 (1.39–1.49)
	**Upper secondary**	1	1	1	1	1
	**Post-secondary school**	1.01 (0.96–1.05)	1.20 (1.15–1.26)	1.13 (1.06–1.20)	1.36 (1.23–1.50)	0.46 (0.44–0.49)
	**Missing**	1.27 (1.15–1.41)	1.23 (1.12–1.34)	0.89 (0.75–1.05)	0.36 (0.25–0.54)	1.22 (1.09–1.36)
**Marital status**	**Single**	1.14 (1.09–1.19)	1.19 (1.14–1.24)	0.56 (0.53–0.59)	0.91 (0.82–1.01)	2.15 (2.05–2.25)
	**Married**	1	1	1	1	1
	**Divorced**	1.25 (1.19–1.31)	1.20 (1.15–1.26)	0.51 (0.48–0.54)	0.95 (0.83–1.08)	2.12 (2.01–2.23)
	**Widow/ Widower**	1.17 (1.00–1.37)	1.21 (1.04–1.41)	0.87 (0.74–1.03)	1.05 (0.63–1.74)	1.72 (1.41–2.10)

For peer review only - http://bmjopen.bmj.com/site/about/guidelines.xhtml

Discussion

The aim of this study was first to explore the prevalence and correlation of indications of alcohol or drug use disorder in a range of national registers, to see if linking registers increased the prevalence of individuals with an indication of substance use disorders. This study demonstrates that the proportion of individuals in the population aged 20–64 years with an indication of alcohol or drug use disorder differs between registers but that there is a correlation between them. This means that those with a register indication of substance use in one register sometimes, but not always, appear in one or more other registers, hence the proportion increases when registers are added together. According to the combined registers, the proportion of women and men with an indication of alcohol use disorder were 2.0 % and 4.2 %, respectively, and with an indication of drug use disorder 1.1 % and 2.5 %, respectively. The proportion in the population with an indication of alcohol use disorder increased by more than 60 % for men and 66 % for women, with the largest increase among individuals aged 30–39 years (67%), in all registers combined when compared with only the register showing the highest prevalence. The proportion with an indication of drug use disorder in the population increased by 45 % for men and 80 % for women, with the largest increase among individuals aged 50–59 years (109%), in all registers combined when compared with only the register showing the highest prevalence.

An additional aim was to investigate if, amongst those with an indication of alcohol or drug use disorder, there was a demographic difference in the likelihood of appearing in a specific register. Individuals aged 20–29 years with an indication of either alcohol or drug use disorder had a lower likelihood of being found in the Inpatient register, but a higher likelihood of being found in the Register of convictions when compared with individuals aged 50–59 years. Compared with men, women with an indication of alcohol use disorder in any register had a similar likelihood to be found in the Outpatient register but a slightly lower likelihood of appearing in the Inpatient register and the Social Insurance register, and a much lower likelihood of appearing in the Register of convictions. Women with an indication of drug use disorder had, compared to men, a higher likelihood of being found in any of the registers, but a much lower likelihood of being found in the Register of convictions. Among those with an indication of alcohol use disorder, migrants, individuals with a pre-upper secondary school education, and unmarried individuals all had a lower likelihood of being registered in the Register of Medications and Social Insurance register. Migrants with an indication of drug use disorder had a lower likelihood of appearing in all registers except the Register of convictions, where the likelihood was higher compared to individuals born in Sweden. Individuals with a pre-upper secondary education and an indication of drug use disorder had a higher likelihood of being found in all registers except the register of Social Insurance when compared to individuals with an upper secondary education. Divorced individuals with an indication of drug use disorder had a higher or similar prevalence in all registers except the Register of Medications, where the likelihood was lower compared to married individuals.

Findings in context

In this study, using all registers showed the share of the population with an indication of alcohol use disorder as 4.2 % for men and 2.0 % for women. This can be compared to a cross-sectional study by Andréasson et al. based on survey-data from randomly selected 19 to 70-year-olds from 12 Swedish municipalities [3], where researchers found that the share classified as alcohol use disorder was 4.9 % for men and 3.2 % for women. The same study also found that an average of 4 % of the population in Sweden meet at least three criteria for alcohol use disorder, while among 19 to 25-year-olds the prevalence was higher (around 11 %) and in this group there was no statistical difference between women and men, which was found in all other age groups [3]. These findings were also in line with

For peer review only - http://bmjopen.bmj.com/site/about/guidelines.xhtml

the results from the 2017 survey "Vanor och konsekvenser" [29]. The difference in prevalence between the two other studies and our study, especially in the case of women, indicates that an individual may estimate their alcohol consumption as high without seeking care or compensation. The study by Andréasson et al. further shows that the youngest group (19–25 years) had the highest prevalence of alcohol use disorder (around 11%), while our study shows that the age group 50–59 years has the highest prevalence (3.9%). The registers (except the Register of convictions) are largely based on people have sought care, so the difference between the previous survey-based studies and the present study may be due to lower care seeking for substance use disorder amongst younger age groups compared to older groups. It could also be due to the fact that the same alcohol intake could have different consequences in different age groups. This would mean that a younger person drinking a hazardous amount might perceive this as unharmful, whereas an older person drinking the same amount may experience hazardous consequences more directly and is hence more prone to seek care. It was only in the register unrelated to care seeking, the Register of convictions, where the youngest age group had a higher likelihood compared to the older age groups.

We found that 2.5 % of men and 1.1 % of women had an indication for drug use disorder. This can be compared with the results from Central Association for Alcohol and Drug Information (CAN)-survey [29], where drug dependence according to the Diagnostic and Statistical Manual of Mental Disorders 5 (DSM-5) [30] amounted to 1.9 % among men and 1.6 % among women. The prevalence among men was thus higher according to register data than in the survey-based study, while the opposite applied to women. In the present study, the Register of convictions was central to an indication of drug use disorders, and it was more common for men than for women to occur in this register. Among men, the highest prevalence of an indication of drug use disorder was captured by the Register of convictions (1.7%), followed by the Outpatient register (1.1%). Among women, most were captured in the Outpatient and the Inpatient registers (0.6% and 0.4%, respectively), followed by the Register of convictions (0.3%). The correlation was greatest if an individual appeared in the Register of convictions, but this register also captured cases that were not in other registers. The vast coverage of this register was expected, as both drug possession and use are illegal in Sweden.

The prevalence of indications of alcohol and drug use disorder differ between the registers. For example, drug use disorder was captured by the Register of convictions to a greater extent than alcohol use disorder, which might be explained by the fact that alcohol is legal while drugs are illegal. This legal difference not only affects the availability of the substances and thus use, the problematic use and need for care, but also which register identifies it. In the case of alcohol use disorder, it was the Register of Medications that had the highest prevalence amongst both men and women. In addition, the Register of Medications showed a different pattern than the Outpatient and Inpatient care register, as among those with an indication of alcohol use disorder married individuals, those born in Sweden and those with a post-secondary education had a higher likelihood in this register, while these groups less likely to be found in the Outpatient and Inpatient registers. The finding that medication targeting alcohol use disorder are prescribed to a greater extent among people born in Sweden and among people with higher education, compared with migrants and those with a shorter education, is in line with previous studies [31].

Strengths and limitations

This study examines the proportion of individuals with an indication of alcohol or drug use disorder during a specific period (period prevalence) without concern of previous or repeated use of health care or the social insurance system. Individuals found in multiple registers likely have more severe alcohol and drug use disorder, but the focus here has been the prevalence of indications of substance use disorder. A valid dependence diagnosis can be described as a diagnosis that meets the

For peer review only - http://bmjopen.bmj.com/site/about/guidelines.xhtml

ICD-10 criteria for the specific addiction. The validity of indications of alcohol and drug use disorder in this study depends on the validity of the different registers. The diagnoses in the Inpatient and Outpatient registers were made by physicians and can thus be considered valid [19]. Being offered sickness or activity compensation by the Swedish Social Insurance Agency is preceded by a process in which physicians are involved, and although there are some differences in insurance medical practice over the country, these indications can also be considered valid. With regard to the Register of Medications, none of the medications against alcohol use disorder included in the study have other uses according to the Swedish Pharmacopeia Information [32] and were all prescribed by physicians, thus be considered valid markers of validity. Some of the medications included as a marker for drug use disorder in the study can in some special cases be used for pain relief, which reduces the validity.

With regard to the register of convictions, this differs from the other registers in that it only measures one aspect of substance use disorder, namely the ICD-10 criterion of continued drinking or use despite negative effects, and is therefore only a proxy variable, i.e. a variable that replaces another variable when it cannot be directly observed. It can thus be considered to have the lowest validity. Only when a person had more than two convictions for relevant alcohol or drug related crime during the same calendar year was this counted as an indication of substance use disorder. It is possible that more individuals with alcohol or drug use disorder would have been identified from the register of convictions if the threshold of number of convictions during a calendar had been lower, however this practice could also have increased the false positives and not according to the ICD-10 criteria of substance use disorder [33]. Getting three convictions for severe forms of alcohol-related crime is relatively rare, as for aggravated drunk driving there is a suspension period, i.e. a time when the driving license is revoked, which typically amounts to 12 months. Regarding the indication of drug use disorder in the register of criminal convictions, it is not certain that possession or sale of drugs involves either use or drug use disorder, although studies show that people who sell drugs usually do use drugs [34, 35].

Conclusion

The use of a combination of relevant population-registers improved the ability to describe the prevalence of alcohol and drug use disorder in the population. The increase in the proportion with an indication of substance use disorder was between 60 % (men with an indication of alcohol use disorder) to 80 % (women with an indication of drug use disorder) when the Inpatient and Outpatient register, the Register of Medications, the Social Insurance register and the Register for convictions were used together, compared to when only the register with the largest prevalence on its own was used. With combining more registers, indications of alcohol or drug use disorder among younger people and migrants, as well as married individuals and those with a higher education, increased when compared with using one register only. The study increases knowledge of how the use of different administrative registers creates a more accurate picture of the prevalence and differences in substance use disorder between different demographic groups of the population, although it is important to keep in mind the validity of the different registers. The results can contribute to the identification of target groups for preventive work and to the analysis of the consequences of alcohol and drug use disorder for different categories of the population. This is an important prerequisite for more equal health.

Declarations

<u>Ethics approval and consent to participate</u>

The project and the register linking were approved by the Regional Ethical Review Board in Stockholm (dnr. 2016 / 987-31).

For peer review only - http://bmjopen.bmj.com/site/about/guidelines.xhtml

Consent for publication

Not applicable

Availability of data and materials

Data is available on request for any interested researchers to allow replication of results through the Swedish National Data Service, provided all ethical and legal requirements are met. Detailed information on data application can be found at https://www.registerforskning.se/en/.

Competing interests

The authors declare that they have no competing interests

Funding

ACH is supported by FORTE (grant numbers 2016-00870). CD is supported by the Swedish Research Council (grant number 5232010-1052). This work was partly funded by the Public Health Agency of Sweden.

Authors' contributions

AL, ACH and CD conceived the study. AL and ACH designed the study. ACH obtained funding from FORTE and the Public Health Agency of Sweden. CD acquired the cohort data. AL prepared the data. AL wrote the study protocol, conducted the statistical analyses, and drafted the data tables. AL and ACH interpreted the statistical analyses. AKD and ACH wrote the manuscript. All authors critically revised the paper for important intellectual content and approved the final version.

Acknowledgements

Parts of the results of this study has been published with other research questions in a report published by the Public Health Agency of Sweden in Swedish (https://www.folkhalsomyndigheten.se/publicerat-material/publikationsarkiv/a/alkohol--och-narkotikaberoende/).

Patient involvement

No patients were involved in setting the research question or the outcome measures, nor were they involved in developing plans for design or implementation of the study. No patients were asked to advise on interpretation or writing up of results. However, we will disseminate the results of our research to agencies responsible for the healthcare of refugee and migrant groups in Sweden.

References

1. Degenhardt L, Charlson F, Ferrari A, Santomauro D, Erskine H, Mantilla-Herrara A, Whiteford H, Leung J, Naghavi M, Griswold M *et al*: **The global burden of disease attributable to alcohol and drug use in 195 countries and territories, 1990-2016: a systematic analysis for the Global Burden of Disease Study 2016**. *Lancet Psychiatry* 2018, **5**(12):987-1012.

2. Agardh E, Boman U, Allebeck P: **Alkohol, narkotika och tobaksrökning ger stor del av sjukdomsbördan - Utvecklingen i Sverige 1990–2010 kartlagd utifrån DALY-metoden**. *Läkartidningen* 2015, **2015;112:C4TH**.

3. Andreasson S, Danielsson AK, Hallgren M: **Severity of alcohol dependence in the Swedish adult population: association with consumption and social factors**. *Alcohol* 2013, **47**(1):21-25.

For peer review only - http://bmjopen.bmj.com/site/about/guidelines.xhtml

4. Kendler KS, Ohlsson H, Sundquist K, Sundquist J: **Prediction of drug abuse recurrence: a Swedish National Study**. *Psychological Medicine* 2018, **48**(8):1367-1374.

5. Syden L, Sidorchuk A, Makela P, Landberg J: **The contribution of alcohol use and other behavioural, material and social factors to socio-economic differences in alcohol-related disorders in a Swedish cohort**. *Addiction* 2017, **112**(11):1920-1930.

6. Hjern A, Allebeck P: **Alcohol-related disorders in first- and second-generation immigrants in Sweden: a national cohort study**. *Addiction* 2004, **99**(2):229-236.

7. Manhica H, Gauffin K, Almquist YB, Rostila M, Berg L, de Cortazar ARG, Hjern A: **Hospital admissions due to alcohol related disorders among young adult refugees who arrived in Sweden as teenagers - a national cohort study**. *Bmc Public Health* 2017, **17**.

8. Manhica H, Gauffin K, Almqvist YB, Rostila M, Hjern A: **Hospital Admission and Criminality Associated with Substance Misuse in Young Refugees - A Swedish National Cohort Study**. *Plos One* 2016, **11**(11).

9. Salvatore JE, Lonn SL, Sundquist J, Lichtenstein P, Sundquist K, Kendler KS: **Alcohol use disorder and divorce: evidence for a genetic correlation in a population-based Swedish sample**. *Addiction* 2017, **112**(4):586-593.

10. Harris S, Dykxhoorn J, Hollander AC, Dalman C, Kirkbride JB: **Substance use disorders in refugee and migrant groups in Sweden: A nationwide cohort study of 1.2 million people**. *PLoS Med* 2019, **16**(11):e1002944.

11. Stedman RC, Connelly NA, Heberlein TA, Decker DJ, Allred SB: **The End of the (Research) World As We Know It? Understanding and Coping With Declining Response Rates to Mail Surveys**. *Soc Natur Resour* 2019, **32**(10):1139-1154.

12. Platt B, O'Driscoll C, Curran VH, Rendell PG, Kamboj SK: **The effects of licit and illicit recreational drugs on prospective memory: a meta-analytic review**. *Psychopharmacology* 2019, **236**(4):1131-1143.

13. Allebeck P: **The use of population based registers in psychiatric research**. *Acta Psychiatr Scand* 2009, **120**(5):386-391.

14. Abel KM, Heuvelman HP, Jorgensen L, Magnusson C, Wicks S, Susser E, Hallkvist J, Dalman C: **Severe bereavement stress during the prenatal and childhood periods and risk of psychosis in later life: population based cohort study**. *BMJ* 2014, **348**:f7679.

15. Zammit S, Allebeck P, David AS, Dalman C, Hemmingsson T, Lundberg I, Lewis G: **A longitudinal study of premorbid IQ Score and risk of developing schizophrenia, bipolar disorder, severe depression, and other nonaffective psychoses**. *Arch Gen Psychiatry* 2004, **61**(4):354-360.

16. Runeson B, Tidemalm D, Dahlin M, Lichtenstein P, Langstrom N: **Method of attempted suicide as predictor of subsequent successful suicide: national long term cohort study**. *BMJ* 2010, **341**:c3222.

17. DeVilbiss EA, Magnusson C, Gardner RM, Rai D, Newschaffer CJ, Lyall K, Dalman C, Lee BK: **Antenatal nutritional supplementation and autism spectrum disorders in the Stockholm youth cohort: population based cohort study**. *BMJ* 2017, **359**:j4273.

For peer review only

For peer review only - http://bmjopen.bmj.com/site/about/guidelines.xhtml

18. Bould H, DeStavola B, Magnusson C, Micali N, Dal H, Evans J, Dalman C, Lewis G: **The influence of school in the development of eating disorders: a record-linkage study**. *Lancet* 2015, **385 Suppl 1**:S24.

19. Ludvigsson JF, Andersson E, Ekbom A, Feychting M, Kim JL, Reuterwall C, Heurgren M, Olausson PO: **External review and validation of the Swedish national inpatient register**. *BMC Public Health* 2011, **11**:450.

20. **https://www.socialstyrelsen.se/en/statistics-and-data/registers/register-information/the-national-patient-register/**

21. Hollander AC, Mackay E, Sjoqvist H, Kirkbride JB, Baarnhielm S, Dalman C: **Psychiatric care use among migrants to Sweden compared with Swedish-born residents: a longitudinal cohort study of 5 150 753 people**. *BMJ Glob Health* 2020, **5**(9).

22. Wandell P, Carlsson AC, Wettermark B, Lord G, Cars T, Ljunggren G: **Most common diseases diagnosed in primary care in Stockholm, Sweden, in 2011**. *Family Practice* 2013, **30**(5):506-513.

23. Statistics Sweden (SCB): **Longitudinell integrationsdatabas för sjukförsäkrings- och arbetsmarknadsstudier (LISA) 1990-2019 (Integrated database for labour market research)**. Stockholm: Statistics Sweden; 2019.

24. Statistics Sweden (SCB): **Dokumentation of databasen STATIV**. In. Edited by Sweden S. www.sos.se; 2019.

25. Statistics Sweden (SCB) **https://www.socialstyrelsen.se/en/statistics-and-data/registers/register-information/the-swedish-prescribed-drug-register/** 2020

26. Försäkringskassan **https://www.forsakringskassan.se/statistik**

27. **https://www.bra.se/bra-in-english/home.html** 2020

28. World Health Organization: **International statistical classification of diseases and related health problems : ICD-10. Vol. 1** 2. edn. Geneva: World Health Organization; 2004.

29. Landberg J, Ramstedt M, Sundin E: **Socioekonomiska skillnader i beroende och utsatthet för andras användning av alkohol, narkotika och tobak**. In: *CAN-rapport.* Edited by narkotikaupplysning Cfa-o; 2018: 91 sidor.

30. American Psychiatric Association, American Psychiatric Association. DSM-5 Task Force.: **Diagnostic and statistical manual of mental disorders : DSM-5**, 5. edn. Arlington, Va.: American Psychiatric Association; 2013.

31. Karriker-Jaffe KJ, Ji JG, Sundquist J, Kendler KS, Sundquist K: **Disparities in pharmacotherapy for alcohol use disorder in the context of universal health care: a Swedish register study**. *Addiction* 2017, **112**(8):1386-1394.

32. FASS: **https://www.fass.se/LIF/startpage**

33. World Health Organization: **International statistical classification of diseases and related health problems : ICD-10. Vol. 3 : alphabetical index**, 2. edn. Geneva: World Health Organization; 2004.

34. DeBeck K, Shannon K, Wood E, Li K, Montaner J, Kerr T: **Income generating activities of people who inject drugs**. *Drug Alcohol Depend* 2007, **91**(1):50-56.

For peer review only - http://bmjopen.bmj.com/site/about/guidelines.xhtml

35. Moyle L, Coomber R: **Earning a Score: An Exploration of the Nature and Roles of Heroin and Crack Cocaine 'User-Dealers'**. *Brit J Criminol* 2015, **55**(3):534-555.

For peer review only

For peer review only - http://bmjopen.bmj.com/site/about/guidelines.xhtml

The RECORD statement – checklist of items, extended from the STROBE statement, that should be reported in observational studies using routinely collected health data.

	Item No.	STROBE items	Location in manuscript where items are reported	RECORD items	Location in manuscript where items are reported
**Title and abstract**					
	1	(a) Indicate the study's design with a commonly used term in the title or the abstract (b) Provide in the abstract an informative and balanced summary of what was done and what was found	Title page at page 1 and abstract at page 2.	RECORD 1.1: The type of data used should be specified in the title or abstract. When possible, the name of the databases used should be included.<br><br>RECORD 1.2: If applicable, the geographic region and timeframe within which the study took place should be reported in the title or abstract.<br><br>RECORD 1.3: If linkage between databases was conducted for the study, this should be clearly stated in the title or abstract.	Title page at page 1 and abstract at page 2.
**Introduction**					
Background rationale	2	Explain the scientific background and rationale for the investigation being reported	Page 4		
Objectives	3	State specific objectives, including any prespecified hypotheses	Page 4		
**Methods**					
Study Design	4	Present key elements of study design early in the paper	Page 4		
Setting	5	Describe the setting, locations, and relevant dates, including periods of recruitment, exposure, follow-up, and data collection	Page 5		

For peer review only

Participants	6	*(a) Cohort study* - Give the eligibility criteria, and the sources and methods of selection of participants. Describe methods of follow-up<br>*Case-control study* - Give the eligibility criteria, and the sources and methods of case ascertainment and control selection. Give the rationale for the choice of cases and controls<br>*Cross-sectional study* - Give the eligibility criteria, and the sources and methods of selection of participants<br><br>*(b) Cohort study* - For matched studies, give matching criteria and number of exposed and unexposed<br>*Case-control study* - For matched studies, give matching criteria and the number of controls per case		RECORD 6.1: The methods of study population selection (such as codes or algorithms used to identify subjects) should be listed in detail. If this is not possible, an explanation should be provided.<br><br>RECORD 6.2: Any validation studies of the codes or algorithms used to select the population should be referenced. If validation was conducted for this study and not published elsewhere, detailed methods and results should be provided.<br><br>RECORD 6.3: If the study involved linkage of databases, consider use of a flow diagram or other graphical display to demonstrate the data linkage process, including the number of individuals with linked data at each stage.	Page 5<br><br>Page 5<br><br>Page 5
Variables	7	Clearly define all outcomes, exposures, predictors, potential confounders, and effect modifiers. Give diagnostic criteria, if applicable.	Page 5-6	RECORD 7.1: A complete list of codes and algorithms used to classify exposures, outcomes, confounders, and effect modifiers should be provided. If these cannot be reported, an explanation should be provided.	Page 5-6
Data sources/ measurement	8	For each variable of interest, give sources of data and details of methods of assessment (measurement).<br>Describe comparability of assessment methods if there is more than one group	Page 5-6		

For peer review only

Bias	9	Describe any efforts to address potential sources of bias	Page 5-6		
Study size	10	Explain how the study size was arrived at	page 5		
Quantitative variables	11	Explain how quantitative variables were handled in the analyses. If applicable, describe which groupings were chosen, and why	page 5-6		
Statistical methods	12	(a) Describe all statistical methods, including those used to control for confounding<br>(b) Describe any methods used to examine subgroups and interactions<br>(c) Explain how missing data were addressed<br>(d) *Cohort study* - If applicable, explain how loss to follow-up was addressed<br>*Case-control study* - If applicable, explain how matching of cases and controls was addressed<br>*Cross-sectional study* - If applicable, describe analytical methods taking account of sampling strategy<br>(e) Describe any sensitivity analyses	Page 7		
Data access and cleaning methods		..		RECORD 12.1: Authors should describe the extent to which the investigators had access to the database population used to create the study population.	Page 5-6

For peer review only

				RECORD 12.2: Authors should provide information on the data cleaning methods used in the study.	Page 5-6
Linkage		..		RECORD 12.3: State whether the study included person-level, institutional-level, or other data linkage across two or more databases. The methods of linkage and methods of linkage quality evaluation should be provided.	Page 5-6
**Results**					
Participants	13	(a) Report the numbers of individuals at each stage of the study (*e.g.*, numbers potentially eligible, examined for eligibility, confirmed eligible, included in the study, completing follow-up, and analysed)<br>(b) Give reasons for non-participation at each stage.<br>(c) Consider use of a flow diagram	Page 7	RECORD 13.1: Describe in detail the selection of the persons included in the study (*i.e.*, study population selection) including filtering based on data quality, data availability and linkage. The selection of included persons can be described in the text and/or by means of the study flow diagram.	Page 7
Descriptive data	14	(a) Give characteristics of study participants (*e.g.*, demographic, clinical, social) and information on exposures and potential confounders<br>(b) Indicate the number of participants with missing data for each variable of interest<br>(c) *Cohort study* - summarise follow-up time (*e.g.*, average and total amount)	page 7		
Outcome data	15	*Cohort study* - Report numbers of outcome events or summary measures over time<br>*Case-control study* - Report numbers in each exposure	Page 7		

For peer review only

		category, or summary measures of exposure<br>*Cross-sectional study* - Report numbers of outcome events or summary measures			
Main results	16	(a) Give unadjusted estimates and, if applicable, confounder-adjusted estimates and their precision (e.g., 95% confidence interval). Make clear which confounders were adjusted for and why they were included<br>(b) Report category boundaries when continuous variables were categorized<br>(c) If relevant, consider translating estimates of relative risk into absolute risk for a meaningful time period	page 11-12		
Other analyses	17	Report other analyses done—e.g., analyses of subgroups and interactions, and sensitivity analyses	Page 11-12		
**Discussion**					
Key results	18	Summarise key results with reference to study objectives	Page 13		
Limitations	19	Discuss limitations of the study, taking into account sources of potential bias or imprecision. Discuss both direction and magnitude of any potential bias	Page 14	RECORD 19.1: Discuss the implications of using data that were not created or collected to answer the specific research question(s). Include discussion of misclassification bias, unmeasured confounding, missing data, and changing eligibility over time, as they pertain to the study being reported.	Page 14
Interpretation	20	Give a cautious overall interpretation of results considering objectives,	Page 13		

For peer review only

		limitations, multiplicity of analyses, results from similar studies, and other relevant evidence			
Generalisability	21	Discuss the generalisability (external validity) of the study results	Page 14		
**Other Information**					
Funding	22	Give the source of funding and the role of the funders for the present study and, if applicable, for the original study on which the present article is based	Page 16		
Accessibility of protocol, raw data, and programming code		..	Page 16	RECORD 22.1: Authors should provide information on how to access any supplemental information such as the study protocol, raw data, or programming code.	Page 16

*Reference: Benchimol EI, Smeeth L, Guttmann A, Harron K, Moher D, Petersen I, Sørensen HT, von Elm E, Langan SM, the RECORD Working Committee. The REporting of studies Conducted using Observational Routinely-collected health Data (RECORD) Statement. *PLoS Medicine* 2015; in press.

*Checklist is protected under Creative Commons Attribution (CC BY) license.

For peer review only

BMJ Open

Indications of alcohol- or drug use disorders in five different national registers in Sweden – a cross sectional population-based study.

Journal:	*BMJ Open*
Manuscript ID	bmjopen-2022-070744.R1
Article Type:	Original research
Date Submitted by the Author:	29-Jun-2023
Complete List of Authors:	Lundin, Andreas; Karolinska Institutet, Department of Global Public Health ; Centre for Epidemiology and Community Medicine, Region Stockholm<br>Danielsson, Anna-Karin; Karolinska Institutet, Global Public Health<br>Dalman, Christina; Karolinska Institute, Global Public Health ; Centre for Epidemiology and Community Medicine, Region Stockholm<br>Hollander, Anna-Clara; Karolinska Institutet, Department of Global Public Health
**Primary Subject Heading**:	Addiction
Secondary Subject Heading:	Epidemiology, Mental health, Public health
Keywords:	EPIDEMIOLOGY, Substance misuse < PSYCHIATRY, PUBLIC HEALTH, SOCIAL MEDICINE

SCHOLARONE™
Manuscripts

I, the Submitting Author has the right to grant and does grant on behalf of all authors of the Work (as defined in the below author licence), an exclusive licence and/or a non-exclusive licence for contributions from authors who are: i) UK Crown employees; ii) where BMJ has agreed a CC-BY licence shall apply, and/or iii) in accordance with the terms applicable for US Federal Government officers or employees acting as part of their official duties; on a worldwide, perpetual, irrevocable, royalty-free basis to BMJ Publishing Group Ltd ("BMJ") its licensees and where the relevant Journal is co-owned by BMJ to the co-owners of the Journal, to publish the Work in this journal and any other BMJ products and to exploit all rights, as set out in our licence.

The Submitting Author accepts and understands that any supply made under these terms is made by BMJ to the Submitting Author unless you are acting as an employee on behalf of your employer or a postgraduate student of an affiliated institution which is paying any applicable article publishing charge ("APC") for Open Access articles. Where the Submitting Author wishes to make the Work available on an Open Access basis (and intends to pay the relevant APC), the terms of reuse of such Open Access shall be governed by a Creative Commons licence – details of these licences and which Creative Commons licence will apply to this Work are set out in our licence referred to above.

Other than as permitted in any relevant BMJ Author's Self Archiving Policies, I confirm this Work has not been accepted for publication elsewhere, is not being considered for publication elsewhere and does not duplicate material already published. I confirm all authors consent to publication of this Work and authorise the granting of this licence.

For peer review only

Title page

The title: Indications of alcohol- or drug use disorders in five different national registers in Sweden – a cross sectional population-based study.

List the full names and institutional addresses for all authors:

Andreas Lundin[1,2]

Anna-Karin Danielsson[1]

Christina Dalman[1,2]

Anna-Clara Hollander[1]*

[1]Epidemiology of Psychiatric Conditions, Substance Use and Social Environment (EPiCSS), Department of Global Public Health, KI, Solnavägen 1E, 171 77 Stockholm, Sweden

[2]Centre for Epidemiology and Community Medicine, Region Stockholm, Stockholm, Sweden

*the corresponding author: anna-clara.hollander@ki.se

For peer review only

For peer review only - http://bmjopen.bmj.com/site/about/guidelines.xhtml

Abstract

Objective: The purpose of this study is to examine the prevalence of indications of alcohol- or drug use disorders in five different national Swedish registers and to investigate the correlation between these registers. Furthermore, to investigate if combining data from different registers increases the prevalence of these indications in the population, due to registers identifying different demographic groups.

Design: Cross-sectional study

Setting and participants: Individuals living in Sweden aged 20 – 64 in 2006, n = 5 453 616.

Primary outcome: The national registers were the registers of Inpatient care, Outpatient, Medications, Social Insurance and Convictions. Demographic variables were sex, age, migrant status, education, and civil status. Indications of alcohol or drug use disorders were presented as prevalence in percentage (%), correlation was examined with a phi-correlation coefficients, and the differences across demographic factors were studied using logistic regression.

Results: The prevalence of an indication of alcohol- or drug use disorder varied between registers, meaning that the prevalence increased when all registers were considered together. The prevalence of alcohol use disorder increased by 60 % and 66 % amongst men and women, respectively, while the prevalence of drug use disorder increased by 45 % and 80 % amongst men and women, respectively, when all registers were combined compared to solely using the register with the highest prevalence. The registers contributed to indications of alcohol and drugs use disorder differently.

Conclusions: Having accurate estimates of alcohol or drug use disorders is critical for health care and rehabilitation. This study shows that using a single register alone underestimates the burden of disease unevenly, while combining a range of registers can provide a more accurate picture.

Keywords

alcohol use disorder; drug use disorder; substance use disorder; health-care registers, population based; epidemiology

Word count: 4927

For peer review only - http://bmjopen.bmj.com/site/about/guidelines.xhtml

Strengths and limitations of this study

- The study has a large sample size as it included all individuals living in Sweden aged 20–64 in 2006 (n = 5 453 616).
- The five national registers included in the study are of high quality.
- A limitation of the study is that it examines the period prevalence of substance use disorder and does not take previous use into account.
- The validity of indications of alcohol- and drug use disorder depends on the validity of the different registers.

For peer review only

For peer review only - http://bmjopen.bmj.com/site/about/guidelines.xhtml

Background

Alcohol and drug use cause a substantial disease burden globally, the composition and extent of which varies between countries [1]. Globally, in 2016, 4.2 % of all disability-adjusted life year (DALYs) were attributable to alcohol use, and 1.3 % to drug use [1] and the lost DALYs were due to the risk of injury, premature death, and other negative consequences [2]. However, the risk posed by using alcohol and drugs differs between men and women, by socioeconomic conditions, and for migrants by country of birth [3-5]. The term substance use disorder refers to both substance abuse and substance dependence and although studies of it in a population commonly use surveys [3, 6], registry-based studies provide an additional way of studying substance use disorders [7-9]. There are many advantages to using survey data in studies of alcohol and drug use disorders in a population, such as the ability to study different degrees of the disorder as the participants describe their consumption and their related problems themselves. However, disadvantages of surveys include: decreasing response rate in population surveys in general [10], selection bias, as some socioeconomic groups are less likely to respond to surveys than others, and that heavy substance use can affect memory [11], increasing the risk of recall bias.

In Scandinavia and Finland, health care visits are recorded in local and national administrative registers covering the entire population. Such population-based registers have benefited psychiatric research immensely [12]. These health registers are generally considered valid [13], but using registers collected for administrative purposes in research can give misleading results if there is a systematic underutilisation of care in specific sub-groups. In Sweden to date, studies of substance use disorder have used the National Patient Register [4, 7-9] from the perspective that the most severe cases of substance use disorder will require care. This register has been an important source of knowledge about the social determinants and consequences of severe alcohol- and drug use disorder. However, it has long been recognised that, in addition to detecting the most severe cases only, these registers possibly underestimate severe cases in specific sub-groups where under-utilisation is pronounced [14]. As register-based research could be a powerful tool for population-based studies of substance use disorders, there have been attempts to expand the use of population-registers by including additional registers with possibly less severe cases, such as registers with diagnoses from General practitioners (GP) surgeries [15], the Crime Prevention Council's register [16], and the Register of prescribed and purchased medicine [17]. Studies using the additional registers recognise that appearing in the additional registers is not the same as being diagnosed with a substance use disorder but acknowledge that it is a possible indication of a substance use disorder. However, despite being used in studies of the determinants and consequences of a substance use disorder, to date this practice has not been tested empirically.

The purpose of this study is to examine the prevalence of indications of alcohol- or drug use disorders in five different national Swedish registers. This is in order to investigate the extent of correlation between the registers to find out if combining the registers increases the prevalence of such indications in the population, due to registers identifying different demographic groups such as different age-groups, sexes, educational level, migrant or marital status.

Methods

<u>Design</u>

We conducted a historical cross-sectional register-based study in several Swedish national registries. To link registers, we used the personal identity numbers that are uniquely assigned to every resident at birth or immigration.

For peer review only - http://bmjopen.bmj.com/site/about/guidelines.xhtml

Data sources

Data were extracted from a linked register database called Psychiatry Sweden (PS) held by the research group Epidemiology of Psychiatric Conditions, Substance use and Social environment (EPiCSS). PS consists of several national and regional registers.

For the registers of alcohol and drug use disorders we used three registers from the National Board of Health and Welfare: the Patient register that actually contains two registers; one for inpatient care (henceforth called the Inpatient register) and one for specialized outpatient care (henceforth called the Outpatient register). Data on psychiatric inpatient care has been accessible here since 1973 and there has been good coverage of psychiatric outpatient data since 2006 [18]. The Register of prescribed and purchased pharmaceuticals (Läkemedelsregistret) (accessible since 2006) [19], is a register of all medicines prescribed in health care and dispensed at pharmacies (henceforth called the Register of Medications). We also included the Swedish Social Insurance Agency's (Försäkringskassan) Mikrodata for analysis of social insurance (henceforth called the Social Insurance register) (accessible since 1992) [20] were we used the variables Sickness absence and Disability pension. Sickness absence refers to the temporarily reduced work capacity due to alcohol and drug use disorders. Disability pension is a public financial support provided to the individuals who permanently leave the labour market before the age of statutory retirement usually preceded by sick leave. From the Crime Prevention Council's register of convicted of crime (Brottsförebyggande rådets (BRÅs)) Register över lagförda personer [21] (since 2000) we used the Register of prosecuted convictions based on the information registered at the Swedish Public Prosecutor's Office and courts (henceforth called the Register of Convictions).

In addition, we used registers from Statistics Sweden for the demographic factors the Register of the total population, the Longitudinal integration database for health insurance and labour market studies (LISA) (since 1990) [22], the Longitudinal database for integration studies STATIV (since 1997) [23].

Population

The population consisted of all persons in Sweden aged 20–64 during 2006 and was identified in LISA. The lower age limit was determined by not having access to data from the child and adolescents' psychiatry from all over Sweden, and the higher as persons over 64 cannot be included in the register of Social Insurance due to the Swedish age of retirement in 2006. The population were then followed up in various registers regarding the indication of alcohol and drug use disorder between 2006 and 2016.

Definitions of indication of alcohol or drug use disorder

The present study focused on use so extensive and problematic that it was captured in national registers and will henceforth be called a register indication of alcohol or drug use disorder. The registers primarily use the International Statistical Classification of Diseases and Related Health Problems, 10th Edition (ICD-10) [24] system of classification where alcohol and drug use disorder has six possible criteria:

- Craving
- Loss of control
- Prioritization of alcohol/drug consumption over other activities
- Continued intake despite adverse effects
- Increased tolerance

For peer review only - http://bmjopen.bmj.com/site/about/guidelines.xhtml

- Abstinence

For a person to be diagnosed according to ICD-10, three of six criteria must be met during the past year [24].

Alcohol use disorder

In both the Patient registers and the Social Insurance register, an indication of alcohol use disorder was an ICD-10 diagnosis of "Mental disorders and behavioural disorders caused by alcohol (F10)".

In the Register of Convictions, an indication of alcohol use disorder was defined as relevant alcohol-related crimes such as drunk driving, aggravated drunk driving or care of intoxicated persons (Act (1951: 649) on penalties for certain traffic offenses, § 4 drunk driving and § 4a aggravated drunk driving respectively). Since one of the ICD-10 criteria for alcohol use disorders is still drinking despite negative effects, it was only counted as a register indication if an individual had more than two convictions during the same calendar year.

In the Register of Medications, an indication of alcohol use disorder was defined as having been prescribed medicine against alcohol use disorder including Disulfiram (ATC NO7BB01), Acamprosate (ATC NO7BB03), Naltrexone (ATC NO7BB04) and Nalmefene (ACT NO7BB05).

Drug use disorder

In the Patient registers and the Social Insurance register, an indication of drug use disorder was an ICD-10 diagnosis of chapters F11 – F14, F16, F18 – F19, which are Mental disorders and behavioural disorders caused by:

- opioids (F11)
- cannabinoids (F12)
- sedatives or hypnotics (F13)
- cocaine (F14)
- hallucinogens (F16)
- volatile solvents (F18)
- psychoactive substances (F19)

In the Register of Convictions, an indication of drug use disorder was relevant drug-related crime (Narcotics Penal Code (1968: 64) and the Act (1991: 1969) on the prohibition of certain substances such as possession, use of, or other engagement with drugs. As one of the ICD-10 criteria for drug use disorder is continued use despite negative effects, it was only counted as a register indication when a person had more than two convictions during the same calendar year.

In the Register of Medications, an indication of drug use disorder was having been prescribed and dispensed Buprenorphine N07BC01, Methadone N07BC02, Levomethadone N07BC05 and Buprenorphine, combinations N07BC51 (only drugs for opiate dependence were available).

<u>Co-variates</u>

- Sex was defined as sex assigned at birth: male or female.
- Age was categorised in age groups: 20–29, 30–39, 40–49, 50–59 and 60–64.
- Migration status was grouped into those born in Sweden, migrants, and refugees; the refugees were defined as having received a residence permit for refugee reasons according to the Swedish Migration Agency.

For peer review only - http://bmjopen.bmj.com/site/about/guidelines.xhtml

- Education level was grouped according to Swedish education nomenclature as pre-secondary, upper secondary, post-secondary, and unknown.
- Marital status was grouped as married (including registered partnership), divorced (including deregistered partnership), widow / widower and unmarried.

Statistical analysis

Alcohol and drug use disorder in the registers were presented as cumulative period prevalence and reported in percentage. Correlation, i.e. the extent to which those with an indication of substance use disorder in one register appear in other registers, was examined with phi-correlation for dichotomous variables. Phi correlation is interpreted as a scale where the correlation coefficient is between 1 and -1, where 1 means maximum positive correlation, 0 is no correlation at all, and -1 is maximum negative correlation. Relationships between the demographic variables (age, sex, migrant status, level of education, marital status) and indication of alcohol or drug use disorder in different registers were examined using logistic regression and presented as odds ratios (OR) and 95 % confidence interval (CI). All regression models were multivariate models including the variables age, sex, migrant status, education level and marital status.

Results

The population consisted of all individuals in Sweden aged 20–64 in 2006, a total of 5.45 million individuals, and is described in Table 1.

Table 1. Characterization of the population in 2006 in percent (%).

**Characteristics**	**Total**	**N = 5 453 616**
		%
**Sex**	Male	51
	Female	49
**Migration status**	Born in Sweden	84
	Born abroad	15
	Refugee	2
**Education**	Pre-upper secondary school	16
	Upper secondary	48
	Post-secondary school	34
	Missing	2
**Age group**	20–29	20
	30–39	23
	40–49	23
	50–59	22
	60–64	13
**Marital status**	Married	43
	Unmarried	43
	Divorced	12
	Widow widower	1

Table 2 shows the prevalence of an indication of alcohol use disorder, by sex and age in the population. The prevalence of individuals with a register indication for alcohol use disorder differed between registers. In total, 2.0 % of the women and 4.2 % of the men appeared in one of these registers. Due to the doubled prevalence of an indication among men, as compared with women, table 2 is also displayed by men and women separately, see supplementary file, supplementary table 1 and supplementary table 2. Those aged 50–59 years had the highest prevalence in all registers except the Register of Convictions. The highest prevalence for both men and women were found in

For peer review only - http://bmjopen.bmj.com/site/about/guidelines.xhtml

the Register of Medications (men 2.6 %, women 1.2 %), followed by the Inpatient and Outpatient register and the Social Insurance register. The lowest prevalence was found in the Register of Convictions. Comparing all registers combined with the register with the highest prevalence increased total prevalence by 60 % for men and 66 % for women. Prevalence in the different age groups increased correspondingly from 24 % to 67 %, with the largest increase among people aged 30–39 years. Table 2 also shows the correlation between the registers i.e. the extent to which persons with an indication of alcohol use disorder in one register appear in other registers, and the unique contribution in percent of each register to a register indication. Register indication in the In- and Outpatient register and the Register of medications had the highest correlation, still these three registers contributed with the largest proportion of unique indications. The correlation between having an indication in another register were strongest if a person appeared in the Register of medications and lowest if the person appeared in The Register of Convictions. The registers of Social insurance and Register of Convictions had the smallest unique contribution.

For peer review only

Table 2. Indication of alcohol use disorder in the total population. Total number n with indication, percent % with register indication by age-group, migrant status, education, marital status, phi - the correlation coefficient between the registers and the unique contribution of the specific register to all registers combined (Any registers) for the following registers: Inpatient care, Outpatient care, the Register of Medications (Medications), the Social Insurance register (Social Insurance), the Register of Convictions (Convictions), and Any register.

	**Register**	In-patient	Out-patient	Medications	Social Insurance	Convictions	Any registers
**Total number**	n	66 940	96 279	103 199	9 323	1 837	167 178
		%	%	%	%	%	%
**Sex**	Men	1.69	2.37	2.59	0.23	0.06	4.15
	Women	0.75	1.15	1.17	0.11	0.01	1.95
**Age**	20–29	0.91	1.58	1.10	0.06	0.04	2.59
	30–39	0.78	1.33	1.35	0.12	0.03	2.25
	40–49	1.27	1.91	2.17	0.24	0.04	3.22
	50–59	1.66	2.25	2.57	0.29	0.04	3.91
	60–64	1.37	1.67	1.89	0.10	0.02	3.13
**Migrant status**	Born in Sweden	1.25	1.80	2.00	0.18	0.03	3.16
	Born abroad	1.16	1.68	1.42	0.12	0.04	2.68
	Refugee	0.52	0.89	0.62	0.04	0.02	1.44
**Education**	Education Pre-upper	2.35	3.05	3.03	0.25	0.09	5.23
	Upper second	1.36	1.92	2.08	0.21	0.03	3.36
	Post-second	0.53	0.96	1.13	0.09	0.01	1.67
	Miss	0.84	1.11	0.87	0.03	0.04	1.87
**Martial status**	Married	0.62	0.96	1.30	0.10	0.01	1.87
	Unmarried	1.42	2.09	2.00	0.19	0.05	3.48
	Divorced	2.69	3.46	3.61	0.37	0.06	5.82
	Widow(er)	1.55	1.93	2.15	0.15	0.03	3.53
**Correlation coefficient**	Inpatient	x	0.54	0.41	0.25	0.04	0.63
	Outpatient		x	0.49	0.22	0.04	0.75
	Medications			x	0.22	0.04	0.78
	Social Insurance				x	0.02	0.23
	Convictions					x	0.10
	Any registers						x
**Unique contribution to Any register**		9.01	17.66	27.13	0.48	0.60	x

Individuals with an indication of alcohol use disorder were n = 167 175 and differed by age, sex, migrant status, education level and marital status (see Table 3). Due to this doubled prevalence among men table 3 is also displayed by men and women separately see supplementary file, supplementary table 3 and supplementary table 4. Women with an indication of alcohol use disorder appeared more often in the Outpatient register (8 % more likely than men) but had a lower likelihood of being found in the Inpatient register (4 % less likely than men), Register of Medications (12 % less likely than men) and Register of Convictions (74% less likely than men) (see Table 3). Both for the

For peer review only - http://bmjopen.bmj.com/site/about/guidelines.xhtml

total population with an indication of alcohol use disorders, and men and women separately, the likelihood of having an indication in the Inpatient register increased with age and the likelihood of an indication in the Register of Convictions decreased with age. With regards to the Outpatient register those with an indication of alcohol use disorder aged 20–29 or 40–49 years were more likely to be found in the compared to those in other age-groups for men but this was not seen in women. Both for the total population with an indication of alcohol use disorders, and men and women separately aged 40–49 and 50–59 years appeared more often in the Register of Medications (Table 3, see supplementary file, supplementary table 3 and supplementary table 4). Migrants with an indication of alcohol use disorder were more likely to appear in the Inpatient and Outpatient register, and they were also more likely to have an indication in the Register of Convictions, compared with those born in Sweden, with little difference between men and women. Persons born in Sweden with an indication of alcohol use disorder, on the other hand, were more likely to appear in the Register of Medications and in the Social Insurance register compared with migrants (Table 3). People with an indication of alcohol use disorder and with post-secondary education appeared more often in the Register of Medications and the Outpatient register compared with other education levels. However, among those with an indication of alcohol use disorder and pre-secondary education only, it was more common to appear in the Register of Convictions and in the Inpatient register. People with an indication of alcohol use disorder who were married were more likely to be found in in the Register of Medications than individuals who were unmarried, divorced, and widows and widowers (see Table 3).

For peer review only

Table 3. The association between socioeconomic factors and appearing in specific registers among those with an indication of alcohol use disorder (n=167175) by the following registers: the Inpatient care, the Outpatient care, the Register of Medications (Medications), the Social Insurance register (Social Insurance) and the Register of Convictions (Convictions) presented with odds ratios (OR) with 95 % confidence interval (CI), by age group, sex, migrant status, education level and marital status all included in the multivariate models.

		Inpatient	Outpatient	Medications	Social Insurance	Convictions
		OR (95 % CI)	OR (95 % CI)	OR (95 % CI)	OR (95 % CI)	OR (95 % CI)
**Age-group**	**20–29**	0.62 (0.60–0.65)	1.07 (1.03–1.11)	0.46 (0.45–0.48)	0.30 (0.27–0.34)	1.63 (1.38–1.93)
	**30–39**	0.67 (0.65–0.69)	1.02 (0.99–1.06)	0.86 (0.83–0.89)	0.70 (0.65–0.75)	1.53 (1.33–1.78)
	**40–49**	0.87 (0.85–0.90)	1.07 (1.04–1.10)	1.11 (1.08–1.15)	1.01 (0.96–1.07)	1.34 (1.17–1.52)
	**50–59**	1	1	1	1	1
	**60–64**	1.09 (1.06–1.13)	0.88 (0.86–0.91)	0.74 (0.72–0.76)	0.43 (0.40–0.46)	0.64 (0.54–0.75)
**Sex**	**Female**	0.96 (0.94–0.98)	1.08 (1.06–1.10)	0.88 (0.86–0.90)	0.99 (0.94–1.03)	0.26 (0.22–0.30)
	**Male**	1	1	1	1	1
**Migrant status**	**Migrant**	1.15 (1.12–1.19)	1.29 (1.25–1.33)	0.63 (0.61–0.65)	0.77 (0.72–0.83)	1.27 (1.12–1.45)
	**Refugee**	0.93 (0.83–1.04)	1.25 (1.12–1.40)	0.39 (0.35–0.44)	0.46 (0.34–0.64)	1.28 (0.84–1.97)
	**Sweden**	1	1	1	1	1
**Education**	**Pre-upper secondary school**	1.17 (1.15–1.20)	1.04 (1.01–1.06)	0.90 (0.88–0.92)	0.80 (0.76–0.84)	1.58 (1.43–1.75)
	**Upper secondary**	1	1	1	1	1
	**Post-secondary school**	0.69 (0.67–0.71)	1.06 (1.03–1.09)	1.23 (1.19–1.26)	0.87 (0.82–0.92)	0.59 (0.49–0.70)
	**Missing**	1.30 (1.17–1.43)	0.98 (0.88–1.08)	0.81 (0.73–0.89)	0.40 (0.28–0.58)	1.82 (1.31–2.52)
**Marital status**	**Single**	1.56 (1.52–1.60)	1.39 (1.35–1.43)	0.64 (0.62–0.66)	1.11 (1.05–1.18)	1.29 (1.13–1.48)
	**Married**	1	1	1	1	1
	**Divorced**	1.62 (1.58–1.67)	1.39 (1.35–1.42)	0.73 (0.71–0.75)	1.25 (1.18–1.32)	1.56 (1.34–1.81)
	**Widow/Widower**	1.41 (1.29–1.53)	1.18 (1.09–1.28)	0.79 (0.73–0.86)	1.06 (0.87–1.30)	1.69 (1.06–2.71)

For peer review only

The prevalence of individuals with an indication of drug use disorder in any of the registers was 1.1 % amongst women and 2.5 % amongst men. The group 20–29 of age had the highest prevalence in all registers except in the Register of medications (Table 4). The prevalence of an indication of drug use disorder was consistently higher for men than women. Due to the more than doubled prevalence among men table 4 is also displayed by men and women separately, see supplementary file, supplementary table 5 and supplementary table 6. For all registers, except in the Register of Medications, prevalence decreased with age. For men, the highest prevalence was found in the Register of Convictions (1.7 %), followed by the Outpatient and Inpatient register, and in the Register of Medications. For women, the highest prevalence was found in the Outpatient register (0.6 %), followed by the Inpatient register, the Register of Convictions and in the Register of Medications. Both men and women had the lowest prevalence in the Social Insurance register. For drug use disorder, the prevalence increased by 45 % for men and 80 % for women if all registers were combined, instead of the register with the highest prevalence alone. The prevalence in different age groups increased correspondingly by between 30 % to 109 %, with the largest increase among people in the 50–59 age group.

There was a correlation between having an indication of a drug use disorder between the registers. The correlation was highest between the Inpatient and Outpatient register (0.56). The correlation between having an indication in another register was strongest if the individual appeared in the Register of Convictions (0.76) and lowest if they appeared in the Social Insurance register (0.19). The register contributing with the highest proportion of individual cases was for men was the Register of Convictions but the registers of Outpatient care for women. The register contributing with the smallest proportion was the Register of Social Insurance register for both sexes.

For peer review only

Table 4. Indication of drug use disorder in the total population. Total number n with indication, percent % with register indication by age-group, migrant status, education, marital status, phi - the correlation coefficient between the registers and the unique contribution of the specific register to all registers combined (Any registers) for the following registers: Inpatient care, Outpatient care, the Register of Medications (Medications), the Social Insurance register (Social Insurance), the Register of Convictions (Convictions), and Any register.

	**Register**	Inpatient	Outpatient	Medications	Social Insurance	Convictions	Any registers
**Total number**	n	32 175	46 123	13 293	3 559	57 211	99 552
**Sex**	Men	0.74	1.07	0.29	0.09	1.74	2.53
	Women	0.44	0.61	0.19	0.04	0.34	1.10
**Age**	20–29	1.21	1.71	0.21	0.16	3.20	4.15
	30–39	0.74	1.08	0.25	0.10	1.47	2.23
	40–49	0.61	0.88	0.25	0.06	0.90	1.73
	50–59	0.46	0.67	0.26	0.04	0.57	1.40
	60–64	0.21	0.29	0.24	0.01	0.09	0.69
**Migrant status**	Born in Sweden	0.59	0.83	0.24	0.07	0.97	1.74
	Born abroad	0.58	0.87	0.23	0.05	1.40	2.18
	Refugee	0.77	1.30	0.45	0.06	2.05	3.01
**Education**	Education Pre-upper	1.45	1.99	0.55	0.12	2.73	4.12
	Upper second	0.58	0.84	0.24	0.07	1.07	1.91
	Post-second	0.19	0.30	0.11	0.03	0.18	0.59
	Miss	0.68	0.95	0.17	0.03	1.55	2.21
**Martial status**	Married	0.21	0.30	0.17	0.02	0.21	0.68
	Unmarried	0.91	1.31	0.28	0.11	1.91	2.85
	Divorced	0.84	1.17	0.37	0.07	1.04	2.36
	Widow(er)	0.37	0.52	0.32	0.02	0.23	1.02
**Correlation coefficient**	Inpatient	x	0.56	0.23	0.20	0.33	0.56
	Outpatient		x	0.27	0.21	0.36	0.68
	Medications			x	0.10	0.15	0.36
	Social Insurance				x	0.12	0.19
	Convictions					x	0.76
	Any registers						x
**Unique contribution to Any register**		7.99	15.99	6.31	0.51	35.88	x

Individuals who were found to have an indication of drug use disorder (n=99 529) differed by age, sex, migrant status, education level and marital status (see Table 5). Due to the more than doubled prevalence among men table 5 is also displayed by men and women separately, see supplementary file, supplementary table 7 and supplementary table 8. In terms of sex differences among those with an indication of drug use disorder, women were more likely to appear in the Outpatient and Inpatient registers as well as the Register of Medications compared with men, while men were more likely to appear in the Register of Convictions. Those aged 20–29 years and with an indication of drug

For peer review only - http://bmjopen.bmj.com/site/about/guidelines.xhtml

use disorder were more likely to appear in the Register of Convictions. Persons with an indication of drug use disorder aged 30–49 were more likely than other groups to be found both in the Register of Convictions (together with those aged 20-29) and to have an indication in the Social Insurance register. The oldest age group (60–64 years) with an indication of drug use disorder were more likely to appear in the Register of Medications (see Table 5). Male migrants with an indication of drug use disorder were more likely to appear in the Register of Convictions compared with individuals born in Sweden, but this was not seen among women (see supplementary file, supplementary table 7 and supplementary table 8). Those with a pre-secondary education were more likely to appear in all registers, except the Social Insurance register, compared to those with a higher education. Unmarried and divorced individuals with an indication of drug use disorder were more likely to appear in the Inpatient register and in the Register of Convictions compared with married individuals. Married individuals with an indication of drug use disorders appeared more often than other marital statuses in the Register of Medications.

For peer review only

For peer review only - http://bmjopen.bmj.com/site/about/guidelines.xhtml

Table 5. The association between socioeconomic factors and appearing in specific registers among those with an indication of drug use disorder n=99529 by the following registers: the Inpatient care, the Outpatient care, the Register of Medications (Medications), the Social Insurance register (Social Insurance) and the Register of Convictions (Convictions) and any register that are all registers combined presented with odds ratios (OR) with 95 % confidence interval (CI), including age group, sex, migrant status, education level and marital status in the in the multivariate models.

		**Inpatient**	**Outpatient**	**Medications**	**Social Insurance**	**Convictions**
		OR (95 % CI)	OR (95 % CI)	OR (95 % CI)	OR (95 % CI)	OR (95 % CI)
**Age-group**	**20–29**	0.86 (0.82–0.90)	0.77 (0.74–0.80)	0.26 (0.24–0.28)	1.74 (1.53–1.97)	3.82 (3.64–4.01)
	**30–39**	1.07 (1.02–1.12)	1.05 (1.01–1.09)	0.57 (0.54–0.61)	2.00 (1.78–2.26)	2.57 (2.46–2.69)
	**40–49**	1.13 (1.08–1.18)	1.13 (1.09–1.18)	0.73 (0.69–0.77)	1.49 (1.32–1.68)	1.61 (1.54–1.69)
	**50–59**	1	1	1	1	1
	**60–64**	0.86 (0.81–0.91)	0.74 (0.70–0.78)	1.97 (1.85–2.09)	0.33 (0.26–0.42)	0.25 (0.23–0.27)
**Sex**	**Female**	1.61 (1.56–1.66)	1.69 (1.64–1.74)	1.27 (1.22–1.32)	1.07 (1.00–1.16)	0.22 (0.22–0.23)
	**Male**	1	1	1	1	1
**Migrant status**	**Born abroad**	0.72 (0.69–0.75)	0.74 (0.71–0.76)	0.73 (0.69–0.77)	0.58 (0.52–0.64)	1.38 (1.32–1.43)
	**Refugee**	0.73 (0.67–0.80)	0.90 (0.83–0.97)	1.20 (1.08–1.35)	0.50 (0.38–0.65)	1.40 (1.28–1.54)
	**Sweden**	1	1	1	1	1
**Education**	**Pre-upper secondary school**	1.30 (1.26–1.34)	1.25 (1.22–1.29)	1.21 (1.16–1.27)	0.74 (0.69–0.80)	1.44 (1.39–1.49)
	**Upper secondary**	1	1	1	1	1
	**Post-secondary school**	1.01 (0.96–1.05)	1.20 (1.15–1.26)	1.13 (1.06–1.20)	1.36 (1.23–1.50)	0.46 (0.44–0.49)
	**Missing**	1.27 (1.15–1.41)	1.23 (1.12–1.34)	0.89 (0.75–1.05)	0.36 (0.25–0.54)	1.22 (1.09–1.36)
**Marital status**	**Single**	1.14 (1.09–1.19)	1.19 (1.14–1.24)	0.56 (0.53–0.59)	0.91 (0.82–1.01)	2.15 (2.05–2.25)
	**Married**	1	1	1	1	1
	**Divorced**	1.25 (1.19–1.31)	1.20 (1.15–1.26)	0.51 (0.48–0.54)	0.95 (0.83–1.08)	2.12 (2.01–2.23)
	**Widow/ Widower**	1.17 (1.00–1.37)	1.21 (1.04–1.41)	0.87 (0.74–1.03)	1.05 (0.63–1.74)	1.72 (1.41–2.10)

For peer review only - http://bmjopen.bmj.com/site/about/guidelines.xhtml

Discussion

The study demonstrates that the proportion with an indication of alcohol or drug use disorder differs between registers but that there is a correlation between the registers. This means that those with a register indication of substance use in one register sometimes, but not always, appear in one or more other registers, hence the proportion increases when registers are added together. The proportion in the population with an indication of alcohol or drug use disorders increase by between 45 – 80 % when compared with only the register showing the highest prevalence. For alcohol use disorder the three health care registers i.e., the Inpatient and the Outpatient registers and the Register of medications had the highest correlations, however, despite this correlation the three health care registers also had the largest proportions of unique alcohol use disorder indications. The registers for Social Insurance and Convictions only contribute with less than one percent each. Still, if not including the Social Insurance register this could bias the results towards missing indications of alcohol use disorders among individuals who are single or divorced. If excluding the Register of Convictions this could bias the results towards missing indication of alcohol use disorder among the younger (up to 49), those not born in Sweden, those without a higher education and not currently married. For drug use disorder the highest correlation with other registers was when an individual appeared in the Outpatient register or the Register of Convictions. These two registers also contribute with most independent indications. The Social Insurance register contribute with less than one percent, however, if not including it among the registers this could bias the results towards missing indications of drug use disorders among individual younger than 49 years and with a higher education.

Findings in context

In this study, using all registers showed the proportion of the population with an indication of alcohol use disorder as 4.2 % for men and 2.0 % for women. This can be compared to a cross-sectional study by Andréasson et al. based on survey-data from randomly selected 19 to 70-year-olds from 12 Swedish municipalities [3], where researchers found that the proportion classified as having an alcohol use disorder was 4.9 % for men and 3.2 % for women. The same study also found that an average of 4 % of the population in Sweden meet at least three criteria for alcohol use disorder, while among 19 to 25-year-olds the prevalence was higher (around 11 %) and in this group there was no statistical difference between women and men, which was found in all other age groups [3]. These findings were also in line with the results from the 2017 survey “Vanor och konsekvenser” [in English “Habits and consequences”][6]. The difference in prevalence between the two other studies and our study, especially in the case of women, indicates that an individual may estimate their alcohol consumption as high without seeking care. The study by Andréasson et al. further shows that the youngest group (19–25 years) had the highest prevalence of alcohol use disorder (around 11 %), while our study shows that the age group 50–59 years has the highest prevalence (3.9 %). The registers (except the Register of Convictions) are largely based on people have been utilizing health care, so the difference between the previous survey-based studies and the present study may be due to lower care seeking for substance use disorder amongst younger age groups compared to older groups. It could also be due to the fact that the same alcohol intake could have different consequences in different age groups. This would mean that a younger person drinking a hazardous amount might perceive this as unharmful, whereas an older person drinking the same amount may experience hazardous consequences more directly and is hence more prone to seek care. It was only in the register unrelated to care seeking, the Register of Convictions, where the youngest age group had a higher likelihood compared to the older age groups.

For peer review only - http://bmjopen.bmj.com/site/about/guidelines.xhtml

We found that 2.5 % of men and 1.1 % of women had an indication for drug use disorder. This can be compared with the results from Central Association for Alcohol and Drug Information (CAN)-survey [6], where drug dependence according to the Diagnostic and Statistical Manual of Mental Disorders 5 (DSM-5) [25] amounted to 1.9 % among men and 1.6 % among women. The prevalence among men was thus higher according to register data than in the survey-based study, while the opposite applied to women. In the present study, the Register of Convictions was central to an indication of drug use disorders, and it was more common for men than for women to occur in this register. Among men, the highest prevalence of an indication of drug use disorder was captured by the Register of Convictions (1.7 %), followed by the Outpatient register (1.1 %). Among women, most were captured in the Outpatient and the Inpatient registers (0.6 % and 0.4 %, respectively), followed by the Register of Convictions (0.3 %). The correlation was strongest if an individual appeared in the Register of Convictions, but this register also captured cases that were not in other registers. The vast coverage of this register was expected, as both drug possession and use are illegal in Sweden.

The prevalence of indications of alcohol and drug use disorder differ between the registers. For example, drug use disorder was captured by the Register of Convictions to a stronger extent than alcohol use disorder, which might be explained by the fact that alcohol is legal while drugs are illegal. This legal difference not only affects the availability of the substances and thus use, the problematic use and need for care, but also which register identifies it. In the case of alcohol use disorder, it was the Register of Medications that had the highest prevalence amongst both men and women. In addition, the Register of Medications showed a different pattern than the Outpatient and Inpatient care register. Among those with an indication of alcohol use disorder married individuals, those born in Sweden and those with a post-secondary education had a higher likelihood to be found in this register, but were less likely to be found in the Outpatient and Inpatient registers. The finding that medications targeting alcohol use disorder are prescribed to a stronger extent among people born in Sweden and among people with higher education, compared with migrants and those with a shorter education, is in line with previous studies [26].

Strengths and limitations

This study examines the proportion of individuals with an indication of alcohol or drug use disorder during a specific period (period prevalence) without concern of previous or repeated use of health care or the social insurance system. Individuals found in multiple registers are likely having more severe alcohol and drug use disorder. Although adding all the registers together increase the prevalence of indications of substance use disorders, this study probably underestimates the prevalence of an early stage of substance use disorder as those at this stage possibly seek less care and might be more careful in terms of, for instance, drunk driving. Also, we have not included diagnoses from primary care, thus some individuals seeking care at an early stage may not be detected. However, as we include the register of Medications where all prescribed medications are registered, including medications prescribed in primary, this underestimation of patients in primary care is limited to patients who have not been purchasing prescribed medications from GPs in primary care nor been referred to outpatient care. Another limitation is the age span 20-64 as both alcohol and drug use disorders are common among adolescents and young adults and among elderly.

A valid substance use diagnosis can be described as a diagnosis that meets the ICD-10 criteria. The validity of indications of alcohol and drug use disorder in this study depends on the validity of the different registers. The diagnoses in the Inpatient and Outpatient registers were made by physicians and can thus be considered valid [13]. Being offered sickness or activity compensation by the Swedish Social Insurance Agency is preceded by a process in which physicians are involved, and although there are some differences in insurance medical practice over the country, these indications

For peer review only - http://bmjopen.bmj.com/site/about/guidelines.xhtml

can also be considered valid. With regard to the Register of Medications, none of the medications against alcohol use disorder included in the study have other uses according to the Swedish Pharmacopeia Information [27] and were all prescribed by physicians, thus be considered valid. Some of the medications included as a marker for drug use disorder in the study can in some special cases be used for pain relief, which reduces the validity. With regard to the Register of Convictions, this differs from the other registers in that it only measures one aspect of substance use disorder, namely the ICD-10 criterion of continued drinking or use despite negative effects, and is therefore only a proxy variable, i.e. a variable that replaces another variable when it cannot be directly observed. It can thus be considered to have the lowest validity. Only when a person had more than two convictions for relevant alcohol or drug related crime during a calendar year was this counted as an indication of substance use disorder. More individuals with alcohol or drug use disorder would have been identified from the register of Convictions if the threshold of number of convictions during a calendar had been lower, however this practice could also have increased the false positives and not according to the ICD-10 criteria of substance use disorder [24]. Getting three convictions for severe forms of alcohol-related crime is relatively rare, as for aggravated drunk driving there is a suspension period, i.e. a time when the driving license is revoked, which typically amounts to 12 months. Regarding the indication of drug use disorder in the Register of Convictions, it is not certain that possession or sale of drugs involves either use or drug use disorder, although studies show that people who sell drugs usually do use drugs [28, 29]. The Register of Convictions were used as proxy variable and there are other proxy variables that could have been included too such as intoxication (from the Inpatient or Outpatient register) were more than two times could be an indicator of possible SUD.

Conclusion

The conclusion of this study is that the use of a combination of relevant population-registers improves the ability to describe the prevalence of alcohol and drug use disorder in the population. Another effect of combining more registers is that indications of alcohol or drug use disorder among younger people and migrants, as well as married individuals and those with a higher education, increased as compared with using one register only. The registers contribute differently to the indications of alcohol and drugs use disorder. For instance, the Register of Convictions contribute with less than one percent of the unique indications of alcohol use disorder but contribute with more than 35 % of the unique indications of drug use disorders. The study increases knowledge of how the use of different administrative registers creates a more accurate picture of the prevalence and differences in substance use disorder between different demographic groups of the population, although it is important to keep in mind the validity of the different registers.

For peer review only - http://bmjopen.bmj.com/site/about/guidelines.xhtml

Declarations

Ethics approval and consent to participate

The project and the register linking were approved by the Regional Ethical Review Board in Stockholm (dnr. 2016 / 987-31).

Consent for publication

Not applicable

Availability of data and materials

Data is available on request for any interested researchers to allow replication of results through the Swedish National Data Service, provided all ethical and legal requirements are met. Detailed information on data application can be found at https://www.registerforskning.se/en/.

Competing interests

The authors declare that they have no competing interests

Funding

ACH is supported by the Swedish council for Health, Working Life and Welfare (FORTE, by Swedish acronym) (grant numbers 2016-00870). CD is supported by the Swedish Research Council (grant number 5232010-1052). This work was partly funded by the Public Health Agency of Sweden.

Authors' contributions

AL, ACH and CD conceived the study. AL and ACH designed the study. ACH obtained funding from FORTE and the Public Health Agency of Sweden. CD acquired the cohort data. AL prepared the data. AL wrote the study protocol, conducted the statistical analyses, and drafted the data tables. AL and ACH interpreted the statistical analyses. AKD and ACH wrote the manuscript. All authors critically revised the paper for important intellectual content and approved the final version.

Acknowledgements

Parts of the results of this study has been published with other research questions in a report published by the Public Health Agency of Sweden in Swedish (https://www.folkhalsomyndigheten.se/publicerat-material/publikationsarkiv/a/alkohol--och-narkotikaberoende/).

Patient involvement

No patients were involved in setting the research question or the outcome measures, nor were they involved in developing plans for design or implementation of the study. No patients were asked to advise on interpretation or writing up of results. However, we will disseminate the results of our research to agencies responsible for the healthcare of refugee and migrant groups in Sweden.

References

1. Degenhardt L, Charlson F, Ferrari A, Santomauro D, Erskine H, Mantilla-Herrara A, Whiteford H, Leung J, Naghavi M, Griswold M *et al*: **The global burden of disease attributable to alcohol and drug use in 195 countries and territories, 1990-2016: a systematic analysis for the Global Burden of Disease Study 2016**. *Lancet Psychiatry* 2018, **5**(12):987-1012.

For peer review only - http://bmjopen.bmj.com/site/about/guidelines.xhtml

2. Agardh E, Boman U, Allebeck P: **Alkohol, narkotika och tobaksrökning ger stor del av sjukdomsbördan - Utvecklingen i Sverige 1990–2010 kartlagd utifrån DALY-metoden**. *Läkartidningen* 2015, **2015;112:C4TH**.
3. Andreasson S, Danielsson AK, Hallgren M: **Severity of alcohol dependence in the Swedish adult population: association with consumption and social factors**. *Alcohol* 2013, **47**(1):21-25.
4. Kendler KS, Ohlsson H, Sundquist K, Sundquist J: **Prediction of drug abuse recurrence: a Swedish National Study**. *Psychological Medicine* 2018, **48**(8):1367-1374.
5. Syden L, Sidorchuk A, Makela P, Landberg J: **The contribution of alcohol use and other behavioural, material and social factors to socio-economic differences in alcohol-related disorders in a Swedish cohort**. *Addiction* 2017, **112**(11):1920-1930.
6. Landberg J, Ramstedt M, Sundin E: **Socioekonomiska skillnader i beroende och utsatthet för andras användning av alkohol, narkotika och tobak**. In: *CAN-rapport.* Edited by narkotikaupplysning Cfa-o; 2018: 91 sidor.
7. Hjern A, Allebeck P: **Alcohol-related disorders in first- and second-generation immigrants in Sweden: a national cohort study**. *Addiction* 2004, **99**(2):229-236.
8. Manhica H, Gauffin K, Almquist YB, Rostila M, Berg L, de Cortazar ARG, Hjern A: **Hospital admissions due to alcohol related disorders among young adult refugees who arrived in Sweden as teenagers - a national cohort study**. *Bmc Public Health* 2017, **17**.
9. Harris S, Dykxhoorn J, Hollander A-C, Dalman C, Kirkbride JB: **Substance use disorders in refugee and migrant groups in Sweden: A nationwide cohort study of 1.2 million people**. *Plos Medicine* 2019, **16**(11).
10. Stedman RC, Connelly NA, Heberlein TA, Decker DJ, Allred SB: **The End of the (Research) World As We Know It? Understanding and Coping With Declining Response Rates to Mail Surveys**. *Soc Natur Resour* 2019, **32**(10):1139-1154.
11. Platt B, O'Driscoll C, Curran VH, Rendell PG, Kamboj SK: **The effects of licit and illicit recreational drugs on prospective memory: a meta-analytic review**. *Psychopharmacology* 2019, **236**(4):1131-1143.
12. Allebeck P: **The use of population based registers in psychiatric research**. *Acta Psychiatr Scand* 2009, **120**(5):386-391.
13. Ludvigsson JF, Andersson E, Ekbom A, Feychting M, Kim JL, Reuterwall C, Heurgren M, Olausson PO: **External review and validation of the Swedish national inpatient register**. *BMC Public Health* 2011, **11**:450.
14. Hollander AC, Mackay E, Sjoqvist H, Kirkbride JB, Baarnhielm S, Dalman C: **Psychiatric care use among migrants to Sweden compared with Swedish-born residents: a longitudinal cohort study of 5 150 753 people**. *BMJ Glob Health* 2020, **5**(9).
15. Wandell P, Carlsson AC, Wettermark B, Lord G, Cars T, Ljunggren G: **Most common diseases diagnosed in primary care in Stockholm, Sweden, in 2011**. *Family Practice* 2013, **30**(5):506-513.
16. Manhica H, Gauffin K, Almqvist YB, Rostila M, Hjern A: **Hospital Admission and Criminality Associated with Substance Misuse in Young Refugees - A Swedish National Cohort Study**. *PLoS One* 2016, **11**(11):e0166066.
17. Salvatore JE, Larsson Lonn S, Sundquist J, Lichtenstein P, Sundquist K, Kendler KS: **Alcohol use disorder and divorce: evidence for a genetic correlation in a population-based Swedish sample**. *Addiction* 2017, **112**(4):586-593.
18. **www.socialstyrelsen.se**
19. **www.scb.se**
20. **https://www.forsakringskassan.se/statistik**
21. Bråttsförebyggande rådet B: **https://www.bra.se/bra-in-english/home.html** 2023.
22. Statistics Sweden S: **Longitudinell integrationsdatabas för sjukförsäkrings- och arbetsmarknadsstudier (LISA) 1990-2019 (Integrated database for labour market research)**. Stockholm: Statistics Sweden; 2019.

For peer review only - http://bmjopen.bmj.com/site/about/guidelines.xhtml

23. Sweden S: **Dokumentation of databasen STATIV**. In. Edited by Sweden S. www.sos.se; 2019.
24. World Health Organization: **International statistical classification of diseases and related health problems : ICD-10. Vol. 1 : tabular list**, 2. edn. Geneva: World Health Organization; 2004.
25. American Psychiatric Association., American Psychiatric Association. DSM-5 Task Force.: **Diagnostic and statistical manual of mental disorders DSM-5**. In., 5th edn. Arlington, VA: American Psychiatric Association,; 2013: 1 electronic resource.
26. Karriker-Jaffe KJ, Ji JG, Sundquist J, Kendler KS, Sundquist K: **Disparities in pharmacotherapy for alcohol use disorder in the context of universal health care: a Swedish register study**. *Addiction* 2017, **112**(8):1386-1394.
27. **https://www.fass.se/LIF/startpage**
28. DeBeck K, Shannon K, Wood E, Li K, Montaner J, Kerr T: **Income generating activities of people who inject drugs**. *Drug Alcohol Depend* 2007, **91**(1):50-56.
29. Moyle L, Coomber R: **Earning a Score: An Exploration of the Nature and Roles of Heroin and Crack Cocaine 'User-Dealers'**. *Brit J Criminol* 2015, **55**(3):534-555.

For peer review only

Supplementary tables

Supplementary table 1 Alcohol use disorder in the male population. Total number (N) with indication, percent % and number per cell (n) with register indication by age-group, migrant status, education, marital status, phi - the correlation coefficient between the registers and the unique contribution of the specific register to all registers combined (Any registers) for the following registers: Inpatient care, Outpatient care, the Register of Medications (Medications), the Social Insurance register (Social Insurance), the Register of convictions (Convictions), and Any register.

		Inpatient	Outpatient	Medication	Social Insurance	Convictions	Any registers
		N=46889	N=65445	N=71680	N=6415	N=1642	N=114789
		% (n)	% (n)	% (n)	% (n)	% (n)	% (n)
**Age**	**20–29**	1.09 (3627)	1.87 (6209)	1.45 (4821)	0.08 (259)	0.08 (251)	3.16 (10530)
	**30–39**	1.01 (6033)	1.73 (10297)	1.83 (10881)	0.15 (898)	0.06 (333)	2.98 (17699)
	**40–49**	1.69 (11050)	2.49 (16257)	2.90 (18887)	0.31 (2038)	0.07 (441)	4.26 (27768)
	**50–59**	2.37 (13889)	3.08(18097)	3.52 (20678)	0.40 (2320)	0.07 (396)	5.38 (31563)
	**60–65**	2.05 (12290)	2.43 (14585)	2.74 (16413)	0.15 (900)	0.04 (221)	4.54 (27229)
**Migration status**	**Born in Sweden**	1.73 (40370)	2.40 (55988)	2.74(63916)	0.25 (5748)	0.06 (1345)	4.27 (99770)
	**Born abroad**	1.63 (6133)	2.34 (8798)	1.94 (7295)	0.17 (632)	0.07 (275)	3.71 (13944)
	**Refugee**	0.69 (386)	1.18 (659)	0.84 (469)	0.06 (35)	0.04 (22)	1.92 (1075)
**Education**	**Education Pre-upper**	3.03 (15314)	3.88 (19623)	3.98 (20116)	0.33 (1671)	0.14 (687)	6.75 (34084)
	**Upper second**	1.83 (24993)	2.51 (34331)	2.79 (38145)	0.28 (3774)	0.06 (802)	4.43 (60676)
	**Post-second**	0.71 (6023)	1.28 (10749)	1.52 (12833)	0.11 (947)	0.01 (115)	2.23 (18772)
	**Miss**	1.14 (559)	1.51 (742)	1.19 (586)	0.05 (23)	0.08 (38)	2.56 (1257)
**Marital status**	**Married**	0.84 (9605)	1.29 (14734)	1.85 (21070)	0.13 (1453)	0.02 (267)	2.58 (29405)
	**Unmarried**	1.89 (24986)	2.69 (35455)	2.64 (34762)	0.25 (3311)	0.08 (992)	4.52 (59662)
	**Divorced**	4.07 (11824)	5.04 (14650)	5.22 (15183)	0.55 (1602)	0.13 (369)	8.48 (24635)
	**Widow(er)**	2.77 (474)	3.54 (606)	3.89 (665)	0.29 (49)	0.08 (14)	6.36 (1087)
**Unique contribution to Any register**	**%**	8.74 (10037)	16.55 (19001)	27.57 (31653)	0.46 (531)	0.19 (98)	-

For peer review only - http://bmjopen.bmj.com/site/about/guidelines.xhtml

Supplementary table 2 Alcohol use disorder in the female population. Total number (N) with indication, percent % and number per cell (n) with register indication by age-group, migrant status, education, marital status, phi - the correlation coefficient between the registers and the unique contribution of the specific register to all registers combined (Any registers) for the following registers: Inpatient care, Outpatient care, the Register of Medications (Medications), the Social Insurance register (Social Insurance), the Register of convictions (Convictions), and Any register.

		**Inpatient**	**Outpatient**	**Medication**	**Social Insurance**	**Convictions**	**Any registers**
		n=20051	n=30834	n=31519	n=2908	n=195	n=52389
**Age**	**20–29**	0.72 (2290)	1.28 (4073)	0.74 (2361)	0.04 (126)	0.01 (20)	1.99 (6317)
	**30–39**	0.53 (3020)	0.91 (5213)	0.86 (4899)	0.08 (447)	0.01 (44)	1.49 (8562)
	**40–49**	0.84 (5253)	1.30 (8173)	1.42 (8917)	0.16 (1017)	0.01 (64)	2.15 (13461)
	**50–59**	0.95 (5437)	1.39 (7984)	1.60 (9215)	0.17 (999)	0.01 (46)	2.42 (13885)
	**60–65**	0.68 (4051)	0.90 (5391)	1.03 (6127)	0.05 (319)	0.00 (21)	1.71 (10164)
**Migration status**	**Born in Sweden**	0.76 (16946)	1.17 (26229)	1.23 (27502)	0.12 (2581)	0.01 (182)	2.01 (44962)
	**Born abroad**	0.73 (3024)	1.08 (4458)	0.95 (3927)	0.08 (324)	0.00 (13)	1.74 (7204)
	**Refugee**	0.24 (81)	0.43 (147)	0.26 (90)	0.01 (3)	0.00 (0)	0.65 (223)
**Education**	**Education Pre-upper**	1.44 (5505)	1.94 (7409)	1.78 (6792)	0.13 (506)	0.02 (69)	3.24 (12375)
	**Upper second**	0.85 (10640)	1.28 (16093)	1.31 (16417)	0.13 (1692)	0.01 (84)	2.18 (27397)
	**Post-second**	0.37 (3697)	0.70 (7053)	0.80 (8096)	0.07 (702)	0.00 (40)	1.21 (12164)
	**Miss**	0.49 (209)	0.66 (279)	0.50 (214)	0.02 (8)	0.00 (2)	1.07 (453)
**Marital status**	**Married**	0.42 (5082)	0.66 (8031)	0.80 (9748)	0.07 (821)	0.00 (45)	1.22 (14853)
	**Unmarried**	0.82 (8644)	1.35 (14143)	1.20 (12556)	0.12 (1214)	0.01 (102)	2.17 (22792)
	**Divorced**	1.58 (5707)	2.18 (7911)	2.31 (8369)	0.23 (815)	0.01 (43)	3.69 (13352)
	**Widow(er)**	1.16 (618)	1.41 (749)	1.59 (846)	0.11 (58)	0.01 (5)	2.62 (1392)
**Unique contribution to Any register**	**%**	9.60 (5029)	20.09 (10527)	26.16 (13705)	0.53 (277)	0.19 (98)	-

For peer review only - http://bmjopen.bmj.com/site/about/guidelines.xhtml

Supplementary table 3 The association between socioeconomic factors and appearing in specific registers among men with an indication of alcohol use disorder (n= 114 789) by the following registers: the Inpatient care, the Outpatient care, the Register of Medications (Medications), the Social Insurance register (Social Insurance) and the Register of convictions (Convictions), presented with odds ratios (OR) with 95 % confidence interval (CI), by age group, sex, migrant status, education level and marital status all included in the multivariate models.

		Inpatient	Outpatient	Medication	Social Insurance	Convictions
**Age-group**	**20–29**	0.55 (0.53-0.58)	0.98 (0.94-1.03)	0.54 (0.52-0.57)	0.33 (0.29-0.38)	1.78 (1.49-2.11)
	**30–39**	0.60 (0.58-0.63)	0.98 (0.95-1.02)	0.94 (0.91-0.98)	0.69 (0.64-0.75)	1.55 (1.33-1.81)
	**40–49**	0.82 (0.80-0.85)	1.04 (1.01-1.08)	1.17 (1.13-1.21)	1.00 (0.94-1.07)	1.32 (1.15-1.52)
	**50–59**	1	1	1	1	1
	**60–64**	1.12 (1.08-1.15)	0.89 (0.86-0.92)	0.73 (0.71-0.76)	0.43 (0.4-0.47)	0.64 (0.54-0.76)
**Migrant status**	**Migrant**	1.15 (1.11-1.19)	1.35 (1.30-1.40)	0.60 (0.57-0.62)	0.78 (0.72-0.85)	1.4 (1.22-1.6)
	**Refugee**	0.93 (0.82-1.06)	1.24 (1.10-1.41)	0.37 (0.33-0.42)	0.51 (0.36-0.71)	1.39 (0.9-2.13)
	**Sweden**	1	1	1	1	1
**Education**	**Pre-upper secondary school**	1.14 (1.11-1.17)	1.03 (1.01-1.06)	0.92 (0.89-0.94)	0.84 (0.79-0.89)	1.55 (1.39-1.72)
	**Upper secondary**	1	1	1	1	1
	**Post-secondary school**	0.69 (0.67-0.72)	1.10 (1.06-1.13)	1.19 (1.15-1.24)	0.83 (0.77-0.9)	0.51(0.42-0.62)
	**Missing**	1.26 (1.12-1.42)	0.97 (0.86-1.09)	0.76 (0.68-0.86)	0.4 (0.26-0.6)	1.75 (1.25-2.46)
**Marital status**	**Married**	1	1	1	1	1
	**Single**	1.74 (1.69-1.80)	1.45 (1.41-1.50)	0.57 (0.55-0.59)	1.13 (1.06-1.21)	1.3 (1.12-1.5)
	**Divorced**	1.77 (1.71-1.84)	1.47 (1.42-1.52)	0.65 (0.63-0.68)	1.33 (1.24-1.44)	1.64 (1.4-1.92)
	**Widow/Widower**	1.36 (1.21-1.54)	1.33 (1.17-1.50)	0.72 (0.63-0.82)	1.19 (0.89-1.6)	1.72 (1-2.96)

For peer review only

Supplementary table 4 The association between socioeconomic factors and appearing in specific registers among women with an indication of alcohol use disorder (n= 52 389) by the following registers: the Inpatient care, the Outpatient care, the Register of Medications (Medications), the Social Insurance register (Social Insurance) and the Register of convictions (Convictions), presented with odds ratios (OR) with 95 % confidence interval (CI), by age group, sex, migrant status, education level and marital status all included in the multivariate models.

		Inpatient	Outpatient	Medication	Social Insurance	Convictions
**Age-group**	**20–29**	0.81 (0.76-0.87)	1.24 (1.16-1.33)	0.33 (0.31-0.35)	0.26 (0.22-0.32)	0.74 (0.42-1.3)
	**30–39**	0.85 (0.80-0.90)	1.11 (1.05-1.18)	0.70 (0.66-0.74)	0.71 (0.63-0.8)	1.39 (0.9-2.15)
	**40–49**	1.00 (0.95-1.05)	1.13 (1.08-1.19)	1.00 (0.95-1.06)	1.04 (0.95-1.14)	1.4 (0.95-2.06)
	**50–59**	1	1	1	1	1
	**60–64**	1.03 (0.98-1.09)	0.86 (0.82-0.91)	0.75 (0.71-0.79)	0.43 (0.38-0.49)	0.6 (0.36-1.02)
**Migrant status**	**Migrant**	1.16 (1.10-1.22)	1.19 (1.13-1.26)	0.71(0.67-0.75)	0.76 (0.68-0.86)	0.44 (0.25-0.77)
	**Refugee**	0.97 (0.73-1.27)	1.30 (0.99-1.72)	0.48 (0.36-0.63)	0.24 (0.08-0.75)	-
	**Sweden**	1	1	1	1	1
**Education**	**Pre-upper secondary school**	1.26 (1.21-1.31)	1.04 (1.00-1.09)	0.87 (0.84-0.91)	0.7 (0.63-0.78)	1.93 (1.4-2.66)
	**Upper secondary**	1	1	1	1	1
	**Post-secondary school**	0.69 (0.66-0.72)	1.01 (0.97-1.05)	1.27 (1.21-1.33)	0.93 (0.85-1.02)	1.11(0.76-1.63)
	**Missing**	1.40 (1.16-1.69)	0.98 (0.81-1.19)	0.95 (0.78-1.15)	0.42 (0.21-0.85)	2.1 (0.51-8.7)
**Marital status**	**Married**	1	1	1	1	1
	**Single**	1.23 (1.17-1.29)	1.27 (1.21-1.33)	0.83 (0.79-0.87)	1.09 (0.99-1.2)	1.29 (0.89-1.88)
	**Divorced**	1.37 (1.31-1.44)	1.24 (1.18-1.30)	0.87 (0.83-0.92)	1.1 (1-1.22)	1.06 (0.7-1.62)
	**Widow/Widower**	1.42 (1.27-1.59)	1.08 (0.96-1.20)	0.87 (0.78-0.98)	0.95 (0.72-1.26)	1.48 (0.58-3.8)

For peer review only - http://bmjopen.bmj.com/site/about/guidelines.xhtml

Supplementary table 5 Drug use disorder in the male population. Total number (N) with indication, percent % and number per cell (n) with register indication by age-group, migrant status, education, marital status, phi - the correlation coefficient between the registers and the unique contribution of the specific register to all registers combined (Any registers) for the following registers: Inpatient care, Outpatient care, the Register of Medications (Medications), the Social Insurance register (Social Insurance), the Register of convictions (Convictions), and Any register.

	**Inpatient**	**Outpatient**	**Medication**	**Social Insurance**	**Convictions**	**Any registers**
	n= 20365	n= 29671	n= 8117	n= 2449	n= 48155	n= 69928
**20–29**	1.60 (5314)	2.33 (7740)	0.26 (875)	0.23 (777)	5.30 (17643)	6.28 (20889)
**30–39**	0.98 (5808)	1.44 (8551)	0.33 (1979)	0.15 (877)	2.48 (14738)	3.25 (19323)
**40–49**	0.73 (4744)	1.07 (6964)	0.30 (1951)	0.08 (495)	1.43 (9331)	2.25 (14642)
**50–59**	0.55 (3258)	0.81 (4728)	0.31 (1793)	0.04 (264)	0.94 (5511)	1.81 (10620)
**60–65**	0.21 (1241)	0.28 (1688)	0.25 (1519)	0.01 (36)	0.16 (932)	0.74 (4454)
**Born in Sweden**	0.72 (16895)	1.03 (24099)	0.28 (6545)	0.09 (2115)	1.56 (36426)	2.33 (54457)
**Born abroad**	0.77 (2908)	1.22 (4574)	0.32 (1210)	0.08 (286)	2.66 (9978)	3.48 (13084)
**Refugee**	1.00 (562)	1.78 (998)	0.65 (362)	0.09 (48)	3.13 (1751)	4.27 (2387)
**Education Pre-upper**	1.74 (8767)	2.42 (12204)	0.64 (3231)	0.16 (819)	3.97 (20080)	5.31 (26807)
**Upper second**	0.70 (9593)	1.04 (14240)	0.28 (3842)	0.10 (1364)	1.76 (24123)	2.60 (35597)
**Post-second**	0.19 (1605)	0.32 (2671)	0.11 (954)	0.03 (246)	0.32 (2725)	0.71 (5994)
**Miss**	0.81 (400)	1.13 (556)	0.18 (90)	0.04 (20)	2.50 (1227)	3.11 (1530)
**Married**	0.20 (2321)	0.31 (3558)	0.19 (2176)	0.02 (271)	0.36 (4067)	0.79 (9019)
**Unmarried**	1.15 (15198)	1.67 (22078)	0.35 (4569)	0.15 (1921)	2.95 (3891)	3.93 (51823)
**Divorsed**	0.95 (2771)	1.35 (3927)	0.45 (1307)	0.09 (251)	1.75 (5100)	3.05 (8861)
**Widow(er)**	0.44 (75)	0.63 (108	0.38 (65)	0.04 (6)	0.44 (76)	1.32 (225)
**Unique contribution to Any register**	5.35 (3744)	11.92 (8332)	4.48 (3131)	0.39 (273)	44.08 (30825)	

For peer review only - http://bmjopen.bmj.com/site/about/guidelines.xhtml

<u>Supplementary table 6</u> Drug use disorder in the female population. Total number (N) with indication, percent % and number per cell (n) with register indication by age-group, migrant status, education, marital status, phi - the correlation coefficient between the registers and the unique contribution of the specific register to all registers combined (Any registers) for the following registers: Inpatient care, Outpatient care, the Register of Medications (Medications), the Social Insurance register (Social Insurance), the Register of convictions (Convictions), and Any register.

	**Inpatient**	**Outpatient**	**Medication**	**Social Insurance**	**Convictions**	**Any registers**
	n= 11810	n= 16452	n= 5176	n= 1110	n= 9056	n= 29624
**20–29**	0.81 (2565)	1.06 (3363)	0.15 (479)	0.08 (255)	0.99 (3145)	1.92 (6095)
**30–39**	0.50 (2861)	0.70 (4020)	0.16 (912)	0.06 (338)	0.43 (2459)	1.17 (6703)
**40–49**	0.48 (3022)	0.68 (4237)	0.19 (1203)	0.05 (319)	0.35 (2196)	1.18 (7418)
**50–59**	0.36 (2084)	0.54 (3083)	0.22 (1281)	0.03 (158)	0.19 (1115)	0.99 (5659)
**60–65**	0.21 (1278)	0.29 (1749)	0.22 (1301)	0.01 (40)	0.02 (141)	0.63 (3749)
**Born in Sweden**	0.45 (10002)	0.62 (13989)	0.20 (4539)	0.04 (993)	0.35 (7910)	1.12 (25177)
**Born abroad**	0.40 (1677)	0.55 (2292)	0.14 (595)	0.03 (110)	0.25 (1054)	1.00 (4125)
**Refugee**	0.38 (131)	0.50 (171)	0.12 (42)	0.02 (7)	0.27 (92)	0.94 (322)
**Education Pre-upper**	1.07 (4100)	1.44 (5498)	0.42 (1621)	0.07 (255)	1.09 (4170)	2.55 (9750)
**Upper second**	0.45 (5621)	0.62 (7830)	0.20 (2458)	0.05 (579)	0.32 (4074)	1.15 (14422)
**Post-second**	0.19 (1870)	0.28 (2808)	0.10 (1035)	0.03 (270)	0.06 (618)	0.49 (4954)
**Miss**	0.52 (219)	0.74 (316)	0.15 (62)	0.01 (6)	0.46 (194)	1.17 (498)
**Married**	0.21 (2575)	0.29 (3601)	0.15 (1854)	0.02 (242)	0.08 (1000)	0.58 (7031)
**Unmarried**	0.60 (6312)	0.85 (8911)	0.19 (2034)	0.06 (677)	0.60 (6305)	1.48 (15566)
**Divorsed**	0.76 (2737)	1.02 (3680)	0.31 (1126)	0.05 (181)	0.46 (1662)	1.80 (6533)
**Widow(er)**	0.35 (186)	0.49 (260)	0.30 (162)	0.02 (10)	0.17 (89)	0.93 (494)
**Unique contribution to Any register**	14.20 (4204)	25.60 (7583)	10.64 (3152)	0.79 (233)	16.53 (4898)	--

For peer review only - http://bmjopen.bmj.com/site/about/guidelines.xhtml

Supplementary table 7 The association between socioeconomic factors and appearing in specific registers among men with an indication of drug use disorder (n= 69 928) by the following registers: the Inpatient care, the Outpatient care, the Register of Medications (Medications), the Social Insurance register (Social Insurance) and the Register of convictions (Convictions), presented with odds ratios (OR) with 95 % confidence interval (CI), by age group, sex, migrant status, education level and marital status all included in the multivariate models.

		**Inpatient**	**Outpatient**	**Medication**	**Social Insurance**	**Convictions**
		OR (95 % CI)	OR (95 % CI)	OR (95 % CI)	OR (95 % CI)	OR (95 % CI)
**Age-group**	**20–29**	0.73 (0.69-0.77)	0.70 (0.67-0.74)	0.24 (0.22-0.27)	1.77 (1.52-2.07)	4.07 (3.84-4.31)
	**30–39**	0.96 (0.91-1.02)	0.98 (0.93-1.03)	0.60 (0.55-0.64)	2.08 (1.80-2.41)	2.72 (2.58-2.87)
	**40–49**	1.10 (1.04-1.16)	1.14 (1.08-1.20)	0.77 (0.72-0.83)	1.44 (1.24-1.68)	1.57 (1.49-1.65)
	**50–59**	1	1	1	1	1
	**60–65**	0.86 (0.80-0.93)	0.75 (0.69-0.80)	2.22 (2.05-2.42)	0.29 (0.21-0.42)	0.28 (0.26-0.31)
**Migrant status**	**Born abroad**	0.62 (0.59-0.65)	0.67 (0.64-0.69)	0.73 (0.69-0.79)	0.54 (0.47-0.61)	1.69 (1.61-1.78)
	**Refugee**	0.67 (0.61-0.74)	0.88 (0.81-0.96)	1.27 (1.13-1.43)	0.49 (0.37-0.66)	1.60 (1.45-1.77)
	**Sweden**	1	1	1	1	1
**Education**	**Pre-upper secondary school**	1.39 (1.34-1.44)	1.32 (1.28-1.37)	1.28 (1.22-1.35)	0.79 (0.72-0.86)	1.30 (1.25-1.35)
	**Upper secondary**	1	1	1	1	1
	**Post-secondary school**	1.02 (0.96-1.09)	1.24 (1.17-1.31)	1.13 (1.04-1.22)	1.21 (1.05-1.39)	0.48 (0.45-0.51)
	**Missing**	1.33 (1.18-1.50)	1.16 (1.04-1.29)	0.77 (0.62-0.96)	0.40 (0.25-0.62)	1.21 (1.06-1.39)
**Marital status**	**Married**	1	1	1	1	1
	**Single**	1.18 (1.12-1.25)	1.17 (1.11-1.23)	0.50 (0.47-0.54)	0.86 (0.75-0.98)	2.16 (2.05-2.28)
	**Divorced**	1.24 (1.17-1.33)	1.17 (1.10-1.25)	0.48 (0.45-0.52)	1.01(0.85-1.21)	2.09 (1.96-2.23)
	**Widow/Widower**	1.34 (1.01-1.78)	1.40 (1.08-1.84)	0.83 (0.61-1.12)	1.32 (0.58-3.02)	1.17 (0.86-1.58)

For peer review only

Supplementary table 8 The association between socioeconomic factors and appearing in specific registers among women with an indication of drug use disorder (n= 29 624) by the following registers: the Inpatient care, the Outpatient care, the Register of Medications (Medications), the Social Insurance register (Social Insurance) and the Register of convictions (Convictions), presented with odds ratios (OR) with 95 % confidence interval (CI), by age group, sex, migrant status, education level and marital status all included in the multivariate models.

		**Inpatient**	**Outpatient**	**Medication**	**Social Insurance**	**Convictions**
		OR (95 % CI)	OR (95 % CI)	OR (95 % CI)	OR (95 % CI)	OR (95 % CI)
**Age-group**	**20–29**	1.29 (1.19-1.41)	0.99 (0.91-1.08)	0.31 (0.27-0.35)	1.71 (1.37-2.14)	3.16 (2.88-3.47)
	**30–39**	1.33 (1.23-1.44)	1.24 (1.15-1.34)	0.54 (0.49-0.60)	1.88 (1.53-2.30)	2.16 (1.97-2.36)
	**40–49**	1.20 (1.11-1.29)	1.12 (1.04-1.20)	0.67 (0.61-0.73)	1.59 (1.31-1.94)	1.65 (1.52-1.80)
	**50–59**	1	1	1	1	1
	**60–65**	0.89 (0.81-0.97)	0.74 (0.68-0.81)	1.69 (1.54-1.86)	0.37 (0.26-0.53)	0.17 (0.14-0.20)
**Migrant status**	**Migrant**	1.02 (0.95-1.09)	0.98 (0.92-1.05)	0.73 (0.66-0.80)	0.69 (0.57-0.85)	0.79 (0.73-0.86)
	**Refugee**	0.99 (0.79-1.24)	0.88 (0.70-1.10)	0.78 (0.56-1.09)	0.52 (0.24-1.10)	0.77 (0.60-1.00)
	**Sweden**	1	1	1	1	1
**Education**	**Pre-upper secondary school**	1.11 (1.06-1.17)	1.08 (1.03-1.14)	1.09 (1.01-1.17)	0.62 (0.54-0.72)	1.79 (1.69-1.90)
	**Upper secondary**	1	1	1	1	1
	**Post-secondary school**	0.98 (0.92-1.05)	1.14 (1.07-1.22)	1.10 (1.01-1.20)	1.51 (1.30-1.75)	0.42 (0.38-0.47)
	**Missing**	1.17 (0.97-1.41)	1.49 (1.23-1.79)	1.12 (0.85-1.48)	0.29 (0.13-0.64)	1.27 (1.05-1.55)
**Marital status**	**Married**	1	1	1	1	1
	**Single**	1.04 (0.98-1.11)	1.19 (1.12-1.27)	0.66 (0.61-0.71)	1.01 (0.86-1.19)	2.25 (2.07-2.44)
	**Divorced**	1.27 (1.18-1.36)	1.25 (1.16-1.33)	0.55 (0.50-0.60)	0.88 (0.72-1.07)	2.19 (1.99-2.40)
	**Widow/Widower**	1.16 (0.96-1.40)	1.20 (1.00-1.45)	0.97 (0.80-1.19)	0.93 (0.49-1.77)	2.38 (1.83-3.10)

For peer review only - http://bmjopen.bmj.com/site/about/guidelines.xhtml

The RECORD statement – checklist of items, extended from the STROBE statement, that should be reported in observational studies using routinely collected health data.

	Item No.	STROBE items	Location in manuscript where items are reported	RECORD items	Location in manuscript where items are reported
**Title and abstract**					
	1	(a) Indicate the study's design with a commonly used term in the title or the abstract (b) Provide in the abstract an informative and balanced summary of what was done and what was found	Title page at page 1 and abstract at page 2.	RECORD 1.1: The type of data used should be specified in the title or abstract. When possible, the name of the databases used should be included.<br><br>RECORD 1.2: If applicable, the geographic region and timeframe within which the study took place should be reported in the title or abstract.<br><br>RECORD 1.3: If linkage between databases was conducted for the study, this should be clearly stated in the title or abstract.	Title page at page 1 and abstract at page 2.
**Introduction**					
Background rationale	2	Explain the scientific background and rationale for the investigation being reported	Page 4		
Objectives	3	State specific objectives, including any prespecified hypotheses	Page 4		
**Methods**					
Study Design	4	Present key elements of study design early in the paper	Page 4		
Setting	5	Describe the setting, locations, and relevant dates, including periods of recruitment, exposure, follow-up, and data collection	Page 5		

For peer review only

Participants	6	*(a) Cohort study* - Give the eligibility criteria, and the sources and methods of selection of participants. Describe methods of follow-up<br>*Case-control study* - Give the eligibility criteria, and the sources and methods of case ascertainment and control selection. Give the rationale for the choice of cases and controls<br>*Cross-sectional study* - Give the eligibility criteria, and the sources and methods of selection of participants<br><br>*(b) Cohort study* - For matched studies, give matching criteria and number of exposed and unexposed<br>*Case-control study* - For matched studies, give matching criteria and the number of controls per case		RECORD 6.1: The methods of study population selection (such as codes or algorithms used to identify subjects) should be listed in detail. If this is not possible, an explanation should be provided.<br><br>RECORD 6.2: Any validation studies of the codes or algorithms used to select the population should be referenced. If validation was conducted for this study and not published elsewhere, detailed methods and results should be provided.<br><br>RECORD 6.3: If the study involved linkage of databases, consider use of a flow diagram or other graphical display to demonstrate the data linkage process, including the number of individuals with linked data at each stage.	Page 5<br><br>Page 5<br><br>Page 5
Variables	7	Clearly define all outcomes, exposures, predictors, potential confounders, and effect modifiers. Give diagnostic criteria, if applicable.	Page 5-6	RECORD 7.1: A complete list of codes and algorithms used to classify exposures, outcomes, confounders, and effect modifiers should be provided. If these cannot be reported, an explanation should be provided.	Page 5-6
Data sources/ measurement	8	For each variable of interest, give sources of data and details of methods of assessment (measurement).<br>Describe comparability of assessment methods if there is more than one group	Page 5-6		

For peer review only

Bias	9	Describe any efforts to address potential sources of bias	Page 5-6		
Study size	10	Explain how the study size was arrived at	page 5		
Quantitative variables	11	Explain how quantitative variables were handled in the analyses. If applicable, describe which groupings were chosen, and why	page 5-6		
Statistical methods	12	(a) Describe all statistical methods, including those used to control for confounding<br>(b) Describe any methods used to examine subgroups and interactions<br>(c) Explain how missing data were addressed<br>(d) *Cohort study* - If applicable, explain how loss to follow-up was addressed<br>*Case-control study* - If applicable, explain how matching of cases and controls was addressed<br>*Cross-sectional study* - If applicable, describe analytical methods taking account of sampling strategy<br>(e) Describe any sensitivity analyses	Page 7		
Data access and cleaning methods		..		RECORD 12.1: Authors should describe the extent to which the investigators had access to the database population used to create the study population.	Page 5-6

For peer review only

				RECORD 12.2: Authors should provide information on the data cleaning methods used in the study.	Page 5-6
Linkage		..		RECORD 12.3: State whether the study included person-level, institutional-level, or other data linkage across two or more databases. The methods of linkage and methods of linkage quality evaluation should be provided.	Page 5-6
**Results**					
Participants	13	(a) Report the numbers of individuals at each stage of the study (*e.g.*, numbers potentially eligible, examined for eligibility, confirmed eligible, included in the study, completing follow-up, and analysed)<br>(b) Give reasons for non-participation at each stage.<br>(c) Consider use of a flow diagram	Page 7	RECORD 13.1: Describe in detail the selection of the persons included in the study (*i.e.*, study population selection) including filtering based on data quality, data availability and linkage. The selection of included persons can be described in the text and/or by means of the study flow diagram.	Page 7
Descriptive data	14	(a) Give characteristics of study participants (*e.g.*, demographic, clinical, social) and information on exposures and potential confounders<br>(b) Indicate the number of participants with missing data for each variable of interest<br>(c) *Cohort study* - summarise follow-up time (*e.g.*, average and total amount)	page 7		
Outcome data	15	*Cohort study* - Report numbers of outcome events or summary measures over time<br>*Case-control study* - Report numbers in each exposure	Page 7		

For peer review only

		category, or summary measures of exposure<br>*Cross-sectional study* - Report numbers of outcome events or summary measures			
Main results	16	(a) Give unadjusted estimates and, if applicable, confounder-adjusted estimates and their precision (e.g., 95% confidence interval). Make clear which confounders were adjusted for and why they were included<br>(b) Report category boundaries when continuous variables were categorized<br>(c) If relevant, consider translating estimates of relative risk into absolute risk for a meaningful time period	page 11-12		
Other analyses	17	Report other analyses done—e.g., analyses of subgroups and interactions, and sensitivity analyses	Page 11-12		
**Discussion**					
Key results	18	Summarise key results with reference to study objectives	Page 13		
Limitations	19	Discuss limitations of the study, taking into account sources of potential bias or imprecision. Discuss both direction and magnitude of any potential bias	Page 14	RECORD 19.1: Discuss the implications of using data that were not created or collected to answer the specific research question(s). Include discussion of misclassification bias, unmeasured confounding, missing data, and changing eligibility over time, as they pertain to the study being reported.	Page 14
Interpretation	20	Give a cautious overall interpretation of results considering objectives,	Page 13		

		limitations, multiplicity of analyses, results from similar studies, and other relevant evidence			
Generalisability	21	Discuss the generalisability (external validity) of the study results	Page 14		
**Other Information**					
Funding	22	Give the source of funding and the role of the funders for the present study and, if applicable, for the original study on which the present article is based	Page 16		
Accessibility of protocol, raw data, and programming code		..	Page 16	RECORD 22.1: Authors should provide information on how to access any supplemental information such as the study protocol, raw data, or programming code.	Page 16

*Reference: Benchimol EI, Smeeth L, Guttmann A, Harron K, Moher D, Petersen I, Sørensen HT, von Elm E, Langan SM, the RECORD Working Committee. The REporting of studies Conducted using Observational Routinely-collected health Data (RECORD) Statement. *PLoS Medicine* 2015; in press.

*Checklist is protected under Creative Commons Attribution (CC BY) license.

For peer review only

BMJ Open

Indications of alcohol- or drug use disorders in five different national registers in Sweden—a cross sectional population-based study.

Journal:	*BMJ Open*
Manuscript ID	bmjopen-2022-070744.R2
Article Type:	Original research
Date Submitted by the Author:	27-Jul-2023

Complete List of Authors:	Lundin, Andreas; Karolinska Institutet, Department of Global Public Health ; Centre for Epidemiology and Community Medicine, Region Stockholm<br>Danielsson, Anna-Karin; Karolinska Institutet, Global Public Health<br>Dalman, Christina; Karolinska Institute, Global Public Health ; Centre for Epidemiology and Community Medicine, Region Stockholm<br>Hollander, Anna-Clara; Karolinska Institutet, Department of Global Public Health

**Primary Subject Heading**:	Addiction
Secondary Subject Heading:	Epidemiology, Mental health, Public health
Keywords:	EPIDEMIOLOGY, Substance misuse < PSYCHIATRY, PUBLIC HEALTH, SOCIAL MEDICINE

SCHOLARONE™
Manuscripts

I, the Submitting Author has the right to grant and does grant on behalf of all authors of the Work (as defined in the below author licence), an exclusive licence and/or a non-exclusive licence for contributions from authors who are: i) UK Crown employees; ii) where BMJ has agreed a CC-BY licence shall apply, and/or iii) in accordance with the terms applicable for US Federal Government officers or employees acting as part of their official duties; on a worldwide, perpetual, irrevocable, royalty-free basis to BMJ Publishing Group Ltd ("BMJ") its licensees and where the relevant Journal is co-owned by BMJ to the co-owners of the Journal, to publish the Work in this journal and any other BMJ products and to exploit all rights, as set out in our licence.

The Submitting Author accepts and understands that any supply made under these terms is made by BMJ to the Submitting Author unless you are acting as an employee on behalf of your employer or a postgraduate student of an affiliated institution which is paying any applicable article publishing charge ("APC") for Open Access articles. Where the Submitting Author wishes to make the Work available on an Open Access basis (and intends to pay the relevant APC), the terms of reuse of such Open Access shall be governed by a Creative Commons licence – details of these licences and which Creative Commons licence will apply to this Work are set out in our licence referred to above.

Other than as permitted in any relevant BMJ Author's Self Archiving Policies, I confirm this Work has not been accepted for publication elsewhere, is not being considered for publication elsewhere and does not duplicate material already published. I confirm all authors consent to publication of this Work and authorise the granting of this licence.

For peer review only

Title page

The title: Indications of alcohol- or drug use disorders in five different national registers in Sweden—a cross sectional population-based study.

List the full names and institutional addresses for all authors:

Andreas Lundin[1,2]

Anna-Karin Danielsson[1]

Christina Dalman[1,2]

Anna-Clara Hollander[1]*

[1]Epidemiology of Psychiatric Conditions, Substance Use and Social Environment (EPiCSS), Department of Global Public Health, KI, Solnavägen 1E, 171 77 Stockholm, Sweden

[2]Centre for Epidemiology and Community Medicine, Region Stockholm, Stockholm, Sweden

*corresponding author: anna-clara.hollander@ki.se

For peer review only

Abstract

Objective: The purpose of this study is to examine the prevalence of indications of alcohol- or drug use disorders in five different national Swedish registers and to investigate the correlation between these registers. Furthermore, the intent is to investigate whether combining data from different registers increases the prevalence of these indications in the population, due to the identification of different demographic groups in different registers.

Design: Cross-sectional study

Setting and participants: Individuals living in Sweden aged 20–64 in 2006, n =5,453,616.

Primary outcome: National registers included the Registers of Inpatient Care, Outpatient Care, Medications, Social Insurance, and Convictions. Demographic variables were sex, age, migrant status, education, and civil status. Indications of alcohol or drug use disorders were presented as prevalence in percentage (%), correlation was examined using phi-correlation coefficients, and differences across demographic factors were studied using logistic regression.

Results: The prevalence of an indication of alcohol- or drug use disorder varied between registers, meaning that prevalence increased when all registers were considered together. The prevalence of alcohol use disorder increased by 60% and 66% among men and women, respectively, while the prevalence of drug use disorder increased by 45% and 80% among men and women, respectively, when all registers were combined, compared to only using the register with the highest prevalence. Registers contributed different indications of drug and alcohol use disorders.

Conclusions: Accurate estimates of alcohol or drug use disorders are critical for healthcare and rehabilitation. This study shows that using a single register alone underestimates the burden of disease unevenly, while combining a range of registers can provide a more accurate picture.

Keywords

alcohol use disorder; drug use disorder; substance use disorder; healthcare registers, population-based; epidemiology

Word count: 4927

For peer review only

Strengths and limitations of this study

- The study has a large sample size as it included all individuals living in Sweden aged 20–64 in 2006 (n =5,453,616).
- The five national registers included in the study are of high quality.
- A limitation of the study is that it examines the period prevalence of substance use disorder and does not take previous use into account.
- The validity of indications of alcohol- and drug use disorder depends on the validity of the different registers.

For peer review only

For peer review only - http://bmjopen.bmj.com/site/about/guidelines.xhtml

Background

Alcohol and drug use cause a substantial disease burden globally, the composition and extent of which varies between countries.[1] Globally, in 2016, 4.2% of all disability-adjusted life year (DALYs) were attributable to alcohol use, 1.3% were attributed to drug use,[1] and lost DALYs were due to risk of injury, premature death, and other negative consequences.[2] However, the risk posed by using alcohol and drugs differs between men and women, by socioeconomic circumstance, and for migrants, by country of birth.[3–5] The term 'substance use disorder' refers to both substance abuse and substance dependence, and although studies of substance use disorder are common in population surveys,[3,6] registry-based studies provide an additional way to study substance use disorders.[7–9] There are many advantages to using survey data in studies of alcohol and drug use disorders in a population, such as the ability to study different degrees of the disorder, as the participants describe their consumption and their substance-use–related problems themselves. However, disadvantages of surveys include: decreasing response rate in population surveys in general;[10] selection bias, as some socioeconomic groups are less likely to respond to surveys than others; and the fact that heavy substance use can affect memory,[11] increasing the risk of recall bias.

In Scandinavia and Finland, healthcare visits are recorded in local and national administrative registers covering the entire population. Such population-based registers have benefited psychiatric research immensely.[12] These health registers are generally considered valid,[13] but using registers collected for administrative purposes in research can give misleading results if there is a systematic underutilisation of care in specific sub-groups. In Sweden, to date, studies of substance use disorder have used the National Patient Register,[4,7–9] assuming the perspective that the most severe cases of substance use disorder will require care. This register has been an important source of knowledge about the social determinants and consequences of severe alcohol- and drug use disorders. However, it has long been recognised that—in addition to only detecting the most severe cases—these registers possibly underestimate severe cases in specific sub-groups where under-utilisation is pronounced.[14] As register-based research could be a powerful tool for population-based studies of substance use disorders, there have been attempts to expand the use of population-registers by including additional registers with possibly less severe cases, such as registers with diagnoses from general practitioners' (GP) surgeries,[15] the Crime Prevention Council's register,[16] and the Register of Prescribed and Purchased Medicine.[17] Studies using the additional registers recognise that appearing in the additional registers is not the same as being diagnosed with a substance use disorder, but acknowledge that it is a possible indication of a substance use disorder. However, despite being used in studies of the determinants and consequences of a substance use disorder, this practice has not yet been tested empirically.

The purpose of this study is to examine the prevalence of indications of alcohol- or drug use disorders in five different national Swedish registers. This was done to investigate the extent of correlation between the registers to find out whether combining registers increases the prevalence of such indications in the population, due to registers identifying different demographic groups such as different age groups, sexes, educational levels, and migrant or marital status.

Methods

Patient involvement

No patients were involved in setting the research question or the outcome measures, nor were they involved in developing plans for design or implementation of the study. No patients were asked to

For peer review only - http://bmjopen.bmj.com/site/about/guidelines.xhtml

advise on interpretation or writing up of results. However, we will disseminate the results of our research to agencies responsible for the healthcare of refugee and migrant groups in Sweden.

Design

We conducted a historical cross-sectional register-based study in several Swedish national registries. To link registers, we used the personal identity numbers that are uniquely assigned to every resident at birth or upon immigration.

Data sources

Data were extracted from a linked register database called Psychiatry Sweden (PS) held by the research group Epidemiology of Psychiatric Conditions, Substance Use, and Social Environment (EPiCSS). PS consists of several national and regional registers.

For the registers of alcohol and drug use disorders we used three registers from the National Board of Health and Welfare: the Patient Register (which actually contains two registers; one for inpatient care (henceforth called the "Inpatient Register") and one for specialised outpatient care (henceforth called the "Outpatient Register")). Data on psychiatric inpatient care has been accessible in these registers since 1973, and there has been good coverage of psychiatric outpatient data since 2006 [18]. The Register of Prescribed and Purchased Pharmaceuticals (*Läkemedelsregistret*) (accessible since 2006),[19] is a register of all medicines prescribed in healthcare and dispensed at pharmacies (henceforth called the "Register of Medications"). We also included the Swedish Social Insurance Agency's (*Försäkringskassan*) *Mikrodata* for analysis of social insurance (henceforth called the "Social Insurance" register) (accessible since 1992),[20] from which we used the variables 'sickness absence' and 'disability pension'. 'Sickness absence' refers to a temporarily-reduced work capacity due to alcohol and drug use disorders. A disability pension is a public financial support provided to the individuals who permanently leave the labour market before the age of statutory retirement, usually preceded by sick leave. From the Crime Prevention Council's register of convicted criminals (*Brottsförebyggande Rådets* (BRÅs)) Register över lagförda personer [21] (since 2000) we used the register of prosecuted convictions based on the information registered in the Swedish Public Prosecutor's Office and courts (henceforth called the "Register of Convictions").

In addition, we used registers from Statistics Sweden for demographic factors, including total population, the Longitudinal Integration Database for Health Insurance and Labour Market Studies (LISA) (since 1990),[22] and the Longitudinal Database for Integration Studies (STATIV) (since 1997).[23]

Population

The population consisted of all persons in Sweden aged 20–64 in 2006 and was identified in LISA. The lower age limit was determined by not having access to data from child and adolescent psychiatry from all over Sweden, and the higher age limit was determined as persons over 64 cannot be included in the register of Social Insurance due to the Swedish age of retirement in 2006. The population were then followed up in various registers regarding indications of alcohol and drug use disorder between 2006 and 2016.

Definitions of indication of alcohol or drug use disorder

The present study focused on use so extensive and problematic that it was captured in national registers and will henceforth be called a 'register indication' of alcohol or drug use disorder. The

For peer review only - http://bmjopen.bmj.com/site/about/guidelines.xhtml

registers primarily use the International Statistical Classification of Diseases and Related Health Problems, 10th Edition (ICD-10) [24] system of classification, where alcohol and drug use disorder has six possible criteria:

- Craving
- Loss of control
- Prioritisation of alcohol/drug consumption over other activities
- Continued intake, despite adverse effects
- Increased tolerance
- Abstinence

For a person to be diagnosed according to ICD-10, three of six criteria must have been met during the past year.[24]

Alcohol use disorder

In both the patient registers and the Social Insurance register, an indication of alcohol use disorder was an ICD-10 diagnosis of "mental disorders and behavioural disorders caused by alcohol (F10)".

In the Register of Convictions, an indication of alcohol use disorder was defined as relevant alcohol-related crimes, such as drunk driving, aggravated drunk driving, or care of intoxicated persons (Act (1951: 649) on penalties for certain traffic offenses (§ 4 drunk driving, and § 4a aggravated drunk driving, respectively). Since one of the ICD-10 criteria for alcohol use disorders is still drinking despite negative effects, it was only counted as a register indication if an individual had more than two convictions in the same calendar year.

In the Register of Medications, an indication of alcohol use disorder was defined as having been prescribed medicine to treat alcohol use disorder, including Disulfiram (ATC NO7BB01), Acamprosate (ATC NO7BB03), Naltrexone (ATC NO7BB04), or Nalmefene (ACT NO7BB05).

Drug use disorder

In the Patient registers and the Social Insurance register, an indication of drug use disorder was an ICD-10 diagnosis of chapters F11–F14, F16, F18–F19, which are mental disorders and behavioural disorders caused by:

- opioids (F11)
- cannabinoids (F12)
- sedatives or hypnotics (F13)
- cocaine (F14)
- hallucinogens (F16)
- volatile solvents (F18)
- psychoactive substances (F19)

In the Register of Convictions, an indication of drug use disorder was a relevant drug-related crime (Narcotics Penal Code (1968: 64), and the Act (1991: 1969) on the prohibition of certain substances such as possession, use of, or other engagement with drugs. As one of the ICD-10 criteria for drug use disorder is continued use despite negative effects, it only counted as a register indication when a person had more than two convictions during the same calendar year.

For peer review only

In the Register of Medications, an indication of drug use disorder was having been prescribed and dispensed Buprenorphine N07BC01, Methadone N07BC02, Levomethadone N07BC05 or Buprenorphine, or combinations N07BC51 (only drugs for opiate dependence were available).

Co-variates

- Sex was defined as sex assigned at birth: male or female.
- Age was categorised in age groups: 20–29, 30–39, 40–49, 50–59, and 60–64.
- Migration status was grouped into those born in Sweden, migrants, and refugees; 'refugees' were defined as having received a residence permit for refugee reasons according to the Swedish Migration Agency.
- Education level was grouped according to Swedish education nomenclature as pre-secondary, upper secondary, post-secondary, and unknown.
- Marital status was grouped as married (including registered partnership), divorced (including deregistered partnership), widow/widower, and unmarried.

Statistical analysis

Alcohol and drug use disorder in the registers were presented as cumulative period prevalence and reported in percentages. Correlation—i.e., the extent to which those with an indication of substance use disorder in one register appear in other registers—was examined with phi-correlation for dichotomous variables. Phi correlation is interpreted as a scale where the correlation coefficient is between 1 and –1, where 1 means maximum positive correlation, 0 is no correlation at all, and –1 is maximum negative correlation. Relationships between the demographic variables (age, sex, migrant status, level of education, marital status) and indication of alcohol or drug use disorder in different registers were examined using logistic regression and presented as odds ratios (OR) and 95% confidence intervals (CI). All regression models were multivariable models including the variables age, sex, migrant status, education level, and marital status.

Results

The population consisted of all individuals in Sweden aged 20–64 in 2006, for a total of 5.45 million individuals, and is described in Table 1.

Table 1. Characterisation of the population in 2006 in number (n) and percent (%).

		**n (%)**
**Characteristics**	**Total**	5,453,616 (100)
**Sex**	Male	2,781,344 (51)
	Female	2,672,272 (49)
**Migration status**	Born in Sweden	4,581,037 (84)
	Born abroad	818,042 (15)
	Refugee	109,072 (2)
**Education**	Pre-upper secondary school	872,578 (16)
	Upper secondary	2,617,736 (48)
	Post-secondary school	1,854,229 (34)
	Missing	109,072 (2)
**Age group**	20–29	1,090,723 (20)
	30–39	1,254,332 (23)
	40–49	1,254,332 (23)
	50–59	1,199,796 (22)
	60–64	708,970 (13)

For peer review only - http://bmjopen.bmj.com/site/about/guidelines.xhtml

**Marital status**	Married	2,345,055 (43)
	Unmarried	2,345,055 (43)
	Divorced	654,434 (12)
	Widow widower	54,536 (1)

Table 2 shows the prevalence of an indication of alcohol use disorder by sex and age in the population. The prevalence of individuals with a register indication for alcohol use disorder differed between registers. In total, 2.0% of the women and 4.2% of the men appeared in one of these registers. Due to the doubled prevalence of an indication among men, compared with women, Table 2 is also displayed by men and women separately (see supplementary files; supplementary Table 1 and supplementary Table 2). Those aged 50–59 years had the highest prevalence in all registers except the Register of Convictions. The highest prevalence for both men and women was found in the Register of Medications (men 2.6%, women 1.2%), followed by the Inpatient and Outpatient Register and the Social Insurance Register. The lowest prevalence was found in the Register of Convictions. Comparing all registers combined with the register with the highest prevalence increased total prevalence by 60% for men and by 66% for women. Prevalence in the different age groups increased correspondingly from 24% to 67%, with the largest increase among people aged 30–39 years. Table 2 also shows the correlation between the registers—i.e., the extent to which persons with an indication of alcohol use disorder in one register appear in other registers—and the unique contribution in percent of each register to a register indication. Register indication in the In- and Outpatient Registers and the Register of Medications had the highest correlation; these three registers contributed the largest proportion of unique indications. The correlations between indications in other registers were strongest if a person appeared in the Register of Medications, and lowest if the person appeared in The Register of Convictions. The Social Insurance Register and Register of Convictions had the smallest unique contributions.

For peer review only

For peer review only - http://bmjopen.bmj.com/site/about/guidelines.xhtml

Table 2. Indication of alcohol use disorder in the total population. Number (n) and percent (%) with register indication by age group, migrant status, education, marital status, and phi (the correlation coefficient) between the registers, and the unique contribution of the specific register to all registers combined (any registers) in percent (%) for the following registers: Inpatient care, Outpatient care, the Register of Medications (Medications), the Social Insurance Register (Social Insurance), the Register of Convictions (Convictions), and Any register.

	Register	In-patient	Out-patient	Medications	Social Insurance	Convictions	Any register
**n (%)**		66,940 (100)	96,279 (100)	103,199 (100)	9,323 (100)	1,837 (100)	167,178 (100)
**Sex**	Men	1131 (1.69)	2282 (2.37)	2673 (2.59)	21 (0.23)	110 (0.06)	6938 (4.15)
	Women	502 (0.75)	1107 (1.15)	1207 (1.17)	10 (0.11)	18 (0.01)	3260 (1.95)
**Age**	20–29	609 (0.91)	1521 (1.58)	1135 (1.1)	6 (0.06)	73 (0.04)	4330 (2.59)
	30–39	522 (0.78)	1281 (1.33)	1393 (1.35)	11 (0.12)	55 (0.03)	3762 (2.25)
	40–49	850 (1.27)	1839 (1.91)	2239 (2.17)	22 (0.24)	73 (0.04)	5383 (3.22)
	50–59	1111 (1.66)	2166 (2.25)	2652 (2.57)	27 (0.29)	73 (0.04)	6537 (3.91)
	60–64	917 (1.37)	1608 (1.67)	1950 (1.89)	9 (0.1)	37 (0.02)	5233 (3.13)
**Migrant status**	Born in Sweden	837 (1.25)	1733 (1.8)	2064 (2)	17 (0.18)	55 (0.03)	5283 (3.16)
	Born abroad	777 (1.16)	1617 (1.68)	1465 (1.42)	11 (0.12)	73 (0.04)	4480 (2.68)
	Refugee	348 (0.52)	857 (0.89)	640 (0.62)	4 (0.04)	37 (0.02)	2407 (1.44)
**Education**	Education pre-upper	1573 (2.35)	2937 (3.05)	3127 (3.03)	23 (0.25)	165 (0.09)	8743 (5.23)
	Upper second	910 (1.36)	1849 (1.92)	2147 (2.08)	20 (0.21)	55 (0.03)	5617 (3.36)
	Post-second	355 (0.53)	924 (0.96)	1166 (1.13)	8 (0.09)	18 (0.01)	2792 (1.67)
	Miss	562 (0.84)	1069 (1.11)	898 (0.87)	3 (0.03)	73 (0.04)	3126 (1.87)
**Marital status**	Married	415 (0.62)	924 (0.96)	1342 (1.3)	9 (0.1)	18 (0.01)	3126 (1.87)
	Unmarried	951 (1.42)	2012 (2.09)	2064 (2)	18 (0.19)	92 (0.05)	5818 (3.48)
	Divorced	1801 (2.69)	3331 (3.46)	3725 (3.61)	34 (0.37)	110 (0.06)	9730 (5.82)
	Widow(er)	1038 (1.55)	1858 (1.93)	2219 (2.15)	14 (0.15)	55 (0.03)	5901 (3.53)
**Correlation coefficient**	Inpatient	x	0.54	0.41	0.25	0.04	0.63
	Outpatient		x	0.49	0.22	0.04	0.75
	Medications			x	0.22	0.04	0.78
	Social Insurance				x	0.02	0.23
	Convictions					x	0.10
	Any registers						x
**Unique contribution to Any register**		9.01	17.66	27.13	0.48	0.60	x

For peer review only - http://bmjopen.bmj.com/site/about/guidelines.xhtml

Individuals with an indication of alcohol use disorder were n =167,175 and differed by age, sex, migrant status, education level, and marital status (see Table 3). Due to this doubled prevalence among men, Table 3 is also displayed by men and women separately (see supplementary file, supplementary Table 3 and supplementary Table 4). Women with an indication of alcohol use disorder appeared more often in the Outpatient register (8% more likely than men), but had a lower likelihood of being found in the Inpatient register (4% less likely than men), Register of Medications (12% less likely than men) and Register of Convictions (74% less likely than men) (see Table 3). Both for the total population with an indication of alcohol use disorders, and for men and women separately, the likelihood of having an indication in the Inpatient Register increased with age and the likelihood of an indication in the Register of Convictions decreased with age. Men with an indication of alcohol use disorder aged 20–29 or 40–49 years were more likely to be found in the Outpatient Register compared to those in other age-groups, but this pattern was not seen in women. Both for the total population with an indication of alcohol use disorders, and for men and women separately, those aged 40–49 and 50–59 years appeared more often in the Register of Medications (Table 3; see supplementary file, supplementary Table 3 and supplementary Table 4). Migrants with an indication of alcohol use disorder were more likely to appear in the Inpatient and Outpatient Registers, and they were also more likely to have an indication in the Register of Convictions, compared with those born in Sweden, with little difference between men and women. Persons born in Sweden with an indication of alcohol use disorder, on the other hand, were more likely to appear in the Register of Medications and in the Social Insurance register compared with migrants (Table 3). People with an indication of alcohol use disorder and those with post-secondary education appeared more often in the Register of Medications and the Outpatient Register compared with other education levels. However, among those with an indication of alcohol use disorder and pre-secondary education, it was more common to appear in the Register of Convictions and in the Inpatient Register. People with an indication of alcohol use disorder who were married were more likely to be found in in the Register of Medications than individuals who were unmarried, divorced, and widows and widowers (see Table 3).

For peer review only

For peer review only - http://bmjopen.bmj.com/site/about/guidelines.xhtml

Table 3. The association between socioeconomic factors and appearing in specific registers among those with an indication of alcohol use disorder (n =167,175) by the following registers: Inpatient care, the Outpatient care, the Register of Medications (Medications), the Social Insurance Register (Social Insurance) and the Register of Convictions (Convictions), presented with odds ratios (OR) with 95% confidence interval (CI), by age group, sex, migrant status, education level, and marital status included in the multivariable models.

		**Inpatient**	**Outpatient**	**Medications**	**Social Insurance**	**Convictions**
		OR (95% CI)	OR (95% CI)	OR (95% CI)	OR (95% CI)	OR (95% CI)
**Age-group**	**20–29**	0.62 (0.60–0.65)	1.07 (1.03–1.11)	0.46 (0.45–0.48)	0.30 (0.27–0.34)	1.63 (1.38–1.93)
	**30–39**	0.67 (0.65–0.69)	1.02 (0.99–1.06)	0.86 (0.83–0.89)	0.70 (0.65–0.75)	1.53 (1.33–1.78)
	**40–49**	0.87 (0.85–0.90)	1.07 (1.04–1.10)	1.11 (1.08–1.15)	1.01 (0.96–1.07)	1.34 (1.17–1.52)
	**50–59**	1	1	1	1	1
	**60–64**	1.09 (1.06–1.13)	0.88 (0.86–0.91)	0.74 (0.72–0.76)	0.43 (0.40–0.46)	0.64 (0.54–0.75)
**Sex**	**Female**	0.96 (0.94–0.98)	1.08 (1.06–1.10)	0.88 (0.86–0.90)	0.99 (0.94–1.03)	0.26 (0.22–0.30)
	**Male**	1	1	1	1	1
**Migrant status**	**Migrant**	1.15 (1.12–1.19)	1.29 (1.25–1.33)	0.63 (0.61–0.65)	0.77 (0.72–0.83)	1.27 (1.12–1.45)
	**Refugee**	0.93 (0.83–1.04)	1.25 (1.12–1.40)	0.39 (0.35–0.44)	0.46 (0.34–0.64)	1.28 (0.84–1.97)
	**Sweden**	1	1	1	1	1
**Education**	**Pre-upper secondary school**	1.17 (1.15–1.20)	1.04 (1.01–1.06)	0.90 (0.88–0.92)	0.80 (0.76–0.84)	1.58 (1.43–1.75)
	**Upper secondary**	1	1	1	1	1
	**Post-secondary school**	0.69 (0.67–0.71)	1.06 (1.03–1.09)	1.23 (1.19–1.26)	0.87 (0.82–0.92)	0.59 (0.49–0.70)
	**Missing**	1.30 (1.17–1.43)	0.98 (0.88–1.08)	0.81 (0.73–0.89)	0.40 (0.28–0.58)	1.82 (1.31–2.52)
**Marital status**	**Single**	1.56 (1.52–1.60)	1.39 (1.35–1.43)	0.64 (0.62–0.66)	1.11 (1.05–1.18)	1.29 (1.13–1.48)
	**Married**	1	1	1	1	1
	**Divorced**	1.62 (1.58–1.67)	1.39 (1.35–1.42)	0.73 (0.71–0.75)	1.25 (1.18–1.32)	1.56 (1.34–1.81)
	**Widow/Widower**	1.41 (1.29–1.53)	1.18 (1.09–1.28)	0.79 (0.73–0.86)	1.06 (0.87–1.30)	1.69 (1.06–2.71)

For peer review only

For peer review only - http://bmjopen.bmj.com/site/about/guidelines.xhtml

The prevalence of individuals with an indication of drug use disorder in any of the registers was 1.1% among women and 2.5% among men. Those aged 20–29 years had the highest prevalence in all registers, except in the Register of medications (Table 4). The prevalence of an indication of drug use disorder was consistently higher for men than women. Due to the more than doubled prevalence among men, Table 4 is displayed for men and women separately (see supplementary file; supplementary Table 5 and supplementary Table 6). For all registers, except in the Register of Medications, prevalence decreased with age. For men, the highest prevalence was found in the Register of Convictions (1.7%), followed by the Outpatient and Inpatient registers, and in the Register of Medications. For women, the highest prevalence was found in the Outpatient Register (0.6%), followed by the Inpatient Register, the Register of Convictions, and in the Register of Medications. Both men and women had the lowest prevalence in the Social Insurance Register. For drug use disorder, the prevalence increased by 45% for men and by 80% for women if all registers were combined, instead of the register with the highest prevalence alone. The prevalence in different age groups increased correspondingly by between 30% to 109%, with the largest increase among people in the 50–59 age group.

There was a correlation between having an indication of a drug use disorder between the registers. The correlation was highest between the Inpatient and Outpatient registers (0.56). The correlation between having an indication in another register was strongest if the individual appeared in the Register of Convictions (0.76) and lowest if they appeared in the Social Insurance Register (0.19). The register contributing the highest proportion of individual cases for men was the Register of Convictions, and Outpatient care registers contributed the highest proportion of individual cases for women. The register contributing the smallest proportion of cases was the Register of Social Insurance, for both sexes.

For peer review only

For peer review only - http://bmjopen.bmj.com/site/about/guidelines.xhtml

Table 4. Indication of alcohol use disorder in the total population. Number (n) and percent (%) with register indication by age group, migrant status, education, marital status, and phi (the correlation coefficient) between registers and the unique contribution of each specific register to all registers combined (Any registers) in percent (%) for the following registers: Inpatient care, Outpatient care, the Register of Medications (Medications), the Social Insurance Register (Social Insurance), the Register of Convictions (Convictions), and Any register.

		Inpatient	Outpatient	Medications	Social Insurance	Convictions	Any registers
**Total number**	n (%)	32,175 (100)	46,123 (100)	13,293,175 (100)	3,559 (100)	57,211 (100)	99,552 (100)
**Sex**	Men	238,175 (0.74)	494,175 (1.07)	39,175 (0.29)	3175 (0.09)	995,175 (1.74)	2,519,175 (2.53)
	Women	142,175 (0.44)	281,175 (0.61)	25,175 (0.19)	1175 (0.04)	195,175 (0.34)	1,095,175 (1.1)
**Age**	20–29	389,175 (1.21)	789,175 (1.71)	28,175 (0.21)	6175 (0.16)	183,1175 (3.2)	4,131,175 (4.15)
	30–39	238,175 (0.74)	498,175 (1.08)	33,175 (0.25)	4175 (0.1)	841,175 (1.47)	2,220,175 (2.23)
	40–49	196,175 (0.61)	406,175 (0.88)	33,175 (0.25)	2175 (0.06)	515,175 (0.9)	1,722,175 (1.73)
	50–59	148,175 (0.46)	309,175 (0.67)	35,175 (0.26)	1175 (0.04)	326,175 (0.57)	1,394,175 (1.4)
	60–64	68,175 (0.21)	134,175 (0.29)	32,175 (0.24)	0175 (0.01)	51,175 (0.09)	687,175 (0.69)
**Migrant status**	Born in Sweden	190,175 (0.59)	383,175 (0.83)	32,175 (0.24)	2175 (0.07)	555,175 (0.97)	1,732,175 (1.74)
	Born abroad	187,175 (0.58)	401,175 (0.87)	31,175 (0.23)	2175 (0.05)	801,175 (1.4)	2,170,175 (2.18)
	Refugee	248,175 (0.77)	600,175 (1.3)	60,175 (0.45)	2175 (0.06)	1,173,175 (2.05)	2,997,175 (3.01)
**Education**	Education pre-upper	467,175 (1.45)	918,175 (1.99)	73,175 (0.55)	4175 (0.12)	1,562,175 (2.73)	4,102,175 (4.12)
	Upper second	187,175 (0.58)	387,175 (0.84)	32,175 (0.24)	2175 (0.07)	612,175 (1.07)	1,901,175 (1.91)
	Post-second	61,175 (0.19)	138,175 (0.3)	15,175 (0.11)	1175 (0.03)	103,175 (0.18)	587,175 (0.59)
	Miss	219,175 (0.68)	438,175 (0.95)	23,175 (0.17)	1175 (0.03)	887,175 (1.55)	2,200,175 (2.21)
**Marital status**	Married	68,175 (0.21)	138,175 (0.3)	23,175 (0.17)	1175 (0.02)	120,175 (0.21)	677,175 (0.68)
	Unmarried	293,175 (0.91)	604,175 (1.31)	37,175 (0.28)	4175 (0.11)	1,093,175 (1.91)	2,837,175 (2.85)
	Divorced	270,175 (0.84)	540,175 (1.17)	49,175 (0.37)	2175 (0.07)	595,175 (1.04)	2,349,175 (2.36)
	Widow(er)	119,175 (0.37)	240,175 (0.52)	43,175 (0.32)	1175 (0.02)	132,175 (0.23)	1,015,175 (1.02)
**Correlation coefficient**	Inpatient	x	0.56	0.23	0.20	0.33	0.56
	Outpatient		x	0.27	0.21	0.36	0.68
	Medications			x	0.10	0.15	0.36
	Social Insurance				x	0.12	0.19
	Convictions					x	0.76
	Any registers						x
**Unique contribution to Any register**		7.99	15.99	6.31	0.51	35.88	x

For peer review only

For peer review only - http://bmjopen.bmj.com/site/about/guidelines.xhtml

Individuals who were found to have an indication of drug use disorder (n =99,529) differed by age, sex, migrant status, education level, and marital status (see Table 5). Due to the more than doubled prevalence among men, Table 5 is also displayed by men and women separately (see supplementary file; supplementary Table 7 and supplementary Table 8). In terms of sex differences among those with an indication of drug use disorder, women were more likely to appear in the Outpatient and Inpatient registers as well as in the Register of Medications compared to men, while men were more likely to appear in the Register of Convictions. Those aged 20–29 years with an indication of drug use disorder were more likely to appear in the Register of Convictions. Persons with an indication of drug use disorder aged 30–49 years were more likely than other groups to be found both in the Register of Convictions (together with those aged 20-29) and to have an indication in the Social Insurance Register. Individuals in the oldest age group (60–64 years) with an indication of drug use disorder were more likely to appear in the Register of Medications (see Table 5). Male migrants with an indication of drug use disorder were more likely to appear in the Register of Convictions compared to individuals born in Sweden, but this was not seen among women (see supplementary file; supplementary Table 7 and supplementary Table 8). Those with a pre-secondary education were more likely to appear in all registers, except the Social Insurance Register, compared to those with a higher education. Unmarried and divorced individuals with an indication of drug use disorder were more likely to appear in the Inpatient register and in the Register of Convictions compared with married individuals. Married individuals with an indication of drug use disorders appeared more often than other marital statuses in the Register of Medications.

For peer review only

Table 5. The association between socioeconomic factors and appearing in specific registers among those with an indication of drug use disorder n =99,529 by the following registers: Inpatient care, Outpatient care, the Register of Medications (Medications), the Social Insurance Register (Social Insurance), and the Register of Convictions (Convictions) and any register (all registers combined) presented with odds ratios (OR) with 95 % confidence interval (CI), including age group, sex, migrant status, education level, and marital status in the in the multivariable models.

		**Inpatient**	**Outpatient**	**Medications**	**Social Insurance**	**Convictions**
		OR (95% CI)	OR (95% CI)	OR (95% CI)	OR (95% CI)	OR (95% CI)
**Age-group**	**20–29**	0.86 (0.82–0.90)	0.77 (0.74–0.80)	0.26 (0.24–0.28)	1.74 (1.53–1.97)	3.82 (3.64–4.01)
	**30–39**	1.07 (1.02–1.12)	1.05 (1.01–1.09)	0.57 (0.54–0.61)	2.00 (1.78–2.26)	2.57 (2.46–2.69)
	**40–49**	1.13 (1.08–1.18)	1.13 (1.09–1.18)	0.73 (0.69–0.77)	1.49 (1.32–1.68)	1.61 (1.54–1.69)
	**50–59**	1	1	1	1	1
	**60–64**	0.86 (0.81–0.91)	0.74 (0.70–0.78)	1.97 (1.85–2.09)	0.33 (0.26–0.42)	0.25 (0.23–0.27)
**Sex**	**Female**	1.61 (1.56–1.66)	1.69 (1.64–1.74)	1.27 (1.22–1.32)	1.07 (1.00–1.16)	0.22 (0.22–0.23)
	**Male**	1	1	1	1	1
**Migrant status**	**Born abroad**	0.72 (0.69–0.75)	0.74 (0.71–0.76)	0.73 (0.69–0.77)	0.58 (0.52–0.64)	1.38 (1.32–1.43)
	**Refugee**	0.73 (0.67–0.80)	0.90 (0.83–0.97)	1.20 (1.08–1.35)	0.50 (0.38–0.65)	1.40 (1.28–1.54)
	**Sweden**	1	1	1	1	1
**Education**	**Pre-upper secondary school**	1.30 (1.26–1.34)	1.25 (1.22–1.29)	1.21 (1.16–1.27)	0.74 (0.69–0.80)	1.44 (1.39–1.49)
	**Upper secondary**	1	1	1	1	1
	**Post-secondary school**	1.01 (0.96–1.05)	1.20 (1.15–1.26)	1.13 (1.06–1.20)	1.36 (1.23–1.50)	0.46 (0.44–0.49)
	**Missing**	1.27 (1.15–1.41)	1.23 (1.12–1.34)	0.89 (0.75–1.05)	0.36 (0.25–0.54)	1.22 (1.09–1.36)
**Marital status**	**Single**	1.14 (1.09–1.19)	1.19 (1.14–1.24)	0.56 (0.53–0.59)	0.91 (0.82–1.01)	2.15 (2.05–2.25)
	**Married**	1	1	1	1	1
	**Divorced**	1.25 (1.19–1.31)	1.20 (1.15–1.26)	0.51 (0.48–0.54)	0.95 (0.83–1.08)	2.12 (2.01–2.23)
	**Widow/ Widower**	1.17 (1.00–1.37)	1.21 (1.04–1.41)	0.87 (0.74–1.03)	1.05 (0.63–1.74)	1.72 (1.41–2.10)

For peer review only

Discussion

The study demonstrates that the proportion of individuals with an indication of alcohol or drug use disorder differs between registers, but that there is a correlation between the registers. This means that those with a register indication of substance use in one register sometimes—but not always—appear in one or more other registers, thus the proportion increases when registers are added together. The proportion of the population with an indication of alcohol or drug use disorders increases by between 45%–80 % when compared to only the register showing the highest prevalence. For alcohol use disorder, the three healthcare registers (i.e., the Inpatient and the Outpatient registers and the Register of Medications) had the highest correlations, however, despite this correlation the three healthcare registers also had the largest proportions of unique alcohol use disorder indications. The registers for Social Insurance and Convictions contribute less than one percent (1%) each. Still, if the Social Insurance Register is not included, this could bias the results towards missing indications of alcohol use disorders among individuals who are single or divorced. If the Register of Convictions is excluded, this could bias the results towards missing indications of alcohol use disorder among younger individuals (up to 49), those not born in Sweden, those without a higher education, and those currently unmarried. For drug use disorder, the highest correlation with other registers was when an individual appeared in the Outpatient register or the Register of Convictions. These two registers also contribute the most independent indications. The Social Insurance Register contributes less than one percent (1%), however, if excluded, this could bias the results towards missing indications of drug use disorders among individualst younger than 49 years and those with a higher education.

Findings in context

In this study, using all registers showed the proportion of the population with an indication of alcohol use disorder as 4.2% for men and 2.0% for women. This can be compared to a cross-sectional study performed by Andréasson et al. based on survey data from randomly-selected 19 to 70-year-olds from 12 Swedish municipalities,[3] where researchers found that the proportion classified as having an alcohol use disorder was 4.9% for men and 3.2% for women. The same study also found that an average of 4% of the population in Sweden meet at least three criteria for alcohol use disorder, while among 19 to 25-year-olds the prevalence was higher (around 11 %), and in this group there was no statistical difference between women and men; a difference which was found in all other age groups.[3] These findings were also in line with the results from the 2017 survey "*Vanor och konsekvenser*" [in English "Habits and consequences"].[6] The difference in prevalence between the two other studies and our study, especially in the case of women, indicates that an individual may estimate their alcohol consumption as high without seeking care. The study by Andréasson et al. also shows that the youngest group (19–25 years) had the highest prevalence of alcohol use disorder (around 1 %), while our study shows that the age group 50–59 years has the highest prevalence (3.9%). The registers (except the Register of Convictions) are largely based on people that have been utilising health care, so the difference between the previous survey-based studies and the present study may be due to lower care-seeking for substance use disorder among younger age groups compared to older age groups. It could also be due to the fact that the same alcohol intake could have different consequences in different age groups. This would mean that a younger person drinking a hazardous amount might perceive this as not harmful, whereas an older person drinking the same amount may experience hazardous consequences more directly and is therefore more likely to seek care. It was only in the register unrelated to care seeking, the Register of Convictions, where the youngest age group had a higher likelihood compared to the older age groups.

For peer review only - http://bmjopen.bmj.com/site/about/guidelines.xhtml

We found that 2.5% of men and 1.1% of women had an indication of drug use disorder. This can be compared with the results from Central Association for Alcohol and Drug Information (CAN) survey,[6] where drug dependence according to the Diagnostic and Statistical Manual of Mental Disorders 5 (DSM-5) [25] amounted to 1.9% among men and 1.6% among women. The prevalence among men was therefore higher according to register data than in the survey-based study, while the opposite applied to women. In the present study, the Register of Convictions was central to an indication of drug use disorders, and it was more common for men than for women to occur in this register. Among men, the highest prevalence of an indication of drug use disorder was captured by the Register of Convictions (1.7%), followed by the Outpatient register (1.1%). Among women, most were captured in the Outpatient and Inpatient registers (0.6% and 0.4%, respectively), followed by the Register of Convictions (0.3%). The correlation was strongest if an individual appeared in the Register of Convictions, but this register also captured cases that were not in other registers. The vast coverage of this register was expected, as both drug possession and drug use are illegal in Sweden.

The prevalence of indications of alcohol and drug use disorder differ between registers. For example, drug use disorder was captured by the Register of Convictions to a stronger extent than alcohol use disorder, which might be explained by the fact that alcohol is legal while drugs are illegal. This legal difference not only affects the availability of these substances and thus their use, their problematic use, and the need for care, but also which register identifies said use. In the case of alcohol use disorder, the Register of Medications had the highest prevalence among both men and women. In addition, the Register of Medications showed a different pattern than the Outpatient and Inpatient care registers. Among those with an indication of alcohol use disorder who were married, those born in Sweden and those with a post-secondary education had a higher likelihood to be found in this register, but were less likely to be found in the Outpatient and Inpatient registers. The finding that medications targeting alcohol use disorder are prescribed to a stronger extent among people born in Sweden and among people with higher education, compared with migrants and those with a shorter education, is in line with previous studies.[26]

Strengths and limitations

This study examines the proportion of individuals with an indication of alcohol or drug use disorder during a specific period (period prevalence) without concern of previous or repeated use of healthcare or the social insurance system. Individuals found in multiple registers are likely to have more severe alcohol and drug use disorders. Although adding all the registers together increases the prevalence of indications of substance use disorders, this study probably underestimates the prevalence of early stages of substance use disorder, as those at this stage possibly are less likely to seek care and might be more careful in terms of, for instance, drunk driving. Also, we have not included diagnoses from primary care, therefore, some individuals seeking care at an early stage may not be detected. However, as we include the register of Medications where all prescribed medications are registered, including medications prescribed in primary care, and this suggested the underestimation of patients with substance use disorder in primary care is limited to patients who have not been prescribed medication, or have not been purchasing medications prescribed by a GP, and have not been referred to outpatient care. Another limitation is the age span of 20–64 as both alcohol and drug use disorders are common among adolescents and young adults and among the elderly.

A valid substance use diagnosis can be described as a diagnosis that meets the ICD-10 criteria. The validity of indications of alcohol and drug use disorder in this study depends on the validity of the different registers. The diagnoses in the Inpatient and Outpatient registers were made by physicians and can therefore be considered valid.[13] Being offered sickness or activity compensation by the

For peer review only - http://bmjopen.bmj.com/site/about/guidelines.xhtml

Swedish Social Insurance Agency is preceded by a process in which physicians are involved, and although there are some differences in insurance medical practice over the country, these indications can also be considered valid. With regard to the Register of Medications, none of the medications used to treat alcohol use disorder included in the study have other uses according to the Swedish Pharmacopeia Information guide,[27] and all were prescribed by physicians, and can therefore be considered valid. Some of the medications included as a marker for drug use disorder in the study can, in some special cases, be used for pain relief, which reduces the validity. With regard to the Register of Convictions, this differs from the other registers in that it only measures one aspect of substance use disorder, namely the ICD-10 criterion of continued drinking or use despite negative effects, and is therefore only a proxy variable; i.e., a variable that replaces another variable when it cannot be directly observed. It can, accordingly, be considered to have the lowest validity. Only cases when a person had more than two convictions for relevant alcohol- or drug-related crime during a calendar year were counted as an indication of substance use disorder. More individuals with alcohol or drug use disorder would have been identified from the Register of Convictions if the threshold of number of convictions during a calendar had been lower; however this practice could also have increased the number of false positives and was not graded according to the ICD-10 criteria of substance use disorder.[24] Having three convictions for severe forms of alcohol-related crime is relatively rare, while for aggravated drunk driving there is a suspension period—i.e., a time when the driving license is revoked—which typically amounts to 12 months. Regarding the indication of drug use disorder in the Register of Convictions, it is not certain that possession or sale of drugs involves either alcohol use or drug use disorder, although studies show that people who sell drugs usually also use drugs.[28,29] There are other variables that could possibly have also been included as an indication of substance use disorder, such as intoxication (from the Inpatient or Outpatient registers) where more than two occurrences could be an indicator of possible SUD. With regard to education, this variable is more often missing among migrants compared to individuals born in Sweden, and this could bias the results when it comes to education.

Conclusion

The conclusion of this study is that the use of a combination of relevant population-based registers improves the ability to describe the prevalence of alcohol and drug use disorder in the population. Another effect of combining multiple registers is that indications of alcohol or drug use disorder among younger people and migrants, as well as among married individuals and those with a higher education, increased in comparison to only using one register. The registers contribute differently to indications of alcohol and drug use disorder. For instance, the Register of Convictions contributes less than one percent (1%) of the unique indications of alcohol use disorder but contribute more than 35% of the unique indications of drug use disorders. The study increases knowledge of how the use of different administrative registers creates a more accurate picture of the prevalence and differences in substance use disorder between different demographic groups in the population, although it is important to keep in mind the validity of the different registers.

For peer review only

Declarations

Ethics approval and consent to participate

The project and the register linking were approved by the Regional Ethical Review Board in Stockholm (dnr. 2016/987-31).

Consent for publication

Not applicable

Availability of data and materials

Data is available on request for any interested researchers to allow replication of results through the Swedish National Data Service, provided all ethical and legal requirements are met. Detailed information on data application can be found at https://www.registerforskning.se/en/.

Competing interests

The authors declare that they have no competing interests

Funding

ACH is supported by the Swedish council for Health, Working Life, and Welfare (FORTE, by Swedish acronym) (grant numbers 2016-00870). CD is supported by the Swedish Research Council (grant number 5232010-1052). This work was partially funded by the Public Health Agency of Sweden.

Authors' contributions

AL, ACH, and CD conceived the study. AL and ACH designed the study. ACH obtained funding from FORTE and the Public Health Agency of Sweden. CD acquired the cohort data. AL prepared the data. AL wrote the study protocol, conducted the statistical analyses, and drafted the data tables. AL and ACH interpreted the statistical analyses. AKD and ACH wrote the manuscript. All authors critically revised the paper for important intellectual content and approved the final version.

Acknowledgements

Parts of the results of this study has been published with other research questions in a report published by the Public Health Agency of Sweden in Swedish (https://www.folkhalsomyndigheten.se/publicerat-material/publikationsarkiv/a/alkohol--och-narkotikaberoende/).

References

1. Degenhardt L, Charlson F, Ferrari A, Santomauro D, Erskine H, Mantilla-Herrara A, Whiteford H, Leung J, Naghavi M, Griswold M *et al*: **The global burden of disease attributable to alcohol and drug use in 195 countries and territories, 1990-2016: a systematic analysis for the Global Burden of Disease Study 2016**. *Lancet Psychiatry* 2018, **5**(12):987-1012.
2. Agardh E, Boman U, Allebeck P: **Alkohol, narkotika och tobaksrökning ger stor del av sjukdomsbördan - Utvecklingen i Sverige 1990–2010 kartlagd utifrån DALY-metoden**. *Läkartidningen* 2015, **2015;112:C4TH**.
3. Andreasson S, Danielsson AK, Hallgren M: **Severity of alcohol dependence in the Swedish adult population: association with consumption and social factors**. *Alcohol* 2013, **47**(1):21-25.
4. Kendler KS, Ohlsson H, Sundquist K, Sundquist J: **Prediction of drug abuse recurrence: a Swedish National Study**. *Psychological Medicine* 2018, **48**(8):1367-1374.

For peer review only - http://bmjopen.bmj.com/site/about/guidelines.xhtml

5. Syden L, Sidorchuk A, Makela P, Landberg J: **The contribution of alcohol use and other behavioural, material and social factors to socio-economic differences in alcohol-related disorders in a Swedish cohort**. *Addiction* 2017, **112**(11):1920-1930.
6. Landberg J, Ramstedt M, Sundin E: **Socioekonomiska skillnader i beroende och utsatthet för andras användning av alkohol, narkotika och tobak**. In: *CAN-rapport.* Edited by narkotikaupplysning Cfa-o; 2018: 91 sidor.
7. Hjern A, Allebeck P: **Alcohol-related disorders in first- and second-generation immigrants in Sweden: a national cohort study**. *Addiction* 2004, **99**(2):229-236.
8. Manhica H, Gauffin K, Almquist YB, Rostila M, Berg L, de Cortazar ARG, Hjern A: **Hospital admissions due to alcohol related disorders among young adult refugees who arrived in Sweden as teenagers - a national cohort study**. *Bmc Public Health* 2017, **17**.
9. Harris S, Dykxhoorn J, Hollander A-C, Dalman C, Kirkbride JB: **Substance use disorders in refugee and migrant groups in Sweden: A nationwide cohort study of 1.2 million people**. *Plos Medicine* 2019, **16**(11).
10. Stedman RC, Connelly NA, Heberlein TA, Decker DJ, Allred SB: **The End of the (Research) World As We Know It? Understanding and Coping With Declining Response Rates to Mail Surveys**. *Soc Natur Resour* 2019, **32**(10):1139-1154.
11. Platt B, O'Driscoll C, Curran VH, Rendell PG, Kamboj SK: **The effects of licit and illicit recreational drugs on prospective memory: a meta-analytic review**. *Psychopharmacology* 2019, **236**(4):1131-1143.
12. Allebeck P: **The use of population based registers in psychiatric research**. *Acta Psychiatr Scand* 2009, **120**(5):386-391.
13. Ludvigsson JF, Andersson E, Ekbom A, Feychting M, Kim JL, Reuterwall C, Heurgren M, Olausson PO: **External review and validation of the Swedish national inpatient register**. *BMC Public Health* 2011, **11**:450.
14. Hollander AC, Mackay E, Sjoqvist H, Kirkbride JB, Baarnhielm S, Dalman C: **Psychiatric care use among migrants to Sweden compared with Swedish-born residents: a longitudinal cohort study of 5 150 753 people**. *BMJ Glob Health* 2020, **5**(9).
15. Wandell P, Carlsson AC, Wettermark B, Lord G, Cars T, Ljunggren G: **Most common diseases diagnosed in primary care in Stockholm, Sweden, in 2011**. *Family Practice* 2013, **30**(5):506-513.
16. Manhica H, Gauffin K, Almqvist YB, Rostila M, Hjern A: **Hospital Admission and Criminality Associated with Substance Misuse in Young Refugees - A Swedish National Cohort Study**. *PLoS One* 2016, **11**(11):e0166066.
17. Salvatore JE, Larsson Lonn S, Sundquist J, Lichtenstein P, Sundquist K, Kendler KS: **Alcohol use disorder and divorce: evidence for a genetic correlation in a population-based Swedish sample**. *Addiction* 2017, **112**(4):586-593.
18. **www.socialstyrelsen.se**
19. **www.scb.se**
20. **https://www.forsakringskassan.se/statistik**
21. Bråttsförebyggande rådet B: **https://www.bra.se/bra-in-english/home.html** 2023.
22. Statistics Sweden S: **Longitudinell integrationsdatabas för sjukförsäkrings- och arbetsmarknadsstudier (LISA) 1990-2019 (Integrated database for labour market research)**. Stockholm: Statistics Sweden; 2019.
23. Sweden S: **Dokumentation of databasen STATIV**. In. Edited by Sweden S. www.sos.se; 2019.
24. World Health Organization: **International statistical classification of diseases and related health problems : ICD-10. Vol. 1 : tabular list**, 2. edn. Geneva: World Health Organization; 2004.
25. American Psychiatric Association., American Psychiatric Association. DSM-5 Task Force.: **Diagnostic and statistical manual of mental disorders DSM-5**. In., 5th edn. Arlington, VA: American Psychiatric Association,; 2013: 1 electronic resource.

For peer review only - http://bmjopen.bmj.com/site/about/guidelines.xhtml

26. Karriker-Jaffe KJ, Ji JG, Sundquist J, Kendler KS, Sundquist K: **Disparities in pharmacotherapy for alcohol use disorder in the context of universal health care: a Swedish register study**. *Addiction* 2017, **112**(8):1386-1394.
27. **https://www.fass.se/LIF/startpage**
28. DeBeck K, Shannon K, Wood E, Li K, Montaner J, Kerr T: **Income generating activities of people who inject drugs**. *Drug Alcohol Depend* 2007, **91**(1):50-56.
29. Moyle L, Coomber R: **Earning a Score: An Exploration of the Nature and Roles of Heroin and Crack Cocaine 'User-Dealers'**. *Brit J Criminol* 2015, **55**(3):534-555.

For peer review only

For peer review only - http://bmjopen.bmj.com/site/about/guidelines.xhtml

Supplementary tables

Supplementary table 1 Alcohol use disorder in the male population. Total number (N) with indication, percent % and number per cell (n) with register indication by age-group, migrant status, education, marital status, phi - the correlation coefficient between the registers and the unique contribution of the specific register to all registers combined (Any registers) for the following registers: Inpatient care, Outpatient care, the Register of Medications (Medications), the Social Insurance register (Social Insurance), the Register of convictions (Convictions), and Any register.

		**Inpatient**	**Outpatient**	**Medication**	**Social Insurance**	**Convictions**	**Any registers**
		N=46889	N=65445	N=71680	N=6415	N=1642	N=114789
		% (n)	% (n)	% (n)	% (n)	% (n)	% (n)
**Age**	**20–29**	1.09 (3627)	1.87 (6209)	1.45 (4821)	0.08 (259)	0.08 (251)	3.16 (10530)
	**30–39**	1.01 (6033)	1.73 (10297)	1.83 (10881)	0.15 (898)	0.06 (333)	2.98 (17699)
	**40–49**	1.69 (11050)	2.49 (16257)	2.90 (18887)	0.31 (2038)	0.07 (441)	4.26 (27768)
	**50–59**	2.37 (13889)	3.08(18097)	3.52 (20678)	0.40 (2320)	0.07 (396)	5.38 (31563)
	**60–65**	2.05 (12290)	2.43 (14585)	2.74 (16413)	0.15 (900)	0.04 (221)	4.54 (27229)
**Migration status**	**Born in Sweden**	1.73 (40370)	2.40 (55988)	2.74(63916)	0.25 (5748)	0.06 (1345)	4.27 (99770)
	**Born abroad**	1.63 (6133)	2.34 (8798)	1.94 (7295)	0.17 (632)	0.07 (275)	3.71 (13944)
	**Refugee**	0.69 (386)	1.18 (659)	0.84 (469)	0.06 (35)	0.04 (22)	1.92 (1075)
**Education**	**Education Pre-upper**	3.03 (15314)	3.88 (19623)	3.98 (20116)	0.33 (1671)	0.14 (687)	6.75 (34084)
	**Upper second**	1.83 (24993)	2.51 (34331)	2.79 (38145)	0.28 (3774)	0.06 (802)	4.43 (60676)
	**Post-second**	0.71 (6023)	1.28 (10749)	1.52 (12833)	0.11 (947)	0.01 (115)	2.23 (18772)
	**Miss**	1.14 (559)	1.51 (742)	1.19 (586)	0.05 (23)	0.08 (38)	2.56 (1257)
**Marital status**	**Married**	0.84 (9605)	1.29 (14734)	1.85 (21070)	0.13 (1453)	0.02 (267)	2.58 (29405)
	**Unmarried**	1.89 (24986)	2.69 (35455)	2.64 (34762)	0.25 (3311)	0.08 (992)	4.52 (59662)
	**Divorced**	4.07 (11824)	5.04 (14650)	5.22 (15183)	0.55 (1602)	0.13 (369)	8.48 (24635)
	**Widow(er)**	2.77 (474)	3.54 (606)	3.89 (665)	0.29 (49)	0.08 (14)	6.36 (1087)
**Unique contribution to Any register**	%	8.74 (10037)	16.55 (19001)	27.57 (31653)	0.46 (531)	0.19 (98)	-

For peer review only - http://bmjopen.bmj.com/site/about/guidelines.xhtml

Supplementary table 2 Alcohol use disorder in the female population. Total number (N) with indication, percent % and number per cell (n) with register indication by age-group, migrant status, education, marital status, phi - the correlation coefficient between the registers and the unique contribution of the specific register to all registers combined (Any registers) for the following registers: Inpatient care, Outpatient care, the Register of Medications (Medications), the Social Insurance register (Social Insurance), the Register of convictions (Convictions), and Any register.

		Inpatient	Outpatient	Medication	Social Insurance	Convictions	Any registers
		n=20051	n=30834	n=31519	n=2908	n=195	n=52389
**Age**	**20–29**	0.72 (2290)	1.28 (4073)	0.74 (2361)	0.04 (126)	0.01 (20)	1.99 (6317)
	**30–39**	0.53 (3020)	0.91 (5213)	0.86 (4899)	0.08 (447)	0.01 (44)	1.49 (8562)
	**40–49**	0.84 (5253)	1.30 (8173)	1.42 (8917)	0.16 (1017)	0.01 (64)	2.15 (13461)
	**50–59**	0.95 (5437)	1.39 (7984)	1.60 (9215)	0.17 (999)	0.01 (46)	2.42 (13885)
	**60–65**	0.68 (4051)	0.90 (5391)	1.03 (6127)	0.05 (319)	0.00 (21)	1.71 (10164)
**Migration status**	**Born in Sweden**	0.76 (16946)	1.17 (26229)	1.23 (27502)	0.12 (2581)	0.01 (182)	2.01 (44962)
	**Born abroad**	0.73 (3024)	1.08 (4458)	0.95 (3927)	0.08 (324)	0.00 (13)	1.74 (7204)
	**Refugee**	0.24 (81)	0.43 (147)	0.26 (90)	0.01 (3)	0.00 (0)	0.65 (223)
**Education**	**Education Pre-upper**	1.44 (5505)	1.94 (7409)	1.78 (6792)	0.13 (506)	0.02 (69)	3.24 (12375)
	**Upper second**	0.85 (10640)	1.28 (16093)	1.31 (16417)	0.13 (1692)	0.01 (84)	2.18 (27397)
	**Post-second**	0.37 (3697)	0.70 (7053)	0.80 (8096)	0.07 (702)	0.00 (40)	1.21 (12164)
	**Miss**	0.49 (209)	0.66 (279)	0.50 (214)	0.02 (8)	0.00 (2)	1.07 (453)
**Marital status**	**Married**	0.42 (5082)	0.66 (8031)	0.80 (9748)	0.07 (821)	0.00 (45)	1.22 (14853)
	**Unmarried**	0.82 (8644)	1.35 (14143)	1.20 (12556)	0.12 (1214)	0.01 (102)	2.17 (22792)
	**Divorced**	1.58 (5707)	2.18 (7911)	2.31 (8369)	0.23 (815)	0.01 (43)	3.69 (13352)
	**Widow(er)**	1.16 (618)	1.41 (749)	1.59 (846)	0.11 (58)	0.01 (5)	2.62 (1392)
**Unique contribution to Any register**	**%**	9.60 (5029)	20.09 (10527)	26.16 (13705)	0.53 (277)	0.19 (98)	-

For peer review only - http://bmjopen.bmj.com/site/about/guidelines.xhtml

Supplementary table 3 The association between socioeconomic factors and appearing in specific registers among men with an indication of alcohol use disorder (n= 114 789) by the following registers: the Inpatient care, the Outpatient care, the Register of Medications (Medications), the Social Insurance register (Social Insurance) and the Register of convictions (Convictions), presented with odds ratios (OR) with 95 % confidence interval (CI), by age group, sex, migrant status, education level and marital status all included in the multivariate models.

		Inpatient	Outpatient	Medication	Social Insurance	Convictions
**Age-group**	**20–29**	0.55 (0.53-0.58)	0.98 (0.94-1.03)	0.54 (0.52-0.57)	0.33 (0.29-0.38)	1.78 (1.49-2.11)
	**30–39**	0.60 (0.58-0.63)	0.98 (0.95-1.02)	0.94 (0.91-0.98)	0.69 (0.64-0.75)	1.55 (1.33-1.81)
	**40–49**	0.82 (0.80-0.85)	1.04 (1.01-1.08)	1.17 (1.13-1.21)	1.00 (0.94-1.07)	1.32 (1.15-1.52)
	**50–59**	1	1	1	1	1
	**60–64**	1.12 (1.08-1.15)	0.89 (0.86-0.92)	0.73 (0.71-0.76)	0.43 (0.4-0.47)	0.64 (0.54-0.76)
**Migrant status**	**Migrant**	1.15 (1.11-1.19)	1.35 (1.30-1.40)	0.60 (0.57-0.62)	0.78 (0.72-0.85)	1.4 (1.22-1.6)
	**Refugee**	0.93 (0.82-1.06)	1.24 (1.10-1.41)	0.37 (0.33-0.42)	0.51 (0.36-0.71)	1.39 (0.9-2.13)
	**Sweden**	1	1	1	1	1
**Education**	**Pre-upper secondary school**	1.14 (1.11-1.17)	1.03 (1.01-1.06)	0.92 (0.89-0.94)	0.84 (0.79-0.89)	1.55 (1.39-1.72)
	**Upper secondary**	1	1	1	1	1
	**Post-secondary school**	0.69 (0.67-0.72)	1.10 (1.06-1.13)	1.19 (1.15-1.24)	0.83 (0.77-0.9)	0.51(0.42-0.62)
	**Missing**	1.26 (1.12-1.42)	0.97 (0.86-1.09)	0.76 (0.68-0.86)	0.4 (0.26-0.6)	1.75 (1.25-2.46)
**Marital status**	**Married**	1	1	1	1	1
	**Single**	1.74 (1.69-1.80)	1.45 (1.41-1.50)	0.57 (0.55-0.59)	1.13 (1.06-1.21)	1.3 (1.12-1.5)
	**Divorced**	1.77 (1.71-1.84)	1.47 (1.42-1.52)	0.65 (0.63-0.68)	1.33 (1.24-1.44)	1.64 (1.4-1.92)
	**Widow/Widower**	1.36 (1.21-1.54)	1.33 (1.17-1.50)	0.72 (0.63-0.82)	1.19 (0.89-1.6)	1.72 (1-2.96)

For peer review only

Supplementary table 4 The association between socioeconomic factors and appearing in specific registers among women with an indication of alcohol use disorder (n= 52 389) by the following registers: the Inpatient care, the Outpatient care, the Register of Medications (Medications), the Social Insurance register (Social Insurance) and the Register of convictions (Convictions), presented with odds ratios (OR) with 95 % confidence interval (CI), by age group, sex, migrant status, education level and marital status all included in the multivariate models.

		**Inpatient**	**Outpatient**	**Medication**	**Social Insurance**	**Convictions**
**Age-group**	**20–29**	0.81 (0.76-0.87)	1.24 (1.16-1.33)	0.33 (0.31-0.35)	0.26 (0.22-0.32)	0.74 (0.42-1.3)
	**30–39**	0.85 (0.80-0.90)	1.11 (1.05-1.18)	0.70 (0.66-0.74)	0.71 (0.63-0.8)	1.39 (0.9-2.15)
	**40–49**	1.00 (0.95-1.05)	1.13 (1.08-1.19)	1.00 (0.95-1.06)	1.04 (0.95-1.14)	1.4 (0.95-2.06)
	**50–59**	1	1	1	1	1
	**60–64**	1.03 (0.98-1.09)	0.86 (0.82-0.91)	0.75 (0.71-0.79)	0.43 (0.38-0.49)	0.6 (0.36-1.02)
**Migrant status**	**Migrant**	1.16 (1.10-1.22)	1.19 (1.13-1.26)	0.71(0.67-0.75)	0.76 (0.68-0.86)	0.44 (0.25-0.77)
	**Refugee**	0.97 (0.73-1.27)	1.30 (0.99-1.72)	0.48 (0.36-0.63)	0.24 (0.08-0.75)	-
	**Sweden**	1	1	1	1	1
**Education**	**Pre-upper secondary school**	1.26 (1.21-1.31)	1.04 (1.00-1.09)	0.87 (0.84-0.91)	0.7 (0.63-0.78)	1.93 (1.4-2.66)
	**Upper secondary**	1	1	1	1	1
	**Post-secondary school**	0.69 (0.66-0.72)	1.01 (0.97-1.05)	1.27 (1.21-1.33)	0.93 (0.85-1.02)	1.11(0.76-1.63)
	**Missing**	1.40 (1.16-1.69)	0.98 (0.81-1.19)	0.95 (0.78-1.15)	0.42 (0.21-0.85)	2.1 (0.51-8.7)
**Marital status**	**Married**	1	1	1	1	1
	**Single**	1.23 (1.17-1.29)	1.27 (1.21-1.33)	0.83 (0.79-0.87)	1.09 (0.99-1.2)	1.29 (0.89-1.88)
	**Divorced**	1.37 (1.31-1.44)	1.24 (1.18-1.30)	0.87 (0.83-0.92)	1.1 (1-1.22)	1.06 (0.7-1.62)
	**Widow/Widower**	1.42 (1.27-1.59)	1.08 (0.96-1.20)	0.87 (0.78-0.98)	0.95 (0.72-1.26)	1.48 (0.58-3.8)

For peer review only

Supplementary table 5 Drug use disorder in the male population. Total number (N) with indication, percent % and number per cell (n) with register indication by age-group, migrant status, education, marital status, phi - the correlation coefficient between the registers and the unique contribution of the specific register to all registers combined (Any registers) for the following registers: Inpatient care, Outpatient care, the Register of Medications (Medications), the Social Insurance register (Social Insurance), the Register of convictions (Convictions), and Any register.

	**Inpatient**	**Outpatient**	**Medication**	**Social Insurance**	**Convictions**	**Any registers**
	n= 20365	n= 29671	n= 8117	n= 2449	n= 48155	n= 69928
**20–29**	1.60 (5314)	2.33 (7740)	0.26 (875)	0.23 (777)	5.30 (17643)	6.28 (20889)
**30–39**	0.98 (5808)	1.44 (8551)	0.33 (1979)	0.15 (877)	2.48 (14738)	3.25 (19323)
**40–49**	0.73 (4744)	1.07 (6964)	0.30 (1951)	0.08 (495)	1.43 (9331)	2.25 (14642)
**50–59**	0.55 (3258)	0.81 (4728)	0.31 (1793)	0.04 (264)	0.94 (5511)	1.81 (10620)
**60–65**	0.21 (1241)	0.28 (1688)	0.25 (1519)	0.01 (36)	0.16 (932)	0.74 (4454)
**Born in Sweden**	0.72 (16895)	1.03 (24099)	0.28 (6545)	0.09 (2115)	1.56 (36426)	2.33 (54457)
**Born abroad**	0.77 (2908)	1.22 (4574)	0.32 (1210)	0.08 (286)	2.66 (9978)	3.48 (13084)
**Refugee**	1.00 (562)	1.78 (998)	0.65 (362)	0.09 (48)	3.13 (1751)	4.27 (2387)
**Education Pre-upper**	1.74 (8767)	2.42 (12204)	0.64 (3231)	0.16 (819)	3.97 (20080)	5.31 (26807)
**Upper second**	0.70 (9593)	1.04 (14240)	0.28 (3842)	0.10 (1364)	1.76 (24123)	2.60 (35597)
**Post-second**	0.19 (1605)	0.32 (2671)	0.11 (954)	0.03 (246)	0.32 (2725)	0.71 (5994)
**Miss**	0.81 (400)	1.13 (556)	0.18 (90)	0.04 (20)	2.50 (1227)	3.11 (1530)
**Married**	0.20 (2321)	0.31 (3558)	0.19 (2176)	0.02 (271)	0.36 (4067)	0.79 (9019)
**Unmarried**	1.15 (15198)	1.67 (22078)	0.35 (4569)	0.15 (1921)	2.95 (3891)	3.93 (51823)
**Divorsed**	0.95 (2771)	1.35 (3927)	0.45 (1307)	0.09 (251)	1.75 (5100)	3.05 (8861)
**Widow(er)**	0.44 (75)	0.63 (108	0.38 (65)	0.04 (6)	0.44 (76)	1.32 (225)
**Unique contribution to Any register**	5.35 (3744)	11.92 (8332)	4.48 (3131)	0.39 (273)	44.08 (30825)	

For peer review only - http://bmjopen.bmj.com/site/about/guidelines.xhtml

Supplementary table 6 Drug use disorder in the female population. Total number (N) with indication, percent % and number per cell (n) with register indication by age-group, migrant status, education, marital status, phi - the correlation coefficient between the registers and the unique contribution of the specific register to all registers combined (Any registers) for the following registers: Inpatient care, Outpatient care, the Register of Medications (Medications), the Social Insurance register (Social Insurance), the Register of convictions (Convictions), and Any register.

	**Inpatient**	**Outpatient**	**Medication**	**Social Insurance**	**Convictions**	**Any registers**
	n= 11810	n= 16452	n= 5176	n= 1110	n= 9056	n= 29624
**20–29**	0.81 (2565)	1.06 (3363)	0.15 (479)	0.08 (255)	0.99 (3145)	1.92 (6095)
**30–39**	0.50 (2861)	0.70 (4020)	0.16 (912)	0.06 (338)	0.43 (2459)	1.17 (6703)
**40–49**	0.48 (3022)	0.68 (4237)	0.19 (1203)	0.05 (319)	0.35 (2196)	1.18 (7418)
**50–59**	0.36 (2084)	0.54 (3083)	0.22 (1281)	0.03 (158)	0.19 (1115)	0.99 (5659)
**60–65**	0.21 (1278)	0.29 (1749)	0.22 (1301)	0.01 (40)	0.02 (141)	0.63 (3749)
**Born in Sweden**	0.45 (10002)	0.62 (13989)	0.20 (4539)	0.04 (993)	0.35 (7910)	1.12 (25177)
**Born abroad**	0.40 (1677)	0.55 (2292)	0.14 (595)	0.03 (110)	0.25 (1054)	1.00 (4125)
**Refugee**	0.38 (131)	0.50 (171)	0.12 (42)	0.02 (7)	0.27 (92)	0.94 (322)
**Education Pre-upper**	1.07 (4100)	1.44 (5498)	0.42 (1621)	0.07 (255)	1.09 (4170)	2.55 (9750)
**Upper second**	0.45 (5621)	0.62 (7830)	0.20 (2458)	0.05 (579)	0.32 (4074)	1.15 (14422)
**Post-second**	0.19 (1870)	0.28 (2808)	0.10 (1035)	0.03 (270)	0.06 (618)	0.49 (4954)
**Miss**	0.52 (219)	0.74 (316)	0.15 (62)	0.01 (6)	0.46 (194)	1.17 (498)
**Married**	0.21 (2575)	0.29 (3601)	0.15 (1854)	0.02 (242)	0.08 (1000)	0.58 (7031)
**Unmarried**	0.60 (6312)	0.85 (8911)	0.19 (2034)	0.06 (677)	0.60 (6305)	1.48 (15566)
**Divorsed**	0.76 (2737)	1.02 (3680)	0.31 (1126)	0.05 (181)	0.46 (1662)	1.80 (6533)
**Widow(er)**	0.35 (186)	0.49 (260)	0.30 (162)	0.02 (10)	0.17 (89)	0.93 (494)
**Unique contribution to Any register**	14.20 (4204)	25.60 (7583)	10.64 (3152)	0.79 (233)	16.53 (4898)	--

For peer review only - http://bmjopen.bmj.com/site/about/guidelines.xhtml

Supplementary table 7 The association between socioeconomic factors and appearing in specific registers among men with an indication of drug use disorder (n= 69 928) by the following registers: the Inpatient care, the Outpatient care, the Register of Medications (Medications), the Social Insurance register (Social Insurance) and the Register of convictions (Convictions), presented with odds ratios (OR) with 95 % confidence interval (CI), by age group, sex, migrant status, education level and marital status all included in the multivariate models.

		**Inpatient**	**Outpatient**	**Medication**	**Social Insurance**	**Convictions**
		OR (95 % CI)	OR (95 % CI)	OR (95 % CI)	OR (95 % CI)	OR (95 % CI)
**Age-group**	**20–29**	0.73 (0.69-0.77)	0.70 (0.67-0.74)	0.24 (0.22-0.27)	1.77 (1.52-2.07)	4.07 (3.84-4.31)
	**30–39**	0.96 (0.91-1.02)	0.98 (0.93-1.03)	0.60 (0.55-0.64)	2.08 (1.80-2.41)	2.72 (2.58-2.87)
	**40–49**	1.10 (1.04-1.16)	1.14 (1.08-1.20)	0.77 (0.72-0.83)	1.44 (1.24-1.68)	1.57 (1.49-1.65)
	**50–59**	1	1	1	1	1
	**60–65**	0.86 (0.80-0.93)	0.75 (0.69-0.80)	2.22 (2.05-2.42)	0.29 (0.21-0.42)	0.28 (0.26-0.31)
**Migrant status**	**Born abroad**	0.62 (0.59-0.65)	0.67 (0.64-0.69)	0.73 (0.69-0.79)	0.54 (0.47-0.61)	1.69 (1.61-1.78)
	**Refugee**	0.67 (0.61-0.74)	0.88 (0.81-0.96)	1.27 (1.13-1.43)	0.49 (0.37-0.66)	1.60 (1.45-1.77)
	**Sweden**	1	1	1	1	1
**Education**	**Pre-upper secondary school**	1.39 (1.34-1.44)	1.32 (1.28-1.37)	1.28 (1.22-1.35)	0.79 (0.72-0.86)	1.30 (1.25-1.35)
	**Upper secondary**	1	1	1	1	1
	**Post-secondary school**	1.02 (0.96-1.09)	1.24 (1.17-1.31)	1.13 (1.04-1.22)	1.21 (1.05-1.39)	0.48 (0.45-0.51)
	**Missing**	1.33 (1.18-1.50)	1.16 (1.04-1.29)	0.77 (0.62-0.96)	0.40 (0.25-0.62)	1.21 (1.06-1.39)
**Marital status**	**Married**	1	1	1	1	1
	**Single**	1.18 (1.12-1.25)	1.17 (1.11-1.23)	0.50 (0.47-0.54)	0.86 (0.75-0.98)	2.16 (2.05-2.28)
	**Divorced**	1.24 (1.17-1.33)	1.17 (1.10-1.25)	0.48 (0.45-0.52)	1.01(0.85-1.21)	2.09 (1.96-2.23)
	**Widow/Widower**	1.34 (1.01-1.78)	1.40 (1.08-1.84)	0.83 (0.61-1.12)	1.32 (0.58-3.02)	1.17 (0.86-1.58)

For peer review only

Supplementary table 8 The association between socioeconomic factors and appearing in specific registers among women with an indication of drug use disorder (n= 29 624) by the following registers: the Inpatient care, the Outpatient care, the Register of Medications (Medications), the Social Insurance register (Social Insurance) and the Register of convictions (Convictions), presented with odds ratios (OR) with 95 % confidence interval (CI), by age group, sex, migrant status, education level and marital status all included in the multivariate models.

		**Inpatient**	**Outpatient**	**Medication**	**Social Insurance**	**Convictions**
		OR (95 % CI)	OR (95 % CI)	OR (95 % CI)	OR (95 % CI)	OR (95 % CI)
**Age-group**	**20–29**	1.29 (1.19-1.41)	0.99 (0.91-1.08)	0.31 (0.27-0.35)	1.71 (1.37-2.14)	3.16 (2.88-3.47)
	**30–39**	1.33 (1.23-1.44)	1.24 (1.15-1.34)	0.54 (0.49-0.60)	1.88 (1.53-2.30)	2.16 (1.97-2.36)
	**40–49**	1.20 (1.11-1.29)	1.12 (1.04-1.20)	0.67 (0.61-0.73)	1.59 (1.31-1.94)	1.65 (1.52-1.80)
	**50–59**	1	1	1	1	1
	**60–65**	0.89 (0.81-0.97)	0.74 (0.68-0.81)	1.69 (1.54-1.86)	0.37 (0.26-0.53)	0.17 (0.14-0.20)
**Migrant status**	**Migrant**	1.02 (0.95-1.09)	0.98 (0.92-1.05)	0.73 (0.66-0.80)	0.69 (0.57-0.85)	0.79 (0.73-0.86)
	**Refugee**	0.99 (0.79-1.24)	0.88 (0.70-1.10)	0.78 (0.56-1.09)	0.52 (0.24-1.10)	0.77 (0.60-1.00)
	**Sweden**	1	1	1	1	1
**Education**	**Pre-upper secondary school**	1.11 (1.06-1.17)	1.08 (1.03-1.14)	1.09 (1.01-1.17)	0.62 (0.54-0.72)	1.79 (1.69-1.90)
	**Upper secondary**	1	1	1	1	1
	**Post-secondary school**	0.98 (0.92-1.05)	1.14 (1.07-1.22)	1.10 (1.01-1.20)	1.51 (1.30-1.75)	0.42 (0.38-0.47)
	**Missing**	1.17 (0.97-1.41)	1.49 (1.23-1.79)	1.12 (0.85-1.48)	0.29 (0.13-0.64)	1.27 (1.05-1.55)
**Marital status**	**Married**	1	1	1	1	1
	**Single**	1.04 (0.98-1.11)	1.19 (1.12-1.27)	0.66 (0.61-0.71)	1.01 (0.86-1.19)	2.25 (2.07-2.44)
	**Divorced**	1.27 (1.18-1.36)	1.25 (1.16-1.33)	0.55 (0.50-0.60)	0.88 (0.72-1.07)	2.19 (1.99-2.40)
	**Widow/Widower**	1.16 (0.96-1.40)	1.20 (1.00-1.45)	0.97 (0.80-1.19)	0.93 (0.49-1.77)	2.38 (1.83-3.10)

For peer review only - http://bmjopen.bmj.com/site/about/guidelines.xhtml

The RECORD statement – checklist of items, extended from the STROBE statement, that should be reported in observational studies using routinely collected health data.

	Item No.	STROBE items	Location in manuscript where items are reported	RECORD items	Location in manuscript where items are reported
**Title and abstract**					
	1	(a) Indicate the study's design with a commonly used term in the title or the abstract (b) Provide in the abstract an informative and balanced summary of what was done and what was found	Title page at page 1 and abstract at page 2.	RECORD 1.1: The type of data used should be specified in the title or abstract. When possible, the name of the databases used should be included.<br><br>RECORD 1.2: If applicable, the geographic region and timeframe within which the study took place should be reported in the title or abstract.<br><br>RECORD 1.3: If linkage between databases was conducted for the study, this should be clearly stated in the title or abstract.	Title page at page 1 and abstract at page 2.
**Introduction**					
Background rationale	2	Explain the scientific background and rationale for the investigation being reported	Page 4		
Objectives	3	State specific objectives, including any prespecified hypotheses	Page 4		
**Methods**					
Study Design	4	Present key elements of study design early in the paper	Page 4		
Setting	5	Describe the setting, locations, and relevant dates, including periods of recruitment, exposure, follow-up, and data collection	Page 5		

For peer review only

Participants	6	*(a) Cohort study* - Give the eligibility criteria, and the sources and methods of selection of participants. Describe methods of follow-up<br>*Case-control study* - Give the eligibility criteria, and the sources and methods of case ascertainment and control selection. Give the rationale for the choice of cases and controls<br>*Cross-sectional study* - Give the eligibility criteria, and the sources and methods of selection of participants<br><br>*(b) Cohort study* - For matched studies, give matching criteria and number of exposed and unexposed<br>*Case-control study* - For matched studies, give matching criteria and the number of controls per case		RECORD 6.1: The methods of study population selection (such as codes or algorithms used to identify subjects) should be listed in detail. If this is not possible, an explanation should be provided.<br><br>RECORD 6.2: Any validation studies of the codes or algorithms used to select the population should be referenced. If validation was conducted for this study and not published elsewhere, detailed methods and results should be provided.<br><br>RECORD 6.3: If the study involved linkage of databases, consider use of a flow diagram or other graphical display to demonstrate the data linkage process, including the number of individuals with linked data at each stage.	Page 5<br><br>Page 5<br><br>Page 5
Variables	7	Clearly define all outcomes, exposures, predictors, potential confounders, and effect modifiers. Give diagnostic criteria, if applicable.	Page 5-6	RECORD 7.1: A complete list of codes and algorithms used to classify exposures, outcomes, confounders, and effect modifiers should be provided. If these cannot be reported, an explanation should be provided.	Page 5-6
Data sources/ measurement	8	For each variable of interest, give sources of data and details of methods of assessment (measurement).<br>Describe comparability of assessment methods if there is more than one group	Page 5-6		

For peer review only

Bias	9	Describe any efforts to address potential sources of bias	Page 5-6		
Study size	10	Explain how the study size was arrived at	page 5		
Quantitative variables	11	Explain how quantitative variables were handled in the analyses. If applicable, describe which groupings were chosen, and why	page 5-6		
Statistical methods	12	(a) Describe all statistical methods, including those used to control for confounding<br>(b) Describe any methods used to examine subgroups and interactions<br>(c) Explain how missing data were addressed<br>(d) *Cohort study* - If applicable, explain how loss to follow-up was addressed<br>*Case-control study* - If applicable, explain how matching of cases and controls was addressed<br>*Cross-sectional study* - If applicable, describe analytical methods taking account of sampling strategy<br>(e) Describe any sensitivity analyses	Page 7		
Data access and cleaning methods		..		RECORD 12.1: Authors should describe the extent to which the investigators had access to the database population used to create the study population.	Page 5-6

For peer review only

				RECORD 12.2: Authors should provide information on the data cleaning methods used in the study.	Page 5-6
Linkage		..		RECORD 12.3: State whether the study included person-level, institutional-level, or other data linkage across two or more databases. The methods of linkage and methods of linkage quality evaluation should be provided.	Page 5-6
**Results**					
Participants	13	(a) Report the numbers of individuals at each stage of the study (*e.g.*, numbers potentially eligible, examined for eligibility, confirmed eligible, included in the study, completing follow-up, and analysed)<br>(b) Give reasons for non-participation at each stage.<br>(c) Consider use of a flow diagram	Page 7	RECORD 13.1: Describe in detail the selection of the persons included in the study (*i.e.*, study population selection) including filtering based on data quality, data availability and linkage. The selection of included persons can be described in the text and/or by means of the study flow diagram.	Page 7
Descriptive data	14	(a) Give characteristics of study participants (*e.g.*, demographic, clinical, social) and information on exposures and potential confounders<br>(b) Indicate the number of participants with missing data for each variable of interest<br>(c) *Cohort study* - summarise follow-up time (*e.g.*, average and total amount)	page 7		
Outcome data	15	*Cohort study* - Report numbers of outcome events or summary measures over time<br>*Case-control study* - Report numbers in each exposure	Page 7		

For peer review only

		category, or summary measures of exposure<br>*Cross-sectional study* - Report numbers of outcome events or summary measures			
Main results	16	(a) Give unadjusted estimates and, if applicable, confounder-adjusted estimates and their precision (e.g., 95% confidence interval). Make clear which confounders were adjusted for and why they were included<br>(b) Report category boundaries when continuous variables were categorized<br>(c) If relevant, consider translating estimates of relative risk into absolute risk for a meaningful time period	page 11-12		
Other analyses	17	Report other analyses done—e.g., analyses of subgroups and interactions, and sensitivity analyses	Page 11-12		
**Discussion**					
Key results	18	Summarise key results with reference to study objectives	Page 13		
Limitations	19	Discuss limitations of the study, taking into account sources of potential bias or imprecision. Discuss both direction and magnitude of any potential bias	Page 14	RECORD 19.1: Discuss the implications of using data that were not created or collected to answer the specific research question(s). Include discussion of misclassification bias, unmeasured confounding, missing data, and changing eligibility over time, as they pertain to the study being reported.	Page 14
Interpretation	20	Give a cautious overall interpretation of results considering objectives,	Page 13		

For peer review only

		limitations, multiplicity of analyses, results from similar studies, and other relevant evidence			
Generalisability	21	Discuss the generalisability (external validity) of the study results	Page 14		
**Other Information**					
Funding	22	Give the source of funding and the role of the funders for the present study and, if applicable, for the original study on which the present article is based	Page 16		
Accessibility of protocol, raw data, and programming code		..	Page 16	RECORD 22.1: Authors should provide information on how to access any supplemental information such as the study protocol, raw data, or programming code.	Page 16

*Reference: Benchimol EI, Smeeth L, Guttmann A, Harron K, Moher D, Petersen I, Sørensen HT, von Elm E, Langan SM, the RECORD Working Committee. The REporting of studies Conducted using Observational Routinely-collected health Data (RECORD) Statement. *PLoS Medicine* 2015; in press.

*Checklist is protected under Creative Commons Attribution (CC BY) license.

For peer review only
